# Multiscale modeling of T cell exhaustion: A mathematical framework integrating continuous dynamics with spatial heterogeneity

**DOI:** 10.1371/journal.pcbi.1014690

**Published:** 2026-08-26

**Authors:** Chenghang Li, Yuhong Zhang, Xue Liu, Yipu Qu, Xiulan Lai, Jinzhi Lei

**Affiliations:** 1 School of Mathematical Sciences, Tiangong University, Tianjin, China; 2 School of Software, Tiangong University, Tianjin, China; 3 School of Mathematics, Shandong University, Jinan, China; 4 School of Mathematics, Renmin University of China, Beijing, China; 5 Center for Applied Mathematics, Tiangong University, Tianjin, China; University of Pisa: Universita degli Studi di Pisa, ITALY

## Abstract

Continuous antigen exposure drives T cells into a progressive state of dysfunction known as exhaustion, enabling tumors to evade immune surveillance and promoting disease progression. Despite its importance, predictive modeling of T cell exhaustion remains a major challenge due to the complexity of its regulatory dynamics. To address this challenge, we developed a mathematical framework that characterizes the dynamic regulation of T cell exhaustion and its impact on tumor-immune interactions. Here, we integrate multi-source data, population dynamics modeling, and agent-based modeling to track the progressive stages of CD8+ T cell exhaustion. Our model demonstrates that immune checkpoint blockade significantly delays exhaustion and promotes the expansion of tumor-reactive T cells compared to untreated conditions. From a pseudo-potential energy perspective, we show that the core mechanism of immunotherapy lies in expanding the tumor-reactive T cell pool, which consequently reduces the overall state of exhaustion within the system. We find that T cell activation and exhaustion signals jointly govern tumor-immune dynamics. Enhancing activation alone without restricting exhaustion can inadvertently accelerate the loss of T cell function. In contrast, combining enhanced activation (via anti-CTLA-4) with suppressed exhaustion (via anti-PD-1) is essential for achieving a sustained antitumor response. Furthermore, spatial simulations confirm that a high-activation and low-exhaustion state effectively restricts tumor spread, maintaining substantially lower tumor densities compared to low-activation, high-exhaustion scenarios. Our framework provides quantitative insights into T cell exhaustion and a theoretical foundation for optimizing combination immunotherapies.

## Author summary

T cell exhaustion, driven by prolonged antigen exposure in chronic infections and cancer, progressively impairs T cell function and increases inhibitory receptor expression. Notably, PD-1/PD-L1 blockades can reinvigorate partially exhausted T cell subsets, offering a key clinical strategy to improve the efficacy of immune checkpoint therapies. In this study, we develop a mathematical model that captures the detailed interactions between five distinct T cell subtypes and tumor cells to investigate exhaustion dynamics in cancer. Our results illustrate how immunotherapeutic interventions reshape the tumor-immune landscape. By simulating combination therapies, we demonstrate that optimal treatment outcomes depend critically on the balance between T cell activation and exhaustion. Different activation-exhaustion profiles lead to qualitatively different outcomes in tumor control. Our framework provides quantitative insights into these complex dynamics, offering a solid theoretical basis to guide the design of more effective combination immunotherapies.

## Introduction

T cell exhaustion operates as a profound state of CD8+ T cell dysfunction that inherently arises during chronic infections and tumor progression [1–3]. Within the highly suppressive tumor microenvironment, malignancies actively engineer this dysfunctional state by deploying multiple resistance strategies, including persistent antigen exposure, the localized secretion of immunosuppressive cytokines, and the compensatory upregulation of distinct immune checkpoint molecules such as PD-1, CTLA-4, and TIM-3 [3–5]. Phenotypically and functionally, exhausted T cells are defined by the sustained co-expression of inhibitory receptors, a precipitous decline in effector cytokine production, and severely diminished proliferative capacity [1–5]. Ultimately, these systemic functional defects critically paralyze the immune network’s ability to consistently recognize and eradicate malignant clones, thereby structurally establishing T cell exhaustion as a driving engine of tumor immune evasion and a formidable barrier to successful precision immunotherapy [6–8].

Based on the distinct expression patterns of marker genes, exhausted CD8+ T cells are generally classified into two major subpopulations: progenitor and terminally exhausted T cells [3–5]. The linear differentiation model proposes that naïve CD8+ T cells first differentiate into tumor-reactive T cells; upon persistent antigenic stimulation, they progressively transition through a progenitor exhausted state before eventually acquiring a terminally exhausted phenotype [1,2,9]. This gradual loss of effector function is largely driven by the sustained upregulation of inhibitory receptors, particularly PD-1 [10–13]. Notably, PD-1 blockade has been shown to partially reverse T cell exhaustion, thereby reinvigorating antitumor immunity and expanding therapeutic options in cancer immunotherapy [12,14]. Consequently, reversing T cell exhaustion has become a central paradigm in developing effective cancer immunotherapies, making it crucial to understand its underlying dynamics to optimize treatment strategies.

Mathematical models have been extensively employed to investigate tumor-immune interactions [15–18]. For instance, Kreger et al. [19] developed a delay differential equation model to characterize the dynamics between tumors and myeloid-derived suppressor cells (MDSCs). More recently, computational frameworks in this area have become increasingly comprehensive. Li et al. [20] formulated a quantitative cancer-immunity cycle (QCIC) model to simulate core anti-tumor mechanisms. In parallel, Lin et al. [21] established a multiscale framework to elucidate tumor–macrophage interactions underlying immunotherapy resistance in glioblastoma, whereas Liu et al. [22] leveraged multiscale model-informed reinforcement learning to optimize combination treatment scheduling in glioblastoma. Additionally, Li et al. [23] established a rigorous mathematical framework integrating a nonlocal reaction-diffusion model with spatial transcriptomics data to uncover key determinants of intratumor phenotypic diversity. Despite these advances, relatively few studies have specifically focused on the quantitative characterization of T cell exhaustion.

To address this gap, several targeted modeling efforts have emerged. Ildefonso and Finley [24] developed a data-driven Boolean model to analyze how complex gene regulatory networks contribute to T cell exhaustion. At the population level, Simmons and Levy [25] constructed an ordinary differential equation (ODE) model describing interactions among tumors, cytotoxic T lymphocytes, and exhausted T cell subsets. Similarly, Beck et al. [26] designed an ODE model integrating multimodal in vivo data to quantify tumor-CTL interactions, revealing that IFNG‑mediated cytostatic effects dominate cytotoxicity, and identifying LAG3 and HAVCR2 as primary correlates of exhaustion, with PD‑1/PD‑L1 playing a more minor role in their specific context. Lai et al. [27] developed mathematical models stratified by the degree of T cell exhaustion, illustrating how exhaustion dynamically influences tumor growth. In a distinct macroscopic approach, Lai and Yu [28] recently explored the bistable regulatory mechanism underlying the interplay between tumor PD-L1 expression and T-cell exhaustion. In the context of engineered therapies, Sahoo et al. [29] verified that antigen burden and cell dosage markedly modulate the progression of CAR‑T cell exhaustion. Furthermore, by incorporating a pharmacokinetic module, Kareva et al. [30] explored drug resistance mechanisms driven by immune exhaustion.

Collectively, these studies lay a solid foundation for the quantitative investigation of T cell exhaustion; however, existing modeling strategies have several methodological limitations. Because they rely on discrete logical rules, Boolean models often struggle to capture the continuous dynamical transitions throughout exhaustion development. Standard ODE models typically adopt homogeneous population assumptions, ignoring both the gradual nature of exhaustion shifts and local spatial heterogeneity. Additionally, purely deterministic formulations exclude stochastic events, limiting their ability to reproduce the random fluctuations inherent to immune responses. Critically, because T cell exhaustion is driven by persistent antigen stimulation—a biological memory effect—accurately modeling this process requires memory-based integral terms to quantify cumulative historical inhibitory signals. Currently, few models can simultaneously incorporate continuous dynamics, spatial heterogeneity, stochastic evolution, and historical memory within a unified mathematical framework. Given the clinical importance of immune checkpoint inhibitors in reversing T cell exhaustion, filling this critical modeling gap is essential to better dissect exhaustion progression and optimize therapeutic schedules.

**Fig 1:**
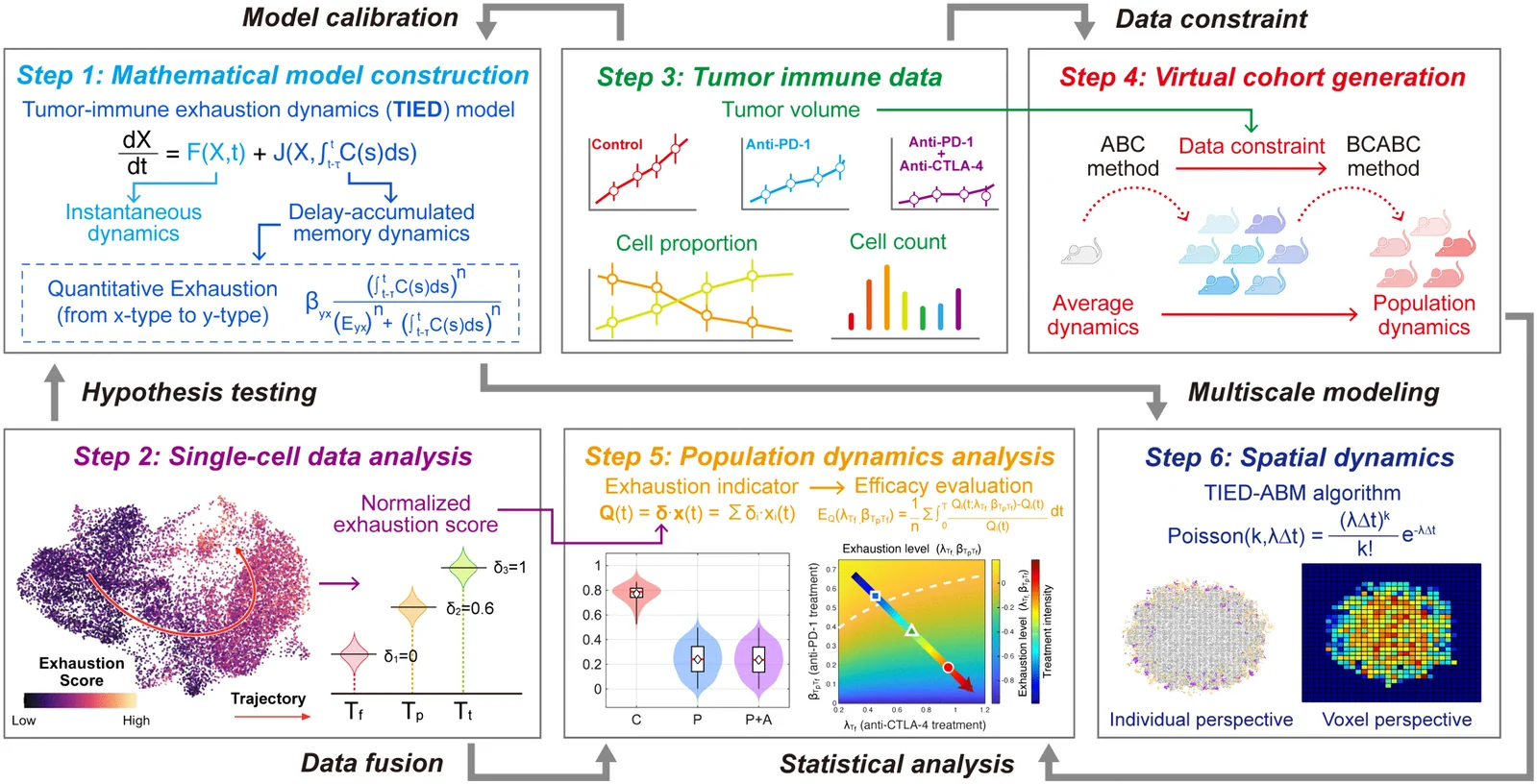
Multiscale framework for quantitative analysis of T cell exhaustion. The six steps illustrate the integration of mathematical modeling (incorporating a delay-accumulated memory term, Step 1), single-cell data analysis (validating the exhaustion lineage, Step 2), experimental data collection and calibration (tumor volume, cell proportions, and counts, Step 3), virtual cohort generation (BCABC method, Step 4), population dynamics analysis (fusion of scRNA-seq and model exhaustion indicators, Step 5), and spatial agent-based simulation (TIED-ABM algorithm, Step 6). Thick gray lines denote main inter-module connections, while colored thin lines represent intra-module mechanisms or data flows.

To systematically dissect the mechanisms driving T cell exhaustion and to address the aforementioned modeling limitations, we developed a multiscale analytical framework (Fig 1). First, we constructed a macroscopic tumor‑immune exhaustion dynamics (TIED) model (Step 1). Second, we analyzed single‑cell RNA sequencing (scRNA‑seq) data to biologically validate the continuous differentiation spectrum of T cell exhaustion, enabling robust hypothesis testing of phenotypic transitions in our model (Step 2). Third, we calibrated the TIED model using experimental measurements of tumor volume, cell proportions, and absolute cell counts (Step 3). Building on this deterministic foundation, we developed a boundary‑constrained approximate Bayesian computation (BCABC) method, utilizing tumor volume data as constraints to generate a highly representative virtual mouse cohort (Step 4). We then mapped exhaustion scores derived from the scRNA‑seq data onto the model’s exhaustion indicators to quantitatively evaluate the evolutionary trajectory of exhaustion under various therapeutic interventions (Step 5). Finally, we designed the TIED-agent‑based model (TIED‑ABM) to explicitly simulate the spatial progression of exhaustion within the tumor microenvironment (Step 6).

Our proposed framework introduces several key methodological innovations. First, unlike conventional models that assume instantaneous state transitions, we incorporated a time‑delay memory integral into the TIED model to quantify the cumulative effect of prolonged antigen exposure, thereby accurately capturing the continuous nature of T cell exhaustion. Second, we achieved a rigorous multi-level calibration by combining macroscopic in vivo measurements with microscopic scRNA‑seq data, providing reliable quantitative indicators for tracking exhaustion dynamics. Third, the newly developed BCABC method effectively bridges experimental observations with model simulations; by generating a data-constrained virtual cohort, it enables robust, population‑level analyses of exhaustion under diverse treatment strategies. Fourth, our TIED‑ABM algorithm translates macroscopic continuous rates into cell‑level stochastic events via spatial Poisson processes, successfully reproducing both the spatial heterogeneity and stochastic evolution of exhausted T cells. By unifying continuous population dynamics, spatial interactions, stochasticity, and biological memory, this framework offers a powerful computational platform for advancing the quantitative study of T cell exhaustion and optimizing combination immunotherapy.

## Methods

### The TIED model: A mechanistic framework for tumor‑immune exhaustion dynamics

**Fig 2:**
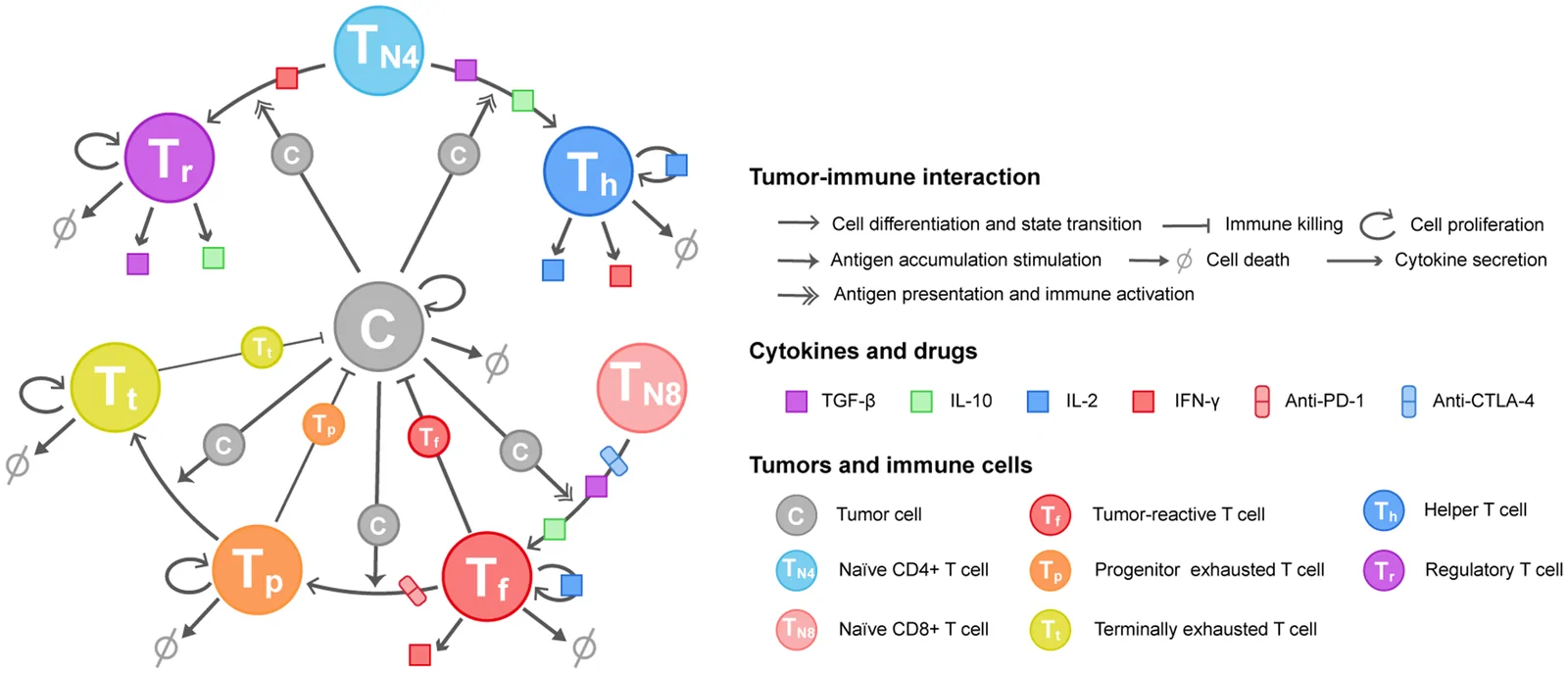
Dynamic regulatory network of tumor-immune interactions driving T cell exhaustion. *C*: tumor cells; $T_{N8}$: naïve CD8+ T cells; $T_{N4}$: naïve CD4+ T cells; $T_{f}$: tumor-reactive T cells; $T_{p}$: progenitor exhausted T cells; $T_{t}$: terminally exhausted T cells; $T_{h}$: helper T cells; $T_{r}$: regulatory T cells.

To elucidate the critical role of T cell exhaustion in tumor‑immune interactions, we developed a mechanistic mathematical model using delay integro-differential equations. We refer to this mathematical model as the tumor‑immune exhaustion dynamics (TIED) model. This nomenclature highlights its two core features: (i) tumor‑immune interactions and (ii) T cell exhaustion dynamics. Guided by established biological mechanisms, we constructed a regulatory network comprising tumor cells (*C*), tumor-reactive T cells ($T_{f}$), progenitor exhausted T cells ($T_{p}$), terminally exhausted T cells ($T_{t}$), helper T cells ($T_{h}$), and regulatory T cells ($T_{r}$) (Fig 2). This network further incorporates key cytokine‑mediated interactions: IL-2 promotes the proliferation of $T_{h}$ and $T_{f}$ [31,32]; IFN-γ inhibits $T_{r}$ activation [33]; and IL-10 and TGF-β suppress $T_{h}$ and $T_{f}$ activation [34]. Because cytokines primarily mediate intercellular communication, we represented the cytokine dynamics as functional regulatory expressions from secreting cells to target cells, thereby bridging molecular mechanisms with cellular dynamics. Notably, rather than explicitly modeling the temporal dynamics of cytokine concentrations, we incorporated their effects indirectly through the regulatory actions exerted by the associated cells, as formulated in the specific biological processes below.

(i) **Tumor evolution dynamics.** The proliferation of tumor cells (*C*) is driven by logistic growth and governed by the following equation:

$$\begin{array}{c} \frac{dC}{dt}=\underset{proliferation}{\underbrace{\alpha_{C}\left(1-\frac{C}{K_{C}}\right)C}}-\underset{killing}{\underbrace{\left(\eta_{T_{f}}T_{f}+\eta_{T_{p}}T_{p}+\eta_{T_{t}}T_{t}\right)C}}-\underset{death}{\underbrace{d_{C}C}}, \end{array}$$ (1)

where $\alpha_{C}$, $K_{C}$, and $d_{C}$ represent the intrinsic proliferation rate, carrying capacity, and natural death rate of tumor cells, respectively; $\eta_{T_{f}}$, $\eta_{T_{p}}$, and $\eta_{T_{t}}$ denote the tumor killing rates mediated by $T_{f}$, $T_{p}$, and $T_{t}$, respectively.

(ii) **CD8+ T cell evolution dynamics.** To quantitatively characterize the progression of T cell exhaustion, we introduce three key CD8+ T cell subsets into our framework. Among these, $T_{f}$ is characterized by robust proliferative capacity and comprehensive immune effector functions. Under persistent tumor antigen stimulation, $T_{f}$ progressively differentiates into $T_{p}$ and eventually into $T_{t}$, a biological cascade coupled with a gradual loss of proliferative potential and immune function [1–5]. Based on this mechanism, the dynamics of the CD8+ T cell populations are formulated as follows:

$$\begin{array}{c} \frac{dT_{f}}{dt}=\underset{activation}{\underbrace{J_{\text{act}}^{T_{f}}(C,T_{r},\gamma_{1})}}+\underset{proliferation}{\underbrace{\alpha_{T_{f}}\frac{T_{h}}{T_{h}+E_{T_{h}}}\left(1-\frac{T_{f}+T_{p}+T_{t}}{K_{T}}\right)T_{f}}}-\underset{exhaustion}{\underbrace{J_{\text{exh}}^{T_{f}}(C,T_{f},\gamma_{2})}}-\underset{death}{\underbrace{d_{T_{f}}T_{f}}}, \end{array}$$ (2)

$$\begin{array}{cc} \frac{dT_{p}}{dt}= & \underset{proliferation}{\underbrace{\alpha_{T_{p}}\left(1-\frac{T_{f}+T_{p}+T_{t}}{K_{T}}\right)T_{p}}}+\underset{exhaustion}{\underbrace{J_{\text{exh}}^{T_{f}}(C,T_{f},\gamma_{2})-J_{\text{exh}}^{T_{p}}(C,T_{p})}}-\underset{death}{\underbrace{d_{T_{p}}T_{p}}}, \end{array}$$ (3)

$$\begin{array}{c} \frac{dT_{t}}{dt}=\underset{proliferation}{\underbrace{\alpha_{T_{t}}\left(1-\frac{T_{f}+T_{p}+T_{t}}{K_{T}}\right)T_{t}}}+\underset{exhaustion}{\underbrace{J_{\text{exh}}^{T_{p}}(C,T_{p})}}-\underset{death}{\underbrace{d_{T_{t}}T_{t}}}. \end{array}$$ (4)

In Equations (2), (3), and (4), $\alpha_{T_{f}}$, $\alpha_{T_{p}}$, and $\alpha_{T_{t}}$ denote the respective proliferation rates of $T_{f}$, $T_{p}$, and $T_{t}$, respectively; $E_{T_{h}}$ represents the half-saturation constant for $T_{h}$; $K_{T}$ is the carrying capacity for cytotoxic T cells; and $d_{T_{f}}$, $d_{T_{p}}$, and $d_{T_{t}}$ represent the clearance rates of these subsets. The kinetic terms governing these cell state transitions are defined below:

- Activation process ($T_{N8}\to T_{f}$):

$$\begin{array}{c} J_{\text{act}}^{T_{f}}(C,T_{r},\gamma_{1})=\lambda_{T_{f}}(1+\gamma_{1})\cdot \frac{C}{E_{C}+C}\cdot \frac{1}{1+T_{r}/E_{T_{f}T_{r}}}\cdot T_{N8}. \end{array}$$ (5)

Here, $\lambda_{T_{f}}$ is the baseline activation rate of $T_{f}$; $\gamma_{1}$ represents the therapeutic enhancement of T cell activation by anti-CTLA-4 blockade; $E_{C}$ and $E_{T_{f}T_{r}}$ act as the half-saturation constants for *C* and $T_{r}$, respectively; and $T_{N8}$ indicates the baseline level of naïve CD8+ T cells.

- Progenitor exhaustion process ($T_{f}\to T_{p}$):

$$\begin{array}{c} J_{\text{exh}}^{T_{f}}(C,T_{f},\gamma_{2})=\beta_{T_{p}T_{f}}(1-\gamma_{2})\cdot \frac{{\left(\int_{t-\tau_{1}}^{t}C(s)ds\right)}^{n_{1}}}{E_{pf}^{n_{1}}+{\left(\int_{t-\tau_{1}}^{t}C(s)ds\right)}^{n_{1}}}\cdot T_{f}. \end{array}$$ (6)

A time-delayed integral formulation is employed here to capture the biological memory effect of T cell exhaustion induced by persistent antigenic stimulation. The integral term $\int_{t-\tau_{1}}^{t}C(s)ds$ quantifies the cumulative antigen exposure over the time window $\tau_{1}$. A Hill function $\frac{{\left(\int_{t-\tau_{1}}^{t}C(s)ds\right)}^{n_{1}}}{E_{pf}^{n_{1}}+{\left(\int_{t-\tau_{1}}^{t}C(s)ds\right)}^{n_{1}}}$ describes the continuous quantitative mapping from cumulative stimulation to the induction of exhaustion. $E_{pf}$ is the half-saturation constant and *n*_1_ is the Hill coefficient, controlling the cooperativity and threshold dependence of this response. Additionally, $\gamma_{2}$ and $\beta_{T_{p}T_{f}}$ represent the inhibitory effect of anti-PD-1 therapy and the intrinsic exhaustion rate, respectively.

- Terminal exhaustion process ($T_{p}\to T_{t}$):

$$\begin{array}{c} J_{\text{exh}}^{T_{p}}(C,T_{p})=\beta_{T_{t}T_{p}}\cdot \frac{{\left(\int_{t-\tau_{2}}^{t}C(s)ds\right)}^{n_{2}}}{E_{tp}^{n_{2}}+{\left(\int_{t-\tau_{2}}^{t}C(s)ds\right)}^{n_{2}}}\cdot T_{p}. \end{array}$$ (7)

Here, $\beta_{T_{t}T_{p}}$, $E_{tp}$, $\tau_{2}$, and *n*_2_ represent the transition rate, half-saturation constant, antigen accumulation time window, and Hill coefficient governing terminal exhaustion, respectively.

(iii) **CD4+ T cell evolution dynamics.** As complementary drivers of the immune response, we also incorporated helper ($T_{h}$) and regulatory ($T_{r}$) cells from the CD4+ lineage into the model. Their dynamics are delineated as follows:

$$\begin{array}{c} \frac{dT_{h}}{dt}=\underset{activation}{\underbrace{\lambda_{T_{h}}\cdot \frac{C}{E_{C}+C}\cdot \frac{1}{1+T_{r}/E_{T_{h}T_{r}}}T_{N4}}}+\underset{proliferation}{\underbrace{\alpha_{T_{h}}\frac{T_{h}}{E_{T_{h}}+T_{h}}T_{h}}}-\underset{death}{\underbrace{d_{T_{h}}T_{h}}}, \end{array}$$ (8)

$$\begin{array}{c} \frac{dT_{r}}{dt}=\underset{activation}{\underbrace{\lambda_{T_{r}}\cdot \frac{C}{E_{C}+C}\cdot \frac{1}{1+T_{f}/E_{T_{r}T_{f}}}\cdot \frac{1}{1+T_{h}/E_{T_{r}T_{h}}}\cdot T_{N4}}}+\underset{proliferation}{\underbrace{\alpha_{T_{r}}T_{f}}}-\underset{death}{\underbrace{d_{T_{r}}T_{r}}}, \end{array}$$ (9)

where $\lambda_{T_{h}}$ and $\lambda_{T_{r}}$ represent the activation rates for $T_{h}$ and $T_{r}$, respectively; $E_{T_{h}T_{r}}$ denotes the inhibition constant scaling the suppressive effect of $T_{r}$ on $T_{h}$ activation; $E_{T_{r}T_{f}}$ and $E_{T_{r}T_{h}}$ denote the related constants for the inhibitory effects on $T_{r}$ activation mediated by $T_{f}$ and $T_{h}$, respectively; $T_{N4}$ is the baseline abundance of naïve CD4+ T cells; $\alpha_{T_{h}}$ and $\alpha_{T_{r}}$ correspond to the proliferation rates of $T_{h}$ and $T_{r}$ cells, respectively; and $d_{T_{h}}$ and $d_{T_{r}}$ indicate their respective natural death rates.

Details on parameter estimation and baseline values are provided in S1 Appendix.

### The BCABC method: A computational framework for virtual cohort generation

To capture the heterogeneity of tumor progression across different therapies, we developed the boundary‑constrained approximate Bayesian computation (BCABC) method [35–37]. This approach first generates a prior virtual cohort by sampling representative kinetic parameters of the TIED model from biologically plausible ranges using Latin hypercube sampling. The BCABC method then approximates the posterior distribution via a truncated Gaussian kernel:


$$\begin{array}{c} P(\theta |x_{0})\propto \int \exp\;\left(-\frac{\rho (x_{*},x_{0}{)}^{2}}{2\epsilon^{2}}\right)\cdot 𝕀(\rho \leq \rho_{0})\cdot F(x_{*}|\theta )\cdot \pi (\theta )\;dx_{*}, \end{array}$$ (10)

where θ is the parameter vector, *x*_0_ represents the observed data, $x_{*}$ denotes the model output, ϵ is the bandwidth, $\rho_{0}$ is the tolerance threshold, and 𝕀(·) is the indicator function. Conventional unweighted distance metrics assign equal importance to all data points, which often fails to accommodate tumor volume data spanning a wide range of magnitudes. Consequently, large late-stage volumes tend to dominate the fitting process while small early‑stage errors are overlooked, introducing systematic bias into parameter estimation.

To address this limitation, we adopted an adaptive weighting strategy based on data magnitude by defining the following distance function:


$$\begin{array}{c} \rho (x_{*},x_{0})=\sum_{i=1}^{n}{\left(\frac{x_{0}(t_{i})-x_{*}(t_{i}|\theta )}{E(t_{i})}\right)}^{2}, \end{array}$$ (11)

where $E(t_{i})=[x_{0}(t_{i}){]}^{\alpha }$ if $x_{0}(t_{i})\geq V_{m}$, and $E(t_{i})=V_{m}^{\alpha }$ otherwise. Following previous studies [38,39], we set α=0.84 and $V_{m}=83\;{\text{mm}}^{3}$. This piecewise design offers two key advantages. For tumor volumes exceeding $V_{m}$, the power-law scaling mitigates the dominance of large late-stage data, ensuring the model does not exclusively fit late dynamics. Conversely, for volumes below $V_{m}$, applying a constant weight prevents the inflation of experimental noise in low-amplitude early measurements, thereby avoiding overfitting.

After obtaining the candidate parameter sets, we apply a penalty function


$$\begin{array}{c} \ell (\theta )=\sum_{i=1}^{n}\max\left[{\left(x_{*}(t_{i}|\theta )-\frac{l_{i}+u_{i}}{2}\right)}^{2}-{\left(\frac{u_{i}-l_{i}}{2}\right)}^{2},\;0\right] \end{array}$$ (12)

to exclude any parameter combinations that produce trajectories falling outside the experimentally plausible bounds $\left[l_{i},u_{i}\right]$. Only those satisfying ℓ(θ)=0 alongside the final tumor volume constraints specific to each treatment group are retained to form the final cohort. Using this refined virtual cohort, we conducted a population-level dynamic analysis of tumor-immune interactions. Further algorithmic details and computational implementations are provided in S2 Appendix.

### The TIED-ABM algorithm: A spatial agent-based simulator for T cell exhaustion dynamics

**Fig 3:**
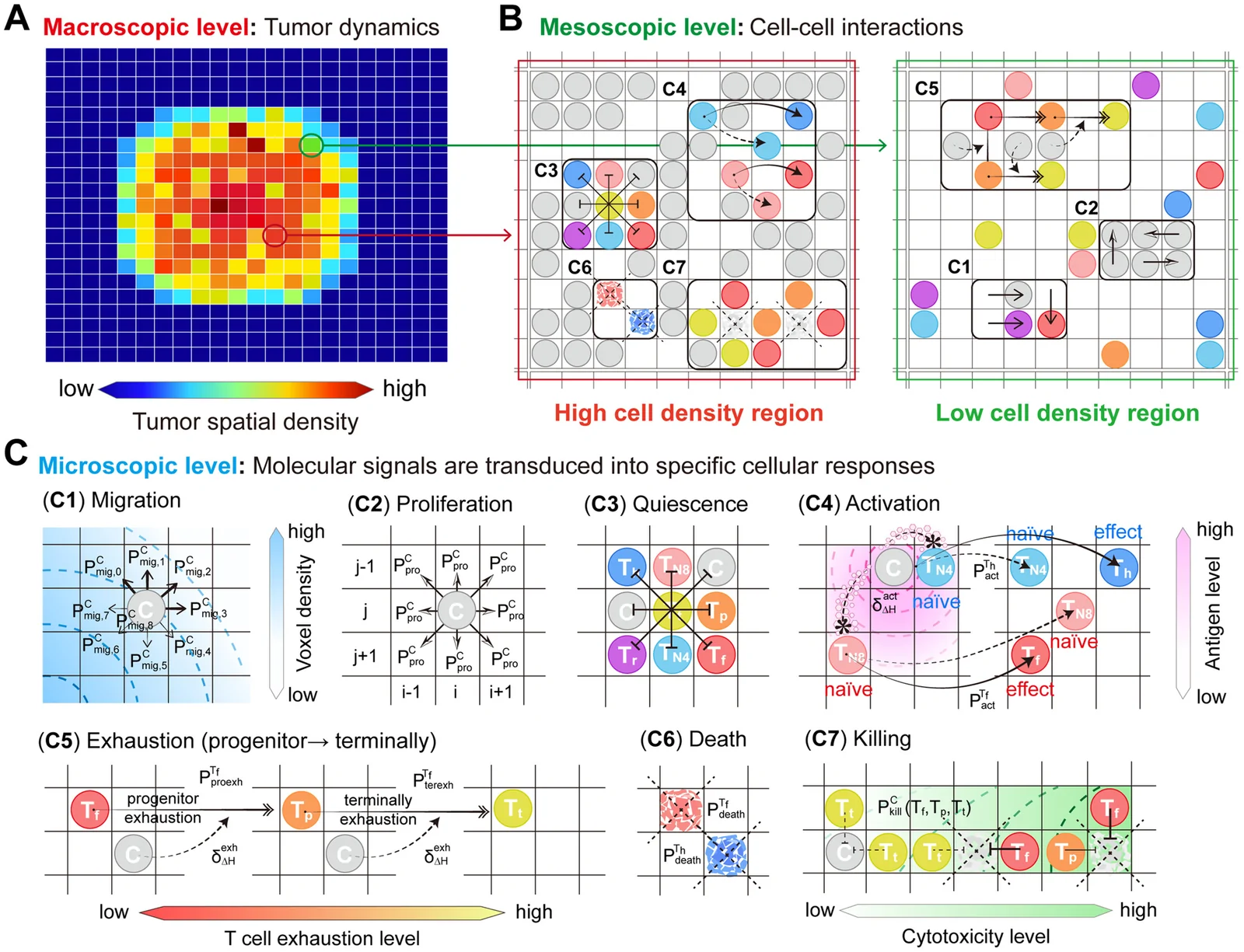
Multiscale mechanisms of tumor-immune interactions and rule design of the TIED-ABM algorithm. At the macroscopic level, population evolutionary dynamics are visualized by voxel colors representing the spatial density of the tumor, with redder colors indicating higher tumor density **(A)**. At the mesoscopic level, intercellular communications and local interactions are evaluated, where a 10×10 cellular array constitutes one voxel module **(B)**. At the microscopic level, molecular signals are transduced into specific cellular responses **(C)**. Within this multiscale framework, the TIED-ABM algorithm executes the following rules: (**C1**) migration; (**C2**) proliferation; (**C3**) quiescence; (**C4**) activation; (**C5**) exhaustion; (**C6**) death; (**C7**) killing.

To capture the spatial and stochastic dynamics of T cell exhaustion at the single-cell level, we designed a spatial agent‑based simulator, termed the TIED‑ABM algorithm. This computational framework employs a Poisson process to bridge the deterministic dynamics at the macroscopic scale with the stochastic events governing individual cellular behaviors (Fig 3). Specifically, for a given biological process with a macroscopic rate λ, the probability that the event occurs exactly *k* times within a small time interval Δt follows a Poisson distribution:


$$\begin{array}{c} \mathrm{Poisson}(k,\lambda \Delta t)=\frac{(\lambda \Delta t{)}^{k}}{k!}e^{-\lambda \Delta t},\;k=0,1,2,... \end{array}$$

This formulation preserves the expected event frequency, λΔt, while introducing necessary stochasticity, thereby accurately translating equation-based population kinetics into agent-based stochastic mechanisms. Further algorithmic details are provided in S3 Appendix.

At the macroscopic scale, we discretized the spatial domain into a two-dimensional grid of voxels (Fig 3A). Each voxel encompasses a 10×10 array of lattice points, enabling clusters of cells to occupy distinct spatial microenvironments; this framework inherently captures local spatial heterogeneity. At the mesoscopic scale, we explicitly modeled the spatial interaction behavior between individual cells within a voxel (Fig 3B). The collective cell density within a voxel translates directly to the grid color observed at the macroscopic level. At the microscopic scale, seven distinct biological behaviors—migration, proliferation, quiescence, activation, exhaustion, death, and killing—are executed by mapping local spatial information onto key regulatory variables (Fig 3C). These discrete localized rules are outlined below:

- Proliferation and migration are regulated by the relative voxel density ΔS(i,j), defined as ΔS(i,j)=n(i,j)/N∈[0,1], where *n*(*i*,*j*) is the total number of cells in voxel (*i*,*j*) and *N* acts as the local carrying capacity. This density constraint directly modulates the proliferation probability via a 1−ΔS scaling factor, effectively penalizing cell division as space becomes crowded. For migration, cells have a higher probability of leaving a densely populated voxel and preferentially navigate toward adjacent voxels with lower densities.
- Activation of naïve T cells is driven by the spatial distribution of tumor antigens. We designated a ‘tumor core’ as any voxel where the tumor cell density surpasses a critical threshold: $\Delta C=n_{C}(i,j)/N>0.5$, with $n_{C}(i,j)$ evaluating the local tumor cell count. The antigen distance ΔH(i,j) is defined as the Euclidean distance to the nearest tumor core, reflecting the spatial decay of antigen availability. Consequently, the activation probability is modulated by a Hill function:

$$\begin{array}{c} \delta_{\Delta H}^{\text{act}}=V_{\text{act}}\cdot \frac{K_{\text{act}}^{n}}{K_{\text{act}}^{n}+(\Delta H{)}^{n}}, \end{array}$$

where *V*_act_ is the maximum activation rate, *K*_act_ is the half-saturation constant, and *n* is the Hill coefficient. This dependence ensures that T cell activation peaks near the tumor core and gracefully decays at greater distances.

- Exhaustion of tumor-reactive and progenitor exhausted T cells is analogously spatially dependent. Utilizing the same antigen distance ΔH(i,j), the exhaustion probability follows a comparable Hill function:

$$\begin{array}{c} \delta_{\Delta H}^{\text{exh}}=V_{\text{exh}}\cdot \frac{K_{\text{exh}}^{n}}{K_{\text{exh}}^{n}+(\Delta H{)}^{n}}, \end{array}$$

where *V*_exh_ and *K*_exh_ denote the maximum exhaustion rate and half-saturation constant, respectively. This couples exhaustion dynamics directly to the local antigen gradient, rendering T cells more susceptible to exhaustion within antigen-rich microenvironments.

- Death of exhausted T cells is accelerated by the local tumor burden. Incorporating the aforementioned tumor density ΔC, we applied a localized death enhancement factor:

$$\begin{array}{c} \delta_{\Delta C}^{\text{dys}}=V_{\text{dys}}\cdot \frac{(\Delta C{)}^{n}}{K_{\text{dys}}^{n}+(\Delta C{)}^{n}}, \end{array}$$

where *V*_dys_ specifies the maximal enhancement for a respective exhausted subset and *K*_dys_ represents the half-saturation constant. This factor effectively increases the mortality of exhausted T cells in regions heavily populated by tumor cells, reflecting the hostility of an immunosuppressive niche.

- Killing of tumor cells relies on the local cytotoxic strength ΔE(i,j), formulated as a weighted linear combination of local T cell subsets:

$$\begin{array}{c} \Delta E(i,j)=\varepsilon_{T_{f}}n_{T_{f}}(i,j)+\varepsilon_{T_{p}}n_{T_{p}}(i,j)+\varepsilon_{T_{t}}n_{T_{t}}(i,j), \end{array}$$

where $n_{T_{f}}(i,j)$, $n_{T_{p}}(i,j)$, and $n_{T_{t}}(i,j)$ signify the local counts of tumor-reactive, progenitor exhausted, and terminally exhausted T cells in voxel (*i*,*j*). The weighting coefficients ($\varepsilon_{T_{f}}$, $\varepsilon_{T_{p}}$, $\varepsilon_{T_{t}}$) quantify the cytotoxic potency specific to each T cell subset, inherently capturing the progressive loss of effector function throughout the exhaustion trajectory. Ultimately, the killing probability is saturated by a Hill factor:


$$\begin{array}{c} \delta_{\Delta E}^{\text{kill}}=V_{\text{kill}}\cdot \frac{(\Delta E{)}^{n}}{K_{\text{kill}}^{n}+(\Delta E{)}^{n}}, \end{array}$$

where *V*_kill_ represents the maximal theoretical killing rate and *K*_kill_ acts as the half-saturation threshold. This mathematically accounts for the saturation of synergistic cytotoxic activity, even at exceedingly high localized T cell densities.

**Fig 4:**
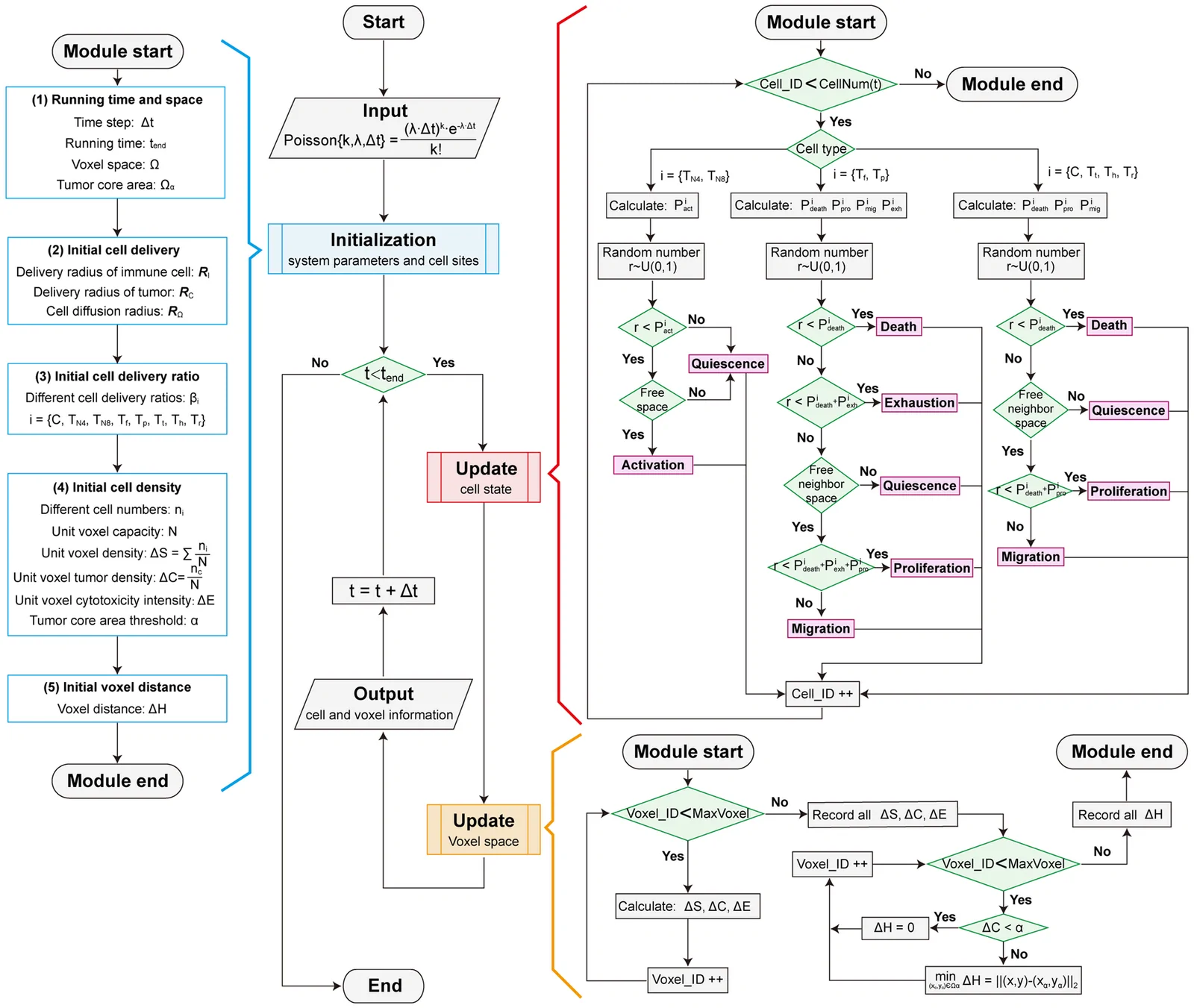
The computational workflow of the TIED-ABM algorithm.

Operationally, the TIED-ABM algorithm runs an iterative simulation over discrete time steps (Fig 4). At each iteration, the algorithm assesses every active cell within the system. For an individual cell, a uniformly distributed random variable evaluates which mutually exclusive event—migration, proliferation, quiescence, activation, exhaustion, death, or killing—will take place. These transition choices utilize probabilities strictly parameterized by the underlying Poisson processes alongside the spatial regulatory factors. Following the cell-level stochastic updates, the algorithm recalculates the macroscopic voxel variables, including the relative density ΔS(i,j), tumor core indicator ΔC, antigen distance ΔH(i,j), and cytotoxic strength ΔE(i,j) based on the subsequent spatial topology. Finally, these refreshed voxel-level variables parameterize the underlying probability maps for the subsequent time step.

## Results

### Molecular characteristics of T cell exhaustion

**Fig 5:**
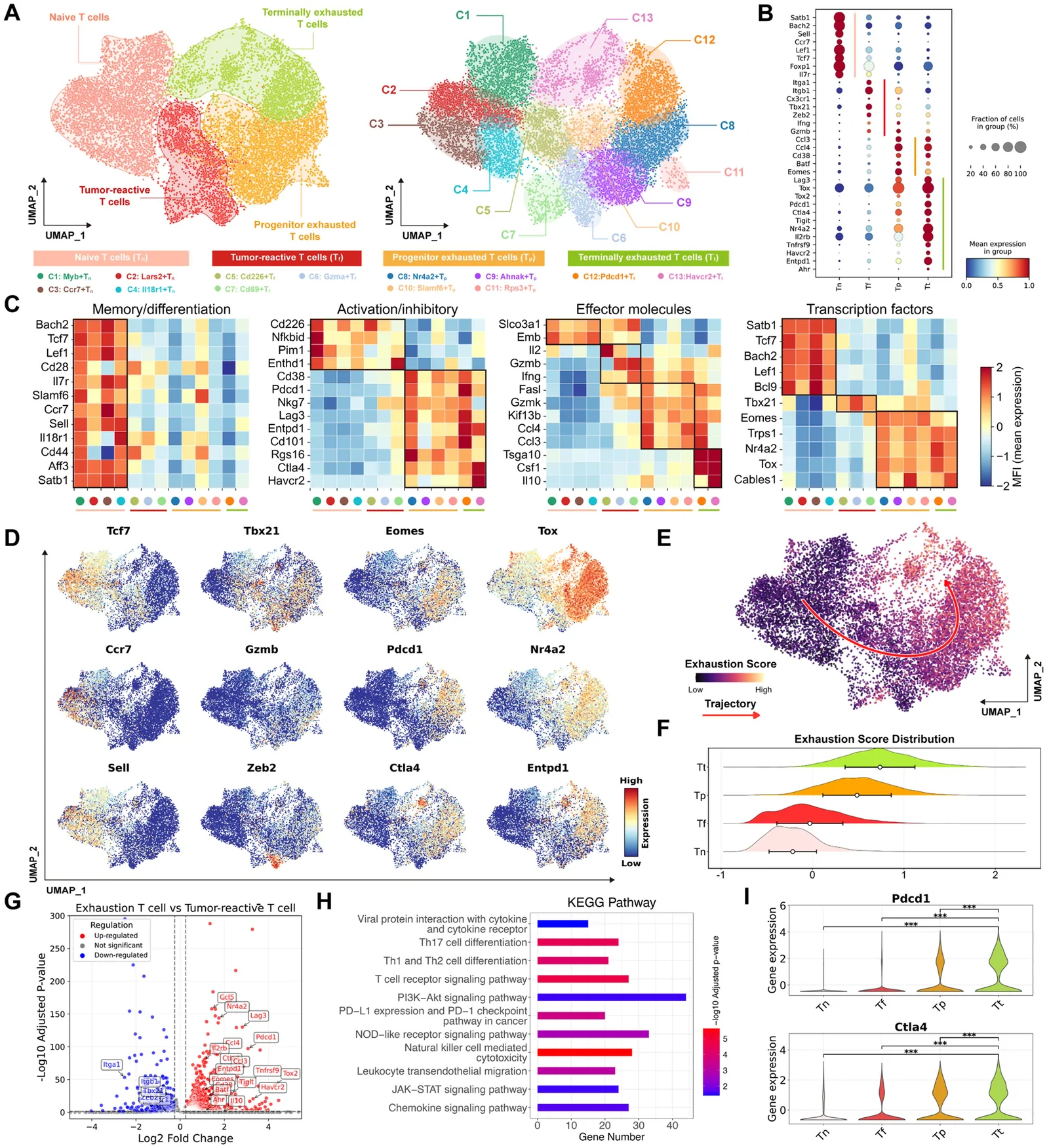
Dynamic progression and functional analysis of CD8+ T cell exhaustion at the single-cell level. **(A)** UMAP of CD8+ T cells after data cleaning and quality control. **(B)** Dot plot of marker genes for distinct T cell subsets. **(C)** Heatmaps displaying the expression of memory/differentiation, activation/inhibitory receptors, effector molecules, and transcription factors across different T cell subsets. **(D)** Feature plots showing the expression of selected genes in individual cells. **(E)**-(**F**) Exhaustion scores of T cells are evaluated using the AddModuleScore function. **(G)** Volcano plot highlighting differentially expressed genes between exhausted and tumor-reactive T cells. **(H)** KEGG enrichment analysis related to tumor immunity. **(I)** Violin plots showing the expression of *Pdcd1* and *Ctla4* in the four T cell subsets. Wilcoxon rank-sum test: *p < 0.05, **p < 0.01, ***p < 0.001.

To biologically validate the distinct CD8+ T cell phenotypes formulated in our mathematical model, we analyzed a single-cell RNA sequencing (scRNA-seq) dataset derived from murine tumor tissues (GSE262306). This dataset comprehensively profiles the transcriptional signatures associated with T cell activation and exhaustion. We performed Uniform Manifold Approximation and Projection (UMAP) dimensionality reduction to visualize cellular heterogeneity and computationally delineate unbiased cell clusters (Fig 5A). Guided by established marker genes (S4 Appendix), clusters exhibiting high expression of *Tcf7*, *Ccr7*, and *Sell* were classified as naïve CD8+ T cells ($T_{n}$) (Fig 5B and 5D). This subset represents the undifferentiated ground state of CD8+ T cells, marked by minimal expression of both effector molecules and inhibitory receptors (Fig 5C). Subsequently, clusters defined by high *Tbx21* expression, alongside elevated levels of the effector genes *Gzmb* and *Zeb2*, were annotated as functional tumor-reactive CD8+ T cells ($T_{f}$) (Fig 5B and 5D). This subset exhibits robust expression of effector molecules and lacks exhaustion-related inhibitory receptors (Fig 5C). Progenitor exhausted CD8+ T cells ($T_{p}$) were identified as clusters sharing high *Eomes* expression and moderate levels of the exhaustion marker *Havcr2* (Fig 5B and 5D). Under persistent antigenic stimulation, this subset retains partial effector activity while acquiring intermediate exhaustion features, establishing it as a reversible, transitional state (Fig 5C). Lastly, clusters characterized by elevated *Tox*, *Nr4a2*, *Pdcd1*, *Ctla4*, and *Havcr2*, coupled with diminished *Tbx21* and *Gzmb*, were defined as terminally exhausted T cells ($T_{t}$) (Fig 5B and 5D). This subset displays severely compromised effector potential alongside persistently high levels of inhibitory molecules, a signature consistent with an irreversible terminal exhaustion phenotype (Fig 5C).

To quantitatively track this phenotypic transition, we computed an exhaustion score for individual cells utilizing the AddModuleScore algorithm against a core module of exhaustion-driving genes [40,41], which includes *Tox*, *Nr4a2*, *Pdcd1*, *Havcr2*, *Tigit*, *Ctla4*, *Entpd1*, and *Il10*. This scoring maps a continuous gradient of increasing exhaustion that strictly parallels the trajectory from $T_{n}$ to $T_{f}$ and $T_{p}$, ultimately terminating at $T_{t}$ within the UMAP space (Fig 5E). Statistical comparisons of these scores confirm significant stratification among the four subsets, with the $T_{t}$ population exhibiting markedly higher exhaustion levels than the other three subsets (Fig 5F). These results biologically corroborate our mathematical assumption that CD8+ T cells progressively degenerate along a continuous axis toward terminal exhaustion.

To decouple the molecular driving forces behind this sequence, we performed differential gene expression analysis partnered with KEGG functional clustering (Fig 5G and 5H). This screening highlighted three key enriched functional pathways: (1) dysregulation of the CD4+ T cell differentiation pathway; (2) overactivation of T cell receptor signaling alongside the PD-L1/PD-1 checkpoint axis; and (3) modulation of chemokine signaling and leukocyte migration. Consistently, the critical immune checkpoint transcripts *Pdcd1* and *Ctla4* remain overexpressed across the exhaustion subsets, peaking dramatically in $T_{t}$ (Fig 5I). From a systems modeling perspective, this persistent dual-upregulation provides a strong empirical rationale for simulating combined PD-1 and CTLA-4 blockades as a targeted strategy to mechanistically rescue T cell function in subsequent analyses.

### Population dynamics analysis of T cell exhaustion

**Model calibration of population dynamics.** To rigorously evaluate the TIED model and estimate its baseline parameters, we leveraged *in vivo* time-series data of tumor growth and immune cell infiltrates from established murine experiments [42–45], providing crucial empirical support for capturing cellular-level kinetic behaviors. Tumor volume (*V*) was extrapolated from cell counts (*C*) using the conversion formula $V=\frac{V_{cell}\times C}{V_{fraction}}$, where $V_{cell}=2.572\times 10^{-6}\;{\text{mm}}^{3}/\text{cell}$ denotes the theoretical volume of a single tumor cell, and $V_{fraction}=0.37$ represents the cellular packing fraction within the tissue [20]. Although available animal experimental data capture the macroscopic trends of tumor evolution, they are insufficient to fully disentangle the complex dynamic interactions between the tumor and the immune system. Therefore, to systematically analyze these population-level dynamic characteristics, we generated a robust digital twin cohort using the BCABC method (S2 Appendix). The coefficient of determination (*R*^2^) was subsequently employed to uniformly evaluate the model’s goodness-of-fit under diverse therapeutic strategies.

**Fig 6:**
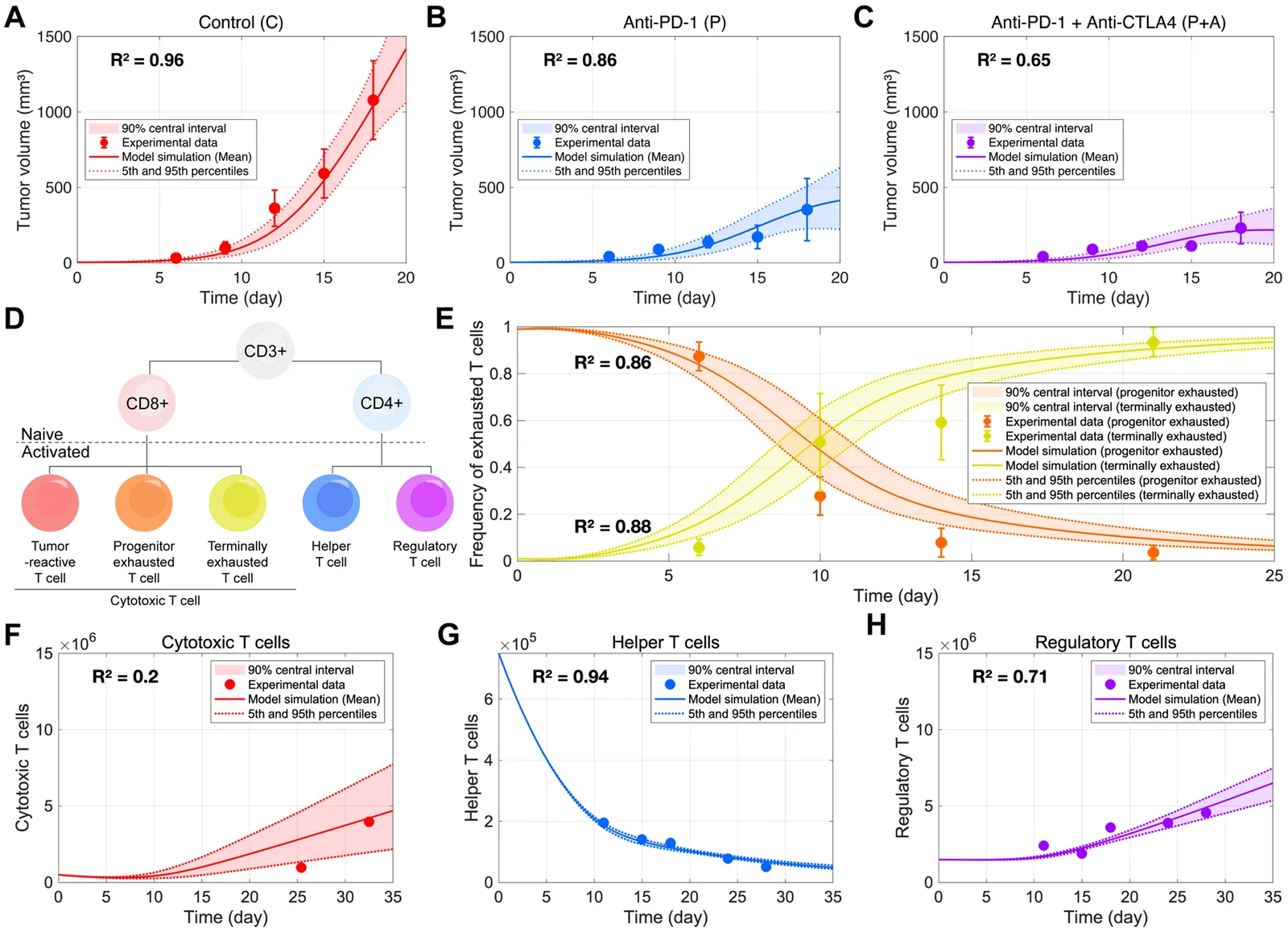
Numerical simulation and experimental calibration of cellular dynamics in the tumor-immune system. **(A)**-(**C**) Dynamic evolution of tumor volume under different treatment strategies. Solid lines represent the mean tumor dynamic trajectories of the virtual cohort. Data points and error bars indicate experimental data [42]. Shaded regions denote the 90% central intervals of tumor evolution within the virtual cohort. **(D)** Developmental lineage diagram of T cell subsets. **(E)** Longitudinal analysis of the ratio changes between progenitor exhausted T cells and terminally exhausted T cells. Experimental data are sourced from [42]. **(F)**-(**H**) Dynamic evolution of cytotoxic T cells, helper T cells, and regulatory T cells within the virtual cohort. Experimental data are derived from [43–45]. C, control; P, anti-PD-1; P + A, anti-PD-1 + anti-CTLA-4.

Trajectory assessments demonstrate strong model performance, yielding *R*^2^ values of 0.96, 0.86, and 0.65 for the control, anti-PD-1 monotherapy, and anti-PD-1 + anti-CTLA-4 combination groups, respectively (Fig 6A-6C). We further evaluated the model’s predictive capacity concerning the dynamic lineage transition of T cell subsets (Fig 6D). Longitudinal validation profiles for progenitor and terminally exhausted T cells yield high *R*^2^ values of 0.86 and 0.88, respectively (Fig 6E). For overarching CD8+ cytotoxic T cells, CD4+ helper T cells, and regulatory T cells, the corresponding *R*^2^ scores are 0.20, 0.94, and 0.71, respectively (Fig 6F-6H). The restricted goodness-of-fit observed for the macroscopic cytotoxic T cell population fundamentally stems from the limitation of having only two experimental data points available for this specific ensemble. A comprehensive sensitivity analysis of the model parameters is detailed in S5 Appendix, where we identify $\alpha_{C}$, $\alpha_{T_{r}}$, $d_{T_{h}}$, and $d_{T_{r}}$ as the primary kinetic determinants governing system behavior.

**Fig 7:**
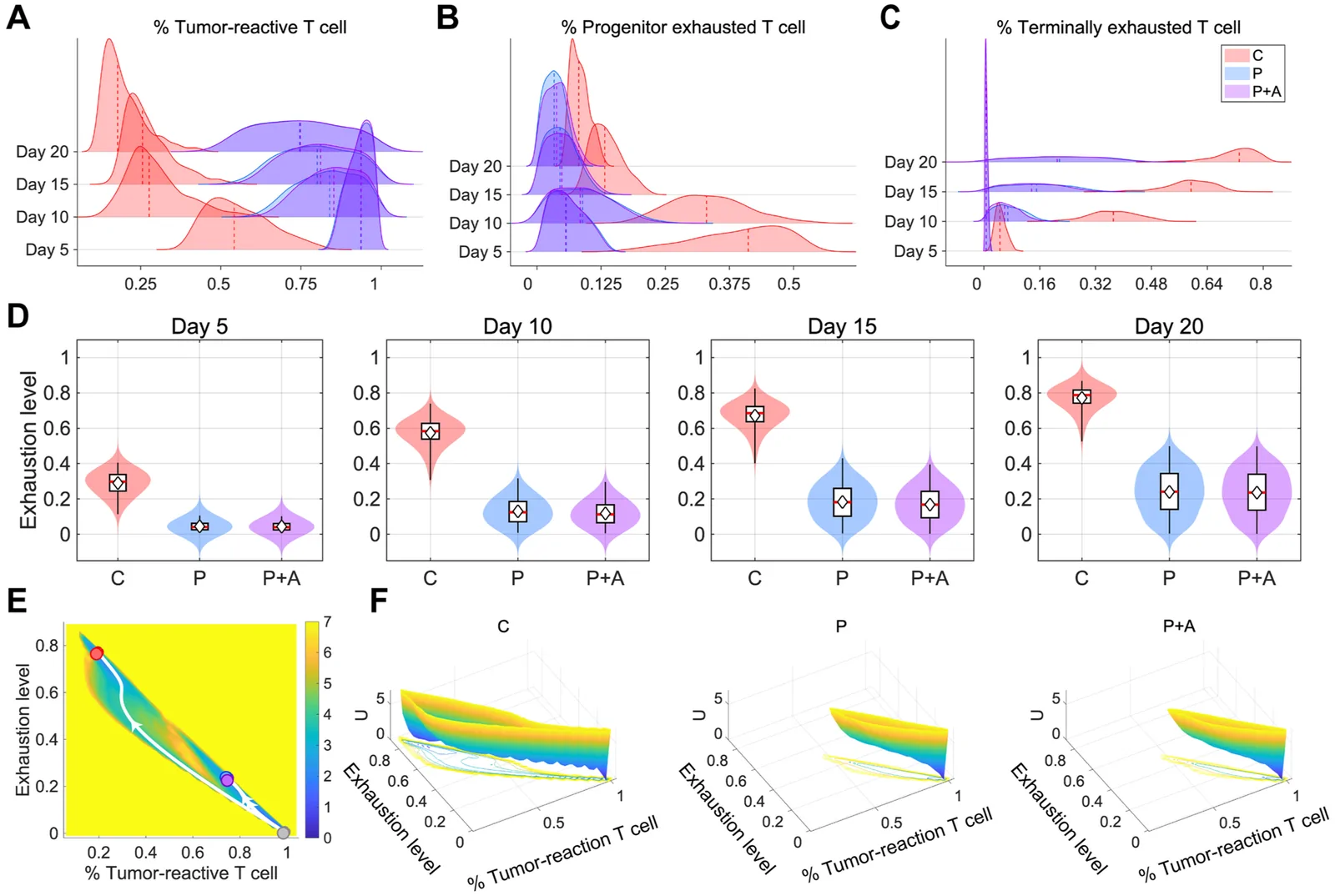
Dynamic evolution of T cell subset proportions and exhaustion levels in virtual cohorts. **(A)**-(**C**) Distribution of tumor-reactive T cells, progenitor exhausted T cells, and terminally exhausted T cells as proportions of CD8+ T cells. **(D)** Violin plots illustrating the distribution of T cell exhaustion levels. **(E)** Pseudo-potential energy constructed from T cell exhaustion levels and the percentage of tumor-reactive T cells. **(F)** Three-dimensional pseudo-potential energy. Gray dots indicate the starting points. Red, blue, and purple dots denote the endpoints for the control **(C)**, anti-PD-1 monotherapy **(P)**, and anti-PD-1 + anti-CTLA-4 combination therapy (P + A) groups, respectively. White curves represent the system’s evolutionary trajectories.

**Quantitative description of exhaustion level.** To dissect the continuous progression of CD8+ T cell exhaustion at the population level, we tracked the proportional dynamics of distinct T cell subsets on days 5, 10, 15, and 20. Our simulations indicate that immune checkpoint blockades successfully expand the pool of tumor-reactive CD8+ T cells while continuously depressing the fractions of both progenitor and terminally exhausted T cells (Fig 7A-7C). Because the functional competence of CD8+ T cells constitutes a critical driver of overall tumor dynamics, we established a quantitative composite indicator to continuously assess global exhaustion levels:


$$\begin{array}{c} Q(t)=\delta \cdot x(t)=\sum_{i=1}^{3}\delta_{i}\cdot x_{i}(t). \end{array}$$ (13)

Here, the state vector $x(t)=(x_{1}(t),x_{2}(t),x_{3}(t){)}^{⊤}$, subject to $x_{i}(t)\in [0,1]$, defines the instantaneous proportions of tumor-reactive T cells (*i* = 1), progenitor exhausted T cells (*i* = 2), and terminally exhausted T cells (*i* = 3). Correspondingly, $\delta =(\delta_{1},\delta_{2},\delta_{3})$ acts as the weight vector bridging subset proportions to systemic exhaustion severity; a larger weight signifies a mechanically more dysfunctional cell state. Guided by the empirical exhaustion scores derived from our earlier scRNA-seq analysis (Fig 5F), we calibrated these weights using min-max normalization: $\delta_{i}=\frac{s_{i}-s_{\min}}{s_{\max}-s_{\min}}$. This formulation yields precise weight parameters of $\delta_{1}=0$, $\delta_{2}=0.6$, and $\delta_{3}=1$, where $s_{i}$ represents the raw average exhaustion score for cell type *i*, while $s_{\min}$ and $s_{\max}$ enclose the minimum and maximum scores across all T cell classes examined. Specifically, enforcing $\delta_{1}=0$ inherently anchors the fully functional tumor-reactive T cell subset as the exhaustion‑free baseline for this continuous macroscopic quantification.

Temporal profiling of this metric demonstrates that during the nascent stages of tumor evolution, systemic T cell exhaustion remains marginal (Fig 7D). However, as local tumor burden continually accumulates, T cells are inexorably driven toward terminal exhaustion, manifested by a steady ascent in the cumulative exhaustion score (Fig 7D). Crucially, these macroscopic dynamics align perfectly with our microscopic single-cell sequencing findings, reaffirming that exhausted subsets persistently dominate the overall systemic dysfunction profile (Fig 5E and 5F). As theoretically expected, therapeutic intervention via immune checkpoint blockade markedly attenuates the acceleration of this systemic exhaustion (Fig 7D).

**Fig 8:**
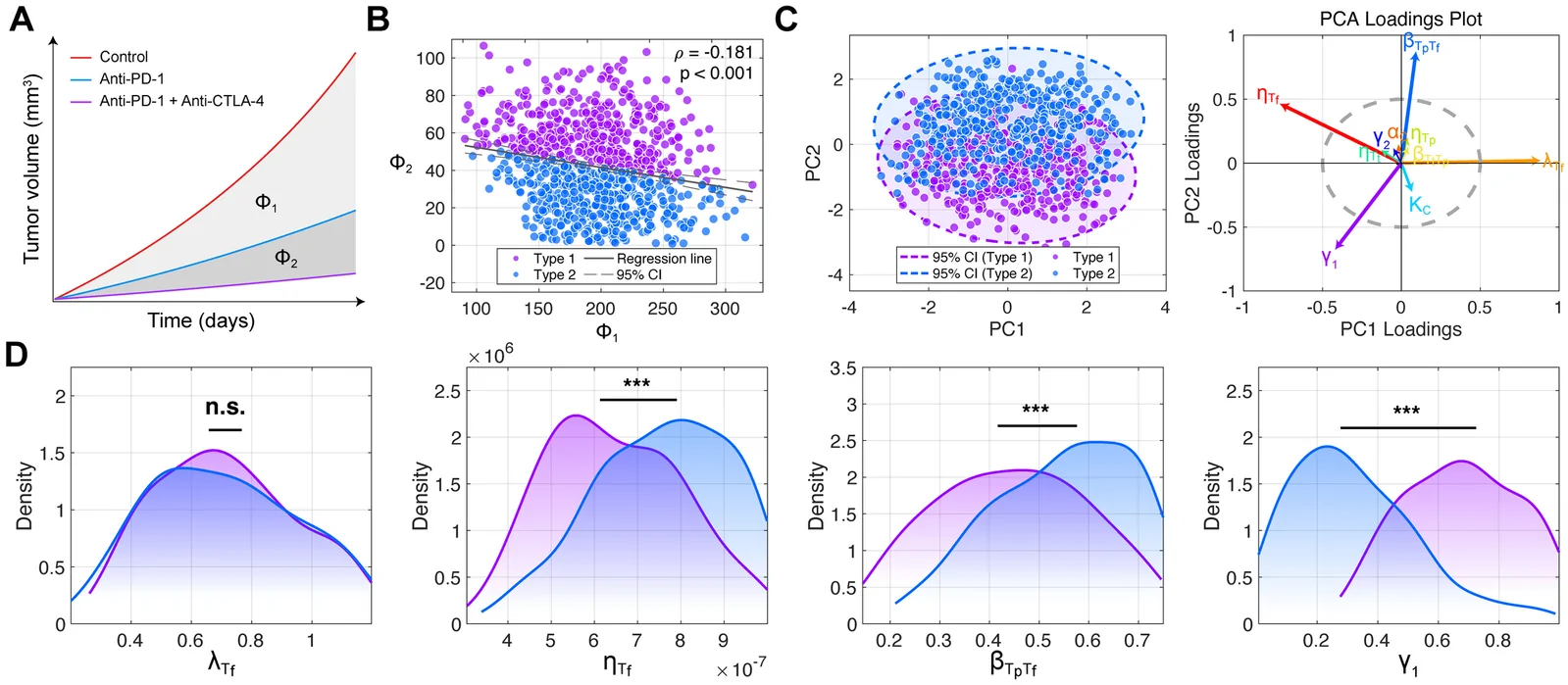
Elucidating the heterogeneity and driving mechanisms of combination immunotherapy efficacy. **(A)** Quantitative indicators for immunotherapy-induced tumor burden reduction. **(B)** Scatter plot based on $\Phi_{1}$ and $\Phi_{2}$ stratifying treatment responses into combination high-benefit (purple dots) and low-benefit (blue dots) populations. **(C)** Principal component analysis (PCA) was employed to quantify the clustering structure of treatment response differences within a multi-parameter space. PC1: Principal component 1. PC2: Principal component 2. **(D)** Distribution of key parameters in the benefits of the two treatments. The Wilcoxon rank-sum test was used to assess significant differences between distributions. *p < 0.05, **p < 0.01, ***p < 0.001.

**Efficacy heterogeneity and mechanistic analysis.** To systematically quantify the inter-individual variability inherent to immune checkpoint blockade responses, we constructed two integral-based metric indicators, $\Phi_{1}$ and $\Phi_{2}$ (Fig 8A). $\Phi_{1}$ captures the time-averaged reduction in tumor burden achieved exclusively by anti-PD-1 monotherapy relative to baseline unhindered growth:


$$\begin{array}{c} \Phi_{1}=\frac{1}{T}\int_{0}^{T}(V_{1}(t)-V_{2}(t))dt, \end{array}$$ (14)

where *V*_1_(*t*) and *V*_2_(*t*) denote the continuous tumor volumes traversing the control and anti-PD-1 monotherapy groups at time *t*, over a fixed simulation horizon *T* = 20 days. Analogously, $\Phi_{2}$ isolates the supplementary therapeutic benefit dynamically conferred by the addition of anti-CTLA-4 to the baseline anti-PD-1 regimen:


$$\begin{array}{c} \Phi_{2}=\frac{1}{T}\int_{0}^{T}(V_{2}(t)-V_{3}(t))dt, \end{array}$$ (15)

wherein *V*_3_(*t*) characterizes the evolving tumor volume under dual-blockade conditions.

Interestingly, population-level correlation analysis reveals a mild negative tradeoff between monotherapy response and combination upside (Pearson’s ρ=−0.181). This pattern implies that patients exhibiting substantial volumetric control via monotherapy may experience mathematically diminishing returns upon the introduction of a secondary checkpoint blockade (Fig 8B). To isolate the mechanistic drivers of this treatment divergence, principal component analysis (PCA) was performed across the parameter space, successfully uncoupling four pivotal kinetic determinants: the activation rate of tumor-reactive T cells ($\lambda_{T_{f}}$), intrinsic tumor killing rate ($\eta_{T_{f}}$), progenitor exhaustion propensity ($\beta_{T_{p}T_{f}}$), and the pharmacodynamic efficacy coefficient of anti-CTLA-4 ($\gamma_{1}$) (Fig 8C). Subsequent nonparametric comparative statistics confirm that while the structural distributions of $\eta_{T_{f}}$, $\beta_{T_{p}T_{f}}$, and $\gamma_{1}$ vary significantly between clinical response strata, intrinsic activation ($\lambda_{T_{f}}$) variations remain statistically unremarkable in isolation (Fig 8D). Strikingly, subsets of the virtual cohort selectively maximized their combination-therapy benefits specifically under conditions mapping to heightened $\gamma_{1}$ efficiency coupled with inherently subdued $\eta_{T_{f}}$ and $\beta_{T_{p}T_{f}}$ constraints (Fig 8D). Ultimately, this multi-parameter stratification confirms that optimal therapeutic combinations do not act independently but instead strictly hinge upon the synchronized interplay between T cell recruitment, exhaustion resistance, and cytotoxic execution.

### Response pattern of T cell exhaustion

**Quantitative mapping of activation-exhaustion space.** To systematically evaluate the coupled impact of the naïve T cell activation rate ($\lambda_{T_{f}}$) and the progenitor exhaustion rate ($\beta_{T_{p}T_{f}}$) on system-level tumor-immune dynamics, we formulated a normalized integral metric:


$$\begin{array}{c} E_{x}(\lambda_{T_{f}},\beta_{T_{p}T_{f}})=\frac{1}{n}\sum_{i=1}^{n}\int_{0}^{T}\frac{x_{i}(t;\lambda_{T_{f}},\beta_{T_{p}T_{f}})-x_{i}^{*}(t)}{x_{i}^{*}(t)}dt. \end{array}$$ (16)

**Fig 9:**
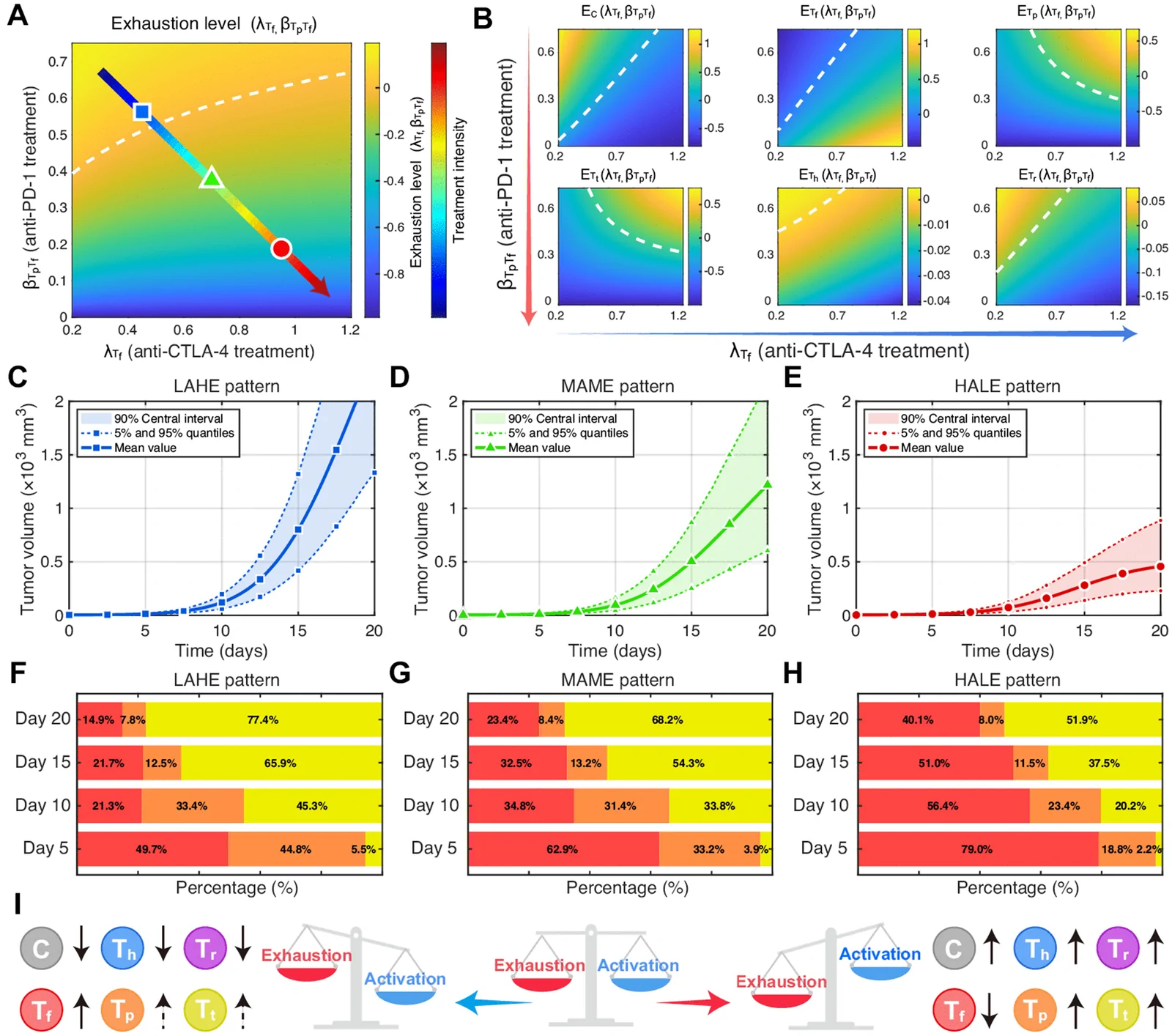
Impact of T cell activation rate ($\lambda_{T_{f}}$) and progenitor exhaustion rate ($\beta_{T_{p}T_{f}}$) on tumor-immune dynamics. **(A)**-(**B**) Two-dimensional heatmaps depicting relative changes in systemic exhaustion levels, tumor volume, and immune subset frequencies across continuous parameter landscapes ($\lambda_{T_{f}},\beta_{T_{p}T_{f}}$). White iso-curves demarcate parameter combinations ensuring equivalent dynamical outcomes relative to the baseline. Blue squares, green triangles, and red circles pinpoint coordinate configurations corresponding to the low activation/high exhaustion (LAHE), moderate activation/moderate exhaustion (MAME), and high activation/low exhaustion (HALE) regimes, respectively. **(C)**-(**E**) Macroscopic tumor evolution dynamics simulated out within the LAHE, MAME, and HALE domains. Solid lines delineate the mean tumor growth trajectories of the cohort, with shaded regions capturing the 90% central interval. **(F)**-(**H**) Mean proportional transitions of tumor-reactive, progenitor exhausted, and terminally exhausted T cells beneath the LAHE, MAME, and HALE regimens. **(I)** Schematic mapping the biological role of the activation-exhaustion balance upon overarching tumor-immune outcomes.

Here, the state variable block *x* belongs to the set $\left\{Q,C,T_{f},T_{p},T_{t},T_{h},T_{r}\right\}$. The term $x_{i}(t;\lambda_{T_{f}},\beta_{T_{p}T_{f}})$ defines the continuous temporal trajectory of variable *x* for the *i*-th virtual patient traversing the perturbed parameter subspace $\left(\lambda_{T_{f}},\beta_{T_{p}T_{f}}\right)$, whereas $x_{i}^{*}(t)$ represents the intrinsic reference trajectory for the identical digital twin simulated under baseline physiological parameter values. Furthermore, *n* specifies the total size of the virtual cohort, and the integration horizon *T* = 20 days establishes the computational evaluation period. Viewed through a pharmacodynamic lens, mechanically elevating $\lambda_{T_{f}}$ mimics an amplified anti-CTLA-4 blockade, thereby stimulating naïve T cell priming and activation (Fig 9A). Conversely, functionally downregulating $\beta_{T_{p}T_{f}}$ acts as a proxy for an effective anti-PD-1 intervention, continuously rescuing effector cells from progressive exhaustion (Fig 9A).

Perturbation analysis across this two-dimensional parameter space reveals that simultaneously dampening $\beta_{T_{p}T_{f}}$ and augmenting $\lambda_{T_{f}}$ drives a monotonic topological decline in both global exhaustion levels and cumulative tumor burden (Fig 9A and 9B). This specific kinetic tuning preferentially expands the tumor-reactive T cell pool while contracting the immunosuppressive helper and regulatory compartments (Fig 9B). Mechanistically, this demonstrates that coordinating exhaustion reversal with enhanced activation actively reprograms the local suppressive microenvironment rather than merely augmenting innate cytotoxicity. Strikingly, escalating both $\beta_{T_{p}T_{f}}$ and $\lambda_{T_{f}}$ cooperatively triggers a paradoxical surge in progenitor and terminally exhausted phenotypes (Fig 9B). This non-linear dynamic implies that aggressively pushing T cell activation without simultaneously applying an exhaustion safeguard inadvertently forces functional effectors down a terminal differentiation sink, ultimately collapsing antitumor immunity. Thus, optimal therapeutic efficacy stringently relies upon mathematically balancing the regenerative influx (activation) against the degenerative efflux (exhaustion) fluxes modulating the effector subset.

To definitively decouple these divergent microenvironmental states, we partitioned the parameter space into three hallmark activation-exhaustion dynamical regimes guided by specific kinetic combinations:

- LAHE pattern (low activation and high exhaustion): $\left(\lambda_{T_{f}},\beta_{T_{p}T_{f}})=(0.45,0.5625\right)$, encoding a profoundly immunosuppressive state.
- MAME pattern (moderate activation and moderate exhaustion): $\left(\lambda_{T_{f}},\beta_{T_{p}T_{f}})=(0.70,0.3750\right)$, establishing a precarious dynamical equilibrium.
- HALE pattern (high activation and low exhaustion): $\left(\lambda_{T_{f}},\beta_{T_{p}T_{f}})=(0.95,0.1875\right)$, driving a robust and durable antitumor state.

Simulations operating along the LAHE trajectory display exponential tumor escape coupled with a catastrophic accumulation of terminally exhausted T cells (Fig 9C and 9F). Dynamically, this confirms that the LAHE regime accelerates the assembly of an exhausted niche, rendering tumor evasion inevitable. Conversely, the MAME trajectory imposes a deceleration in tumor outgrowth (Fig 9D). In direct contrast to the LAHE baseline, the terminal exhaustion fraction under this moderate constraint gracefully falls from 77.4% down to 68.2% by day 20 (Fig 9G). Crucially, therapeutic emulation via the HALE regimen effectively induces profound tumor collapse (Fig 9E). Operating within this optimized parameter basin remarkably preserves functional effector integrity, sustaining the macroscopic proportions of tumor-reactive T cells at peak values of 79.0%, 56.4%, 51.0%, and 40.1% upon days 5, 10, 15, and 20, respectively (Fig 9H).

**Mechanistic rationalization of combination therapies.** These multi-scale numerical analyses emphatically underscore that the competing rate configurations steering CD8+ T cell priming and exhaustion unilaterally dictate the bifurcation between successful tumor clearance and unconstrained immune escape (Fig 9I). Geometrically navigating the modeled system toward a high-activation, low-exhaustion parameter basin constitutes the most biologically reliable methodology for engineering durable antitumor immunity. Unbridled molecular activation bereft of corresponding exhaustion suppression merely hastens the depletion of functional T cells. Consequently, safely guiding the immune response towards a stable tumor-clearing equilibrium necessitates a mathematically dualistic approach: chemically potentiating systemic influx signals via CTLA-4 blockade while strictly bottlenecking localized exhaustion propagation via anti-PD-1 therapies. Ultimately, this dynamical systems framework engineers a resilient theoretical substrate for rationally designing advanced synergistic immunotherapy strategies.

### Spatiotemporal multiscale modeling of T cell exhaustion

**Fig 10:**
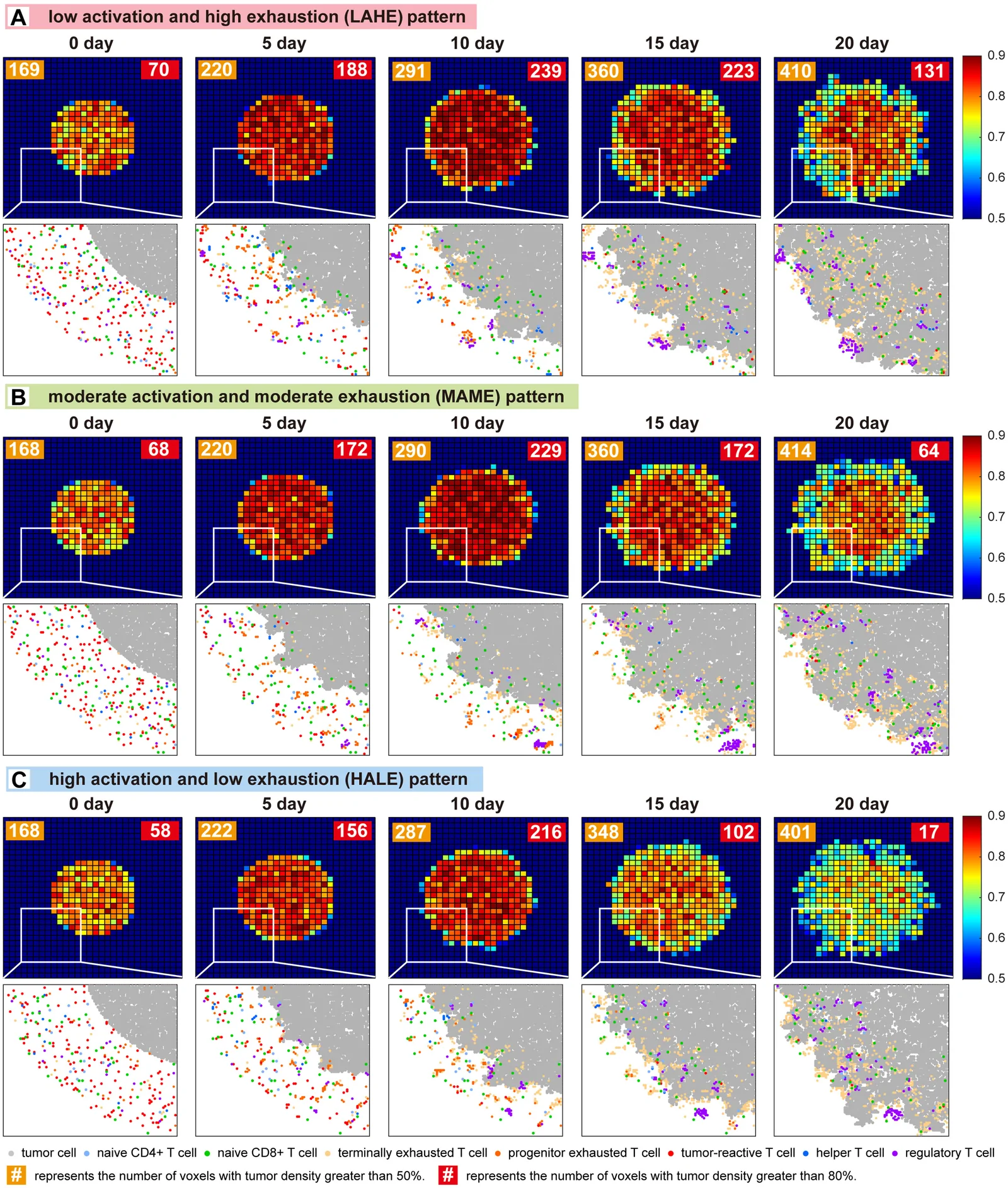
Two-dimensional simulation of tumor dynamics and T cell exhaustion under three activation-exhaustion patterns. **(A)** LAHE pattern. **(B)** MAME pattern. **(C)** HALE pattern. At the tissue level, distinct color blocks indicate variations in tumor density within each voxel. At the cellular level, gray denotes tumor cells; light blue, naïve CD4+ T cells; green, naïve CD8+ T cells; light yellow, terminally exhausted T cells; orange, progenitor exhausted T cells; red, tumor-reactive T cells; blue, helper T cells; and purple, regulatory T cells. Numbers in the orange rectangle delineate voxels with tumor density exceeding 50%, with red denoting those above 80%.

**Spatiotemporal evolution of the solid tumor microenvironment.** Using the TIED-ABM algorithm, we reconstructed the spatiotemporal evolutionary dynamics of tumors and T cell exhaustion across the three characteristic activation-exhaustion phase spaces (S1 Video). To explicitly resolve the biophysical dynamics across these distinct microenvironments, we extracted spatial snapshots spanning days 0, 5, 10, 15, and 20 (Fig 10). This discrete periodic sampling permitted continuous tracking of voxel-based structural evolution at the macroscopic tissue level, while simultaneously monitoring the agent-based kinetic interactions between malignant and immune lineages at the cellular scale. At the tissue scale, computational simulations reveal that the number of spatial voxels with tumor occupancy (ΔC) exceeding 50% escalates from 169 to 410 under the LAHE configuration (Fig 10A). Similarly, under the MAME and HALE patterns, these intermediate-density subregions expand from 168 to 414 and from 168 to 401, respectively (Fig 10B and 10C). Further spatial quantification of critical domains (ΔC>80%) demonstrates that the LAHE trajectory dramatically exacerbates the localized consolidation of high-density tumor nodules. By day 20, the absolute number of these critical voxels (ΔC>80%) under the HALE, MAME, and LAHE regimens stands at 17, 64, and 131, respectively (Fig 10). These topological mappings quantitatively confirm that the HALE configuration, defined by potent naïve activation and stringent exhaustion suppression, is the most pharmacodynamically effective mechanism for structurally constraining localized tumor burden. Conversely, the LAHE macro-state explicitly licenses the unhindered expansion of densely packed malignant domains.

**Fig 11:**
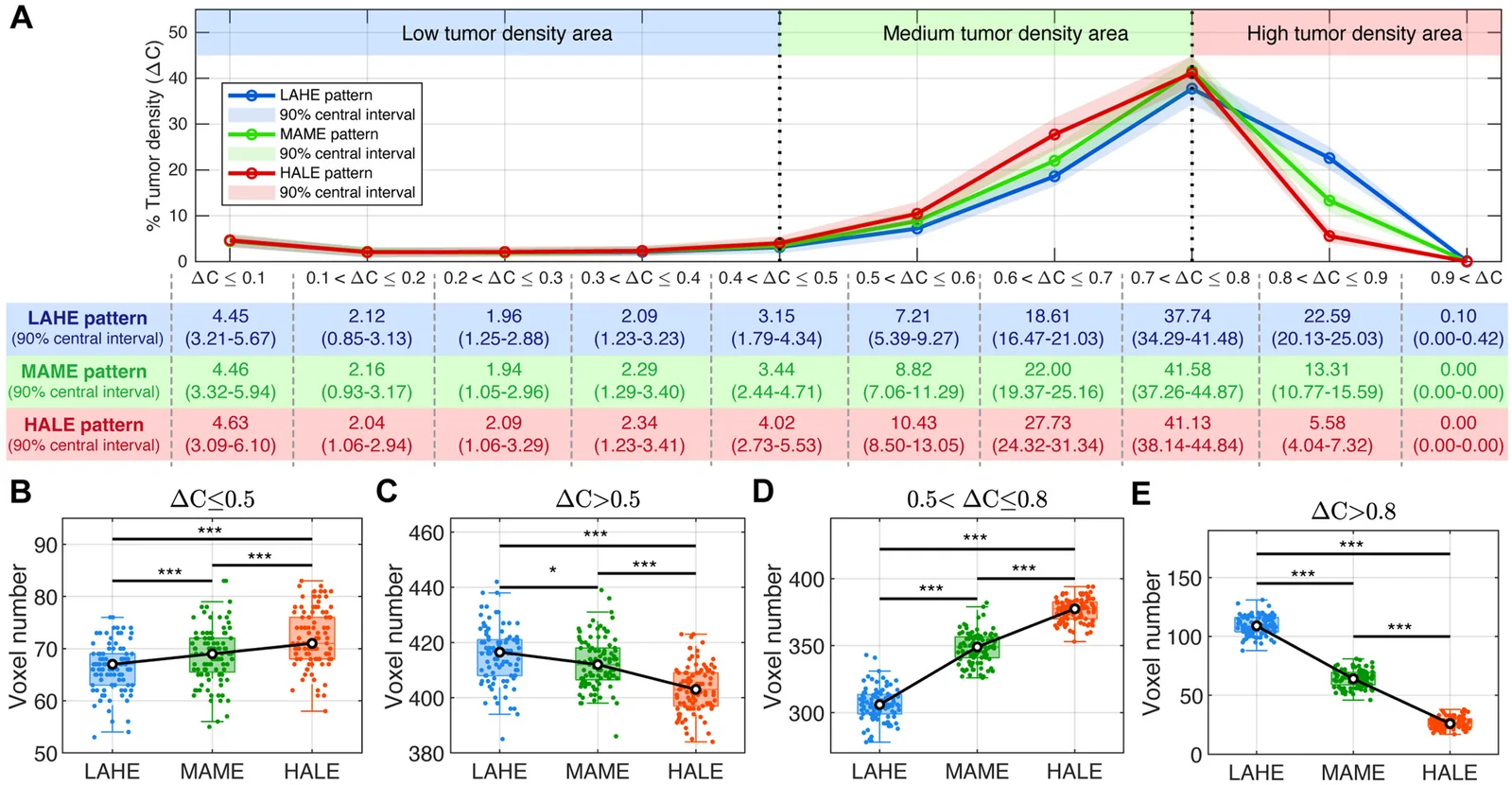
Statistical analysis of tumor voxel density (ΔC) under three activation-exhaustion patterns. **(A)** Distribution of ΔC across different intervals at the end of simulation. **(B)** Boxplots of tumor voxel density distributions under the conditions of ΔC≤0.5, ΔC>0.5, 0.5<ΔC≤0.8, and ΔC>0.8. The Wilcoxon rank-sum test was used to assess significant differences between distributions. *p < 0.05, **p < 0.01, ***p < 0.001.

**Statistical robustness of spatial heterogeneity.** To guarantee the statistical robustness of these spatial structures and account for intrinsic cellular stochasticity, we executed 100 independent Monte Carlo realizations (Fig 11A). Ensemble averaging demonstrates that across all three dynamic regimes, the foundational fraction of low-density voxels (ΔC≤0.5) persistently hovers below 6% (Fig 11A). Under the optimal HALE intervention, spatial progression is remarkably arrested, with the spatial majority confined within the intermediate density phase (0.5<ΔC≤0.8) (Fig 11A). By sharp contrast, mapping the LAHE trajectory reveals a catastrophic topological shift, where the majority of spatial domains coalesce into critically dense physical clusters (0.8≤ΔC) (Fig 11A). Notably, within the LAHE microenvironment, 22.59% of the spatial domain is driven into the severe 0.8<ΔC≤0.9 density bracket, contrasting sharply with merely 13.31% and 5.58% observed under the MAME and HALE regimens, respectively (Fig 11A). These granular spatial statistics reinforce the physical reality that attenuated activation signaling naturally coupled with heightened exhaustion synergistically engineers a localized architecture characterized by mechanically impenetrable tumor densities.

**Comparative topology of therapeutic modes.** Subsequent non-parametric validations expose profound stratification in the emergent tumor density distribution (ΔC) uniquely dictated by the governing activation-exhaustion balance (Fig 11B-11E). Relative to the immunosuppressive LAHE and transitional MAME trajectories, the HALE framework retains a functionally superior frequency of sparse-density spatial bins (ΔC≤0.5) while concurrently minimizing the emergence of structurally dense cellular pockets (ΔC>0.5) (Fig 11B and 11C). Operating within the HALE regime efficiently restricts the structural majority of voxels to the intermediate density range (0.5<ΔC≤0.8), maintaining a spatial distribution significantly elevated compared to the LAHE and MAME conditions (Fig 11D). Symmetrically, the terminal probability of countering extreme macroscopic densities (ΔC>0.8) is heavily restricted under the HALE regime relative to alternate configurations (Fig 11E). Ultimately, this multiscale architecture mechanistically substantiates that driving systemic immune parameters into the HALE phase basin reliably bottlenecks malignant growth at physically manageable intermediate densities, explicitly circumventing the densely packed, highly immunosuppressive solid voids symptomatic of the LAHE state.

## Discussion

T cell exhaustion represents a fundamentally distinct dysfunctional state driven by chronic antigenic stimulation [1,2]. Rather than a simple functional decline, it is characterized by an organized hierarchy of impaired effector functions, sustained inhibitory receptor expression, and profound transcriptional and epigenetic reprogramming [1–4]. This terminal differentiation compromises the immune system’s capacity for tumor clearance, thereby accelerating malignant immune evasion [2,3]. While clinical interventions—predominantly checkpoint blockades targeting PD-1, PD-L1, and CTLA-4—have achieved considerable success in partially reversing this exhausted state [12,14], a rigorous mathematical foundation capable of dynamically mapping how synergistic immunotherapies modulate these complex exhaustion trajectories remains largely absent.

To bridge this gap, we constructed a tumor‑immune exhaustion dynamics (TIED) model that captures the systemic co-evolution of T cell exhaustion and localized tumor growth. Rather than relying on singular deterministic outputs, we mathematically navigated inter-individual variability and tumor heterogeneity via a Boundary-Constrained Approximate Bayesian Computation (BCABC) approach. This data-driven framework allowed us to sample a probabilistic virtual cohort tightly bounded by empirical observations, thereby extracting stochastic tumor-immune dynamics at the macroscopic population level. Building upon this continuum framework, we subsequently developed the TIED-ABM algorithm. By strategically employing spatial Poisson processes, this architecture elegantly bridges deterministic ordinary differential equations with the stochastic mesoscopic dynamics of individual single-cell agents, successfully unifying disparate spatiotemporal scales.

Mechanistically, our computational integrations demonstrate that checkpoint blockade dynamically decelerates the exhaustion cascade, functionally rescuing a robust pool of tumor-reactive T cells capable of sustained cytotoxicity (Fig 7). Notably, the model quantitatively captures the nonlinear clinical reality that patients intrinsically responsive to anti-PD-1 monotherapy derive diminishing marginal returns from additional anti-CTLA-4 interventions (Fig 8). By projecting these kinetics into three distinct activation-exhaustion phase spaces, we established that the high activation and low exhaustion (HALE) dynamical regimen serves as the optimal biological basin for durable tumor containment (Fig 9). Conversely, driving the system into a high activation coupled with high exhaustion state paradoxically collapses the effector pool, facilitating unconstrained tumor escape. Furthermore, localized spatial simulations mathematically explicitly illustrate how the severe immunosuppressive microenvironment inherent to the low activation and high exhaustion (LAHE) trajectory licenses the formation of critical, high-density malignant nodules (Fig 10). Conversely, successfully navigating the immune landscape toward the HALE regime structurally bottlenecks malignant expansion, rigidly confining the localized tumor burden to physically manageable intermediate densities (Fig 11).

In formulating the underlying equations, we initially abstracted T cell exhaustion as a unidirectional, irreversible differentiation cascade. Consequently, the pharmacodynamic effect of anti-PD-1 was mathematically parameterized as a downregulation of the forward exhaustion transition rate. While emerging evidence suggests that therapeutic blockade can partially reverse exhaustion [12,14], we retained this kinetic simplification for two fundamental analytical reasons. First, from a macroscopic population dynamics viewpoint, downregulating a forward flux (exhaustion) and explicitly introducing a reverse flux both mathematically converge upon a net reduction in the progenitor exhausted pool, generating highly indistinguishable system-level continuous trajectories. Second, the transient proportions and precise kinetic timescales governing in vivo exhaustion reversal lack rigorous chronological quantification, rendering bidirectional parameters structurally unidentifiable under current data limits. Thus, representing anti-PD-1 solely as a rate attenuation provides a parsimonious yet robust mathematical approximation. As precision single-cell fate mapping techniques mature, explicit integration of stochastic reversible transitions will iteratively refine this kinetic topology. Furthermore, dynamic perturbation of exhaustion time delays structurally confirmed that prolonged exhaustion delays intrinsically favor tumor propagation and systematically deplete the tumor-reactive T cell phase (S7 Appendix). By further elevating cumulative antigen exposure to an independent state variable, comparative numerical analyses validated that defining exhaustion via a distributed delay kernel historically delivers superior mathematical fidelity in reproducing experimentally observed immune subset proportions (S8 Appendix).

Within the current TIED architecture, localized cytokine fluctuations are phenomenologically simplified as implicit directional regulatory couplings rather than explicit molecular gradients, which inherently restricts the resolution of micro-environmental signaling events. Extending the model structure to incorporate spatio-temporal cytokine distributions represents a natural pathway for enhancing biological realism. Given the finite scale of accessible in vivo temporal data, we refrained from partitioning an explicitly independent test set for macroscopic tumor volume predictions. Nevertheless, the derived transcriptomic exhaustion gradients were rigorously cross-validated against external single-cell RNA datasets, confirming the robustness of our quantitative exhaustion metrics independent of training limitations. The continuous accumulation of longitudinal multi-omic clinical datasets will critically govern further systemic validation of these theoretical projections. Importantly, synthesizing heterogeneous data modalities—spanning discrete animal models and public cross-condition single-cell repositories—introduces systemic biases strictly rooted in mismatched spatiotemporal sampling resolutions. Navigating this inherent cross-source heterogeneity remains a paramount challenge in quantitative biology. Continual standardization across experimental immunology, merged with advanced hierarchical Bayesian calibration networks, will systematically suppress these biases and enhance the generalizability of fused computational frameworks.

Recognizing the sparse temporal sampling frequency and aggregate dimensionality of the primary datasets, we deliberately deferred a fully exhaustive global identifiability analysis for the model’s broad parameter space. Intrinsic stoichiometric correlations coupled with limited degrees of statistical freedom inevitably manifest as significant uncertainty architectures during point estimation. To proactively navigate this constraint, we exclusively extracted parameter subsets via BCABC posterior sampling, subsequently interrogating overarching system sensitivities globally across the virtual meta-population. This probabilistic constraint naturally guards against overfitting by demanding structural fidelity across a bounded topology rather than isolated optima. Looking ahead, assessing the absolute integrity of the TIED network via advanced analytical protocols—such as structural profile likelihoods, rigorous Fisher information matrix diagnostics, and local coordinate identifiability frameworks [46–50]—will systematically map the uncertainty boundaries, aggressively elevating the robustness of in silico translational modeling.

Ultimately, translating the theoretical spatial dynamics proposed by the TIED-ABM requires empirical alignment against high-throughput spatial omics architectures. Advanced diagnostic imaging, including multiplex immunohistochemistry (mIHC) and imaging mass cytometry (IMC), now enables high-dimensional topological mapping within intact structural tissues. By computationally projecting our generated voxel coordinate densities onto these experimentally derived multi-cellular co-localization fields, the precise spatial predictive accuracy of the overarching ABM can be directly validated. Concurrently, incorporating spatially resolved transcriptomics holds profound potential to structurally anchor the dynamically modeled antigen gradients and localized genomic exhaustion neighborhoods. Architecturally coupling the predictive rigor of computational mesoscopic simulations with the visual fidelity of spatial multiplexing will fundamentally establish a more precise theoretical and physical foundation for engineering spatial tumor immunity interventions.

## Supporting information

S1 Appendix Parameter estimation. (PDF)

S2 Appendix BCABC method. (PDF)

S3 Appendix TIED-ABM algorithm. (PDF)

S4 Appendix Marker gene expression in different T cell subsets. (PDF)

S5 Appendix Parameter analysis. (PDF)

S6 Appendix Exhaustion level analysis. (PDF)

S7 Appendix Delay dynamic analysis. (PDF)

S8 Appendix ODE model integrating antigen exposure levels. (PDF)

S1 Video Spatiotemporal simulation of tumor immunity. (GIF)
