# Supplementary material for: Multiscale modeling of T cell exhaustion: A mathematical framework integrating continuous dynamics with spatial heterogeneity
Source: PLoS Comput Biol. 2026 Aug 26;22(8):e1014690. doi: 10.1371/journal.pcbi.1014690 (PMC13541251; doi:10.1371/journal.pcbi.1014690)
Supplement: Supplementary file 1 [file pcbi.1014690.s001.pdf]

S1 Appendix. Parameter estimation

In this study, we establish a mathematical model consisting of 11 categories and 38 kinetic parameters. We determine the parameter values and their ranges based on three key considerations: (1) biological significance, (2) previously published studies, and (3) experimental data. All parameters are summarized in Table A.

Cell proliferation rate (α). Based on dynamic modeling of tumor evolution [1, 2], the proliferation rate of tumor cells in murine models is parameterized as $\alpha_C = 0.680$ day^{-1}. Reported values for this parameter range from 0.6387 day^{-1} to 0.7163 day^{-1}, reflecting variability across different tumor microenvironments. Consistent with [3–5], we take the proliferation rates of tumor-reactive T cells, helper T cells, and regulatory T cells as $\alpha_{T_f} = 0.12$ day^{-1}, $\alpha_{T_h} = 0.15$ day^{-1}, and $\alpha_{T_r} = 0.12$ day^{-1}, respectively. These parameter settings align with experimental observations and provide a foundation for simulating tumor-immune interactions in the model. Since the proliferation level of exhausted T cells is lower than that of effector T cells [6–8], i.e., $\alpha_{T_t} < \alpha_{T_p} < \alpha_{T_f}$, we set $\alpha_{T_p} = 0.075$ day^{-1} and $\alpha_{T_t} = 0.025$ day^{-1}. The difference in parameter values reflects the cell cycle arrest and decreased proliferation capacity of exhausted T cells resulting from persistent antigenic stimulation [6–8].

Carrying capacity (K). According to several studies [9–11], we set the carrying capacity of tumor cells as $K_C = 9 \times 10^8$ cells, with values ranging from 5×10^8 to 2×10^9 cells. This range reflects the inherent heterogeneity across tumor types and microenvironments, arising from differences in nutrient availability, vascularization, and immune infiltration. Furthermore, multiscale modeling and spatial transcriptomics data indicate that tumor cells outnumber CD8+ T cells in the tumor microenvironment [12–14]. Therefore, we set $K_T = 1 \times 10^8$ cells, where K_T denotes the carrying capacity of CD8+ T cells.

Half-saturation constant (E). Following previous studies [15, 16], we establish the half-saturation constant for tumor cells as $E_C = 3.74 \times 10^7$ cells. This parameter represents the tumor cell density at which immune responses reach half-maximal efficacy, a key determinant in tumor-immune dynamics. Experimental measurements [17] demonstrate that helper T cell counts are on the order of 10^5 cells. We parameterize $E_{T_h} = 6 \times 10^5$ cells and $E_{T_rT_h} = 6 \times 10^5$ cells. These values are chosen to reflect the typical steady-state concentrations observed in tumor microenvironments. Consistent with reported regulatory T cell abundances ranging from 10^5 to 10^7 cells [17, 18], we set $E_{T_hT_r} = 1 \times 10^5$ cells and $E_{T_fT_r} = 1 \times 10^7$ cells. $E_{T_rT_f} = 7 \times 10^5$ cells [19]. Through computational fitting to experimental data [20], we determine $E_{pf} = 5 \times 10^6$ cells and $E_{tp} = 5 \times 10^7$ cells.

Death rate (d). According to [21], we parameterize the death rate of tumor cells as $d_C = 0.17$ day^{-1}. The death rates of tumor-reactive T cells, helper T cells and regulatory T cells are $d_{T_f} = 0.18$ day^{-1}, $d_{T_h} = 0.197$ day^{-1}, and $d_{T_r} = 0.125$ day^{-1}, respectively [15, 21–23]. Meanwhile, we set $d_{T_p} = 0.125$ day^{-1} and $d_{T_t} = 0.01$ day^{-1} [8, 24–26].

Killing rate (η). Based on published studies [2, 22, 27–29], we select a CD8+ T cell-mediated tumor killing rate ranging between 1×10^{-9} and 1×10^{-6} cells^{-1}day^{-1}. As exhaustion progresses, the cytotoxic activity of T cells and their ability to secrete effector factors gradually decline [6, 8, 30], i.e., $\eta_{T_f} > \eta_{T_p} > \eta_{T_t}$. Therefore, we assume that $\eta_{T_f} = 7.50 \times 10^{-7}$ cells^{-1}day^{-1} with a range from 1×10^{-7} to 1×10^{-6} cells^{-1}day^{-1}, $\eta_{T_p} = 7.50 \times 10^{-8}$ cells^{-1}day^{-1} with a range from

1×10^{-8} to 1×10^{-7} cells^{-1}day^{-1}, $\eta_{T_t} = 7.50 \times 10^{-9}$ cells^{-1}day^{-1} with a range from 1×10^{-9} to 1×10^{-8} cells^{-1}day^{-1}. The killing rates of tumor-reactive T cells, progenitor exhausted T cells, and terminally exhausted T cells require inference specifically through approximate Bayesian calculations. Meanwhile, the killing rate range shows a gradual decrease in cytotoxic mechanisms, consistent with single-cell sequencing data [31].

Cell baseline level (T). Based on previous studies [5,32], the baseline levels of naïve CD4+ T cells and naïve CD8+ T cells are set as $T_{N4} = T_{N8} = 5.00 \times 10^5$ cells, with a range between 1×10^5 and 1×10^6 cells. This parameter range captures the physiological variation in naïve T cell pools observed across different mice. These values are fixed as constants to represent a steady thymic naïve T cell supply maintained at homeostasis over the simulation period.

Activation rate (λ). Based on previous studies [5, 16, 18, 22, 33], the activation rate of tumor-reactive T cells is identified as $\lambda_{T_f} = 0.6$ day^{-1}, with a range between 0.2 and 1.2 day^{-1}. Additionally, according to studies [22] and [16], the activation rates of helper T cells and regulatory T cells are selected as $\lambda_{T_h} = 1$ day^{-1} and $\lambda_{T_r} = 0.9$ day^{-1}. These differential activation rates reflect the distinct functional requirements and response kinetics of each T cell subset in anti-tumor immunity.

Exhaustion rate (β). Due to mechanisms such as irreversible epigenetic reprogramming, diminished effector function, and metabolic dysfunction in exhausted T cells, the terminal exhaustion rate is higher than the progenitor exhaustion rate [20, 34, 35], i.e., $\beta_{T_pT_f} < \beta_{T_tT_p}$. Based on studies [36–38], we set $\beta_{T_pT_f} = 0.5$ day^{-1} (range : 0-0.75 day^{-1}) and $\beta_{T_tT_p} = 1.0$ day^{-1} (range : 0.75-1.50 day^{-1}). This hierarchical parameterization captures the progressive nature of T cell exhaustion, where terminally exhausted cells exhibit more severe functional impairment and reduced proliferative capacity compared to progenitor exhausted populations.

Hill coefficient (n). In systems biology modeling, the Hill coefficient in the Hill function generally ranges from 0 to 8 [39]. We assign the Hill coefficients for the exhaustion processes of tumor-reactive T cells and progenitor exhausted T cells as $n_1 = 1$ and $n_2 = 1$, respectively.

Exhaustion time (τ). Based on experimental observations reported in [20], approximately 90% of exhausted T cells are progenitor exhausted T cells and only 10% correspond to terminally exhausted T cells on day 6 following tumor inoculation. The complete two-step exhaustion cascade, progressing from functional tumor-reactive T cells to progenitor exhausted T cells and subsequently to terminally exhausted T cells, is accomplished over approximately six days. For modeling simplification, we assume identical transition kinetics for the two sequential differentiation steps. Therefore, the total six-day period is split equally, yielding $\tau_1 = \tau_2 = 3$ days for these two exhaustion processes. This parameter quantifies the characteristic timescale driving progressive T-cell dysfunction upon persistent tumor antigen stimulation.

Efficacy coefficient (γ). Based on experimental data from [20], we set the efficacy coefficients for anti-PD-1 and anti-CTLA-4 as $\gamma_1 = 0.5$ (range : 0-1) and $\gamma_2 = 0.9$ (range : 0.8-1), respectively. Next, we use approximate Bayesian inference to infer the distribution rules of the efficacy parameters, which reflect the heterogeneity of drug responses among different mice.

The dynamic behavior of the system under baseline parameters is illustrated in Figure A.

Table A. Parameter values and definition.

Notation	Description	Value	Range	Units	Reference & Constraint
α_C	Proliferation rate of C	0.680	[0.6387, 0.7163]	day^{-1}	[1, 2]
α_{T_f}	Proliferation rate of T_f	0.120	-	day^{-1}	[3–5]
α_{T_p}	Proliferation rate of T_p	0.075	-	day^{-1}	$\alpha_{T_t} < \alpha_{T_p} < \alpha_{T_f}$ [6–8]
α_{T_t}	Proliferation rate of T_t	0.025	-	day^{-1}	$\alpha_{T_t} < \alpha_{T_p} < \alpha_{T_f}$ [6–8]
α_{T_h}	Proliferation rate of T_h	0.150	-	day^{-1}	[3, 4]
α_{T_r}	Proliferation rate of T_r	0.120	-	day^{-1}	[3, 4]
K_C	Carrying capacity of tumor cells	9.00×10^8	$[5.00 \times 10^8,\ 2 \times 10^9]$	cells	[9–11]
K_T	Carrying capacity of CD8+ T cells	1.00×10^8	-	cells	$K_C < K_T$ [12–14]
E_C	Half-saturation constant of C	3.74×10^7	-	cells	[15, 16]
E_{T_h}	Half-saturation constant of T_h	6.00×10^5	-	cells	Estimated from [17]
E_{pf}	Half-saturation constant of T_f	5.00×10^6	-	cells	Estimated from [20]
E_{tp}	Half-saturation constant of T_p	5.00×10^7	-	cells	Estimated from [20]
$E_{T_f T_r}$	Inhibition of T_f by T_r	1.00×10^7	-	cells	Estimated from [17, 18]
$E_{T_h T_r}$	Inhibition of T_h by T_r	1.00×10^5	-	cells	Estimated from [17, 18]
$E_{T_r T_f}$	Inhibition of T_r by T_f	7.00×10^5	-	cells	[19]
$E_{T_r T_h}$	Inhibition of T_r by T_h	6.00×10^5	-	cells	Estimated from [17]
d_C	Death rate of C	0.170	-	day^{-1}	[21]
d_{T_f}	Death rate of T_f	0.180	-	day^{-1}	[15, 21–23]
d_{T_p}	Death rate of T_p	0.125	-	day^{-1}	Estimated from [8, 24–26]
d_{T_t}	Death rate of T_t	0.010	-	day^{-1}	Estimated from [8, 24–26]
d_{T_h}	Death rate of T_h	0.197	-	day^{-1}	[15, 21–23]
d_{T_r}	Death rate of T_r	0.125	-	day^{-1}	[15, 21–23]
η_{T_f}	Killing rate of C by T_f	7.50×10^{-7}	$[1 \times 10^{-7},\ 1 \times 10^{-6}]$	cells^{-1}day^{-1}	[2, 22, 27–29]
η_{T_p}	Killing rate of C by T_p	7.50×10^{-8}	$[1 \times 10^{-8},\ 1 \times 10^{-7}]$	cells^{-1}day^{-1}	$\eta_{T_t} < \eta_{T_p} < \eta_{T_f}$ [6, 8, 30]
η_{T_t}	Killing rate of C by T_t	7.50×10^{-9}	$[1 \times 10^{-9},\ 1 \times 10^{-8}]$	cells^{-1}day^{-1}	$\eta_{T_t} < \eta_{T_p} < \eta_{T_f}$ [6, 8, 30]
T_{N8}	Naïve CD8+ T cell baseline level	5.00×10^5	-	cells	[5, 32]
T_{N4}	Naïve CD4+ T cell baseline level	5.00×10^5	-	cells	[5, 32]
λ_{T_f}	Activation rate of T_f by C	0.600	[0.2, 1.2]	day^{-1}	[5, 16, 18, 22, 33]
λ_{T_h}	Activation rate of T_h by C	1.000	-	day^{-1}	[16]
λ_{T_r}	Activation rate of T_r by C	0.900	-	day^{-1}	[16, 22]
$\beta_{T_p T_f}$	Exhaustion rate from T_f to T_p	0.50	[0.00, 0.75]	day^{-1}	$\beta_{T_p T_f} < \beta_{T_t T_p}$ [20, 34, 35]
$\beta_{T_t T_p}$	Exhaustion rate from T_p to T_t	1.00	[0.75, 1.50]	day^{-1}	$\beta_{T_p T_f} < \beta_{T_t T_p}$ [20, 34, 35]
n_1	Hill coefficient of T_f exhaustion	1.00	-	none	Estimated from [39]
n_2	Hill coefficient of T_p exhaustion	1.00	-	none	Estimated from [39]
τ_1	Exhaustion time of T_f	3.00	-	day	Estimated from [20]
τ_2	Exhaustion time of T_p	3.00	-	day	Estimated from [20]
γ_1	Efficacy coefficient of anti-CTLA-4	0.5	[0, 1]	none	Estimated from [20]
γ_2	Efficacy coefficient of anti-PD-1	0.9	[0.8, 1]	none	Estimated from [20]

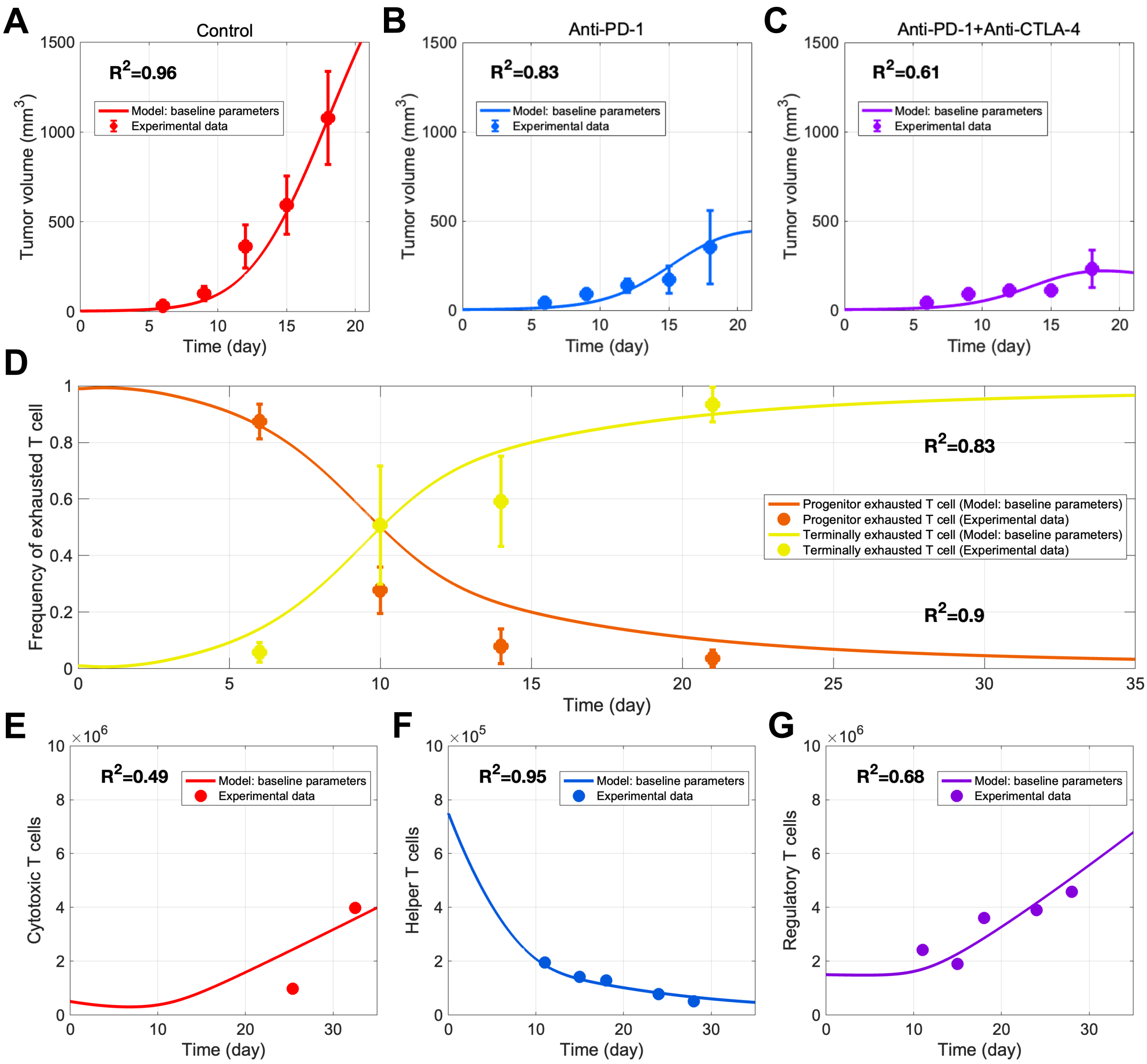

Figure A. The dynamic behavior of the system under baseline parameters.

References

[1] Fuzhang Wang, M Idrees, and Ayesha Sohail. "AI-MCMC" for the parametric analysis of the hormonal therapy of cancer. *Chaos Soliton Fract*, 154:111618, 2022.

[2] Xiulan Lai and Teng Yu. Modeling combination therapies and T cell exhaustion dynamics in the tumor under immune checkpoint blockade. *Bull Math Biol*, 87(9):128, Aug 2025.

[3] Marisabel Rodriguez Messan, Osman N Yogurtcu, Joseph R McGill, Ujwani Nukala, Zuben E Sauna, and Hong Yang. Mathematical model of a personalized neoantigen cancer vaccine and the human immune system. *PLoS Comput Biol*, 17(9):e1009318, Sep 2021.

[4] Munisha Smalley, Michelle Przedborski, Saravanan Thiyagarajan, Moriah Pellowe, Amit Verma, Nilesh Brijwani, Debika Datta, Misti Jain, Basavaraja U Shanthappa, Vidushi Kapoor, Kodaganur S Gopinath, D C Doval, K S Sabitha, Gaspar Taroncher-Oldenburg, Biswanath Majumder, Pradip Majumder, Mohammad Kohandel, and Aaron Goldman. Integrating systems biology and an ex vivo human tumor model elucidates PD-1 blockade response dynamics. *iScience*, 23(6):101229, Jun 2020.

[5] Zixin Zhang, Xiyin Liang, Jianyong Qin, and Jinzhi Lei. Mathematical model of tumor immune microenvironment with application to the combined therapy targeting the PD-1/PD-L1 pathway and IL-10 cytokine antibody. *Theory Biosci*, 144(1):19–43, Feb 2025.

[6] E John Wherry. T cell exhaustion. *Nat Immunol*, 12(6):492–499, Jun 2011.

[7] E John Wherry and Makoto Kurachi. Molecular and cellular insights into T cell exhaustion. *Nat Rev Immunol*, 15(8):486–499, Aug 2015.

[8] Bocheng Wu, Angela N Koehler, and Peter M K Westcott. New opportunities to overcome T cell dysfunction: The role of transcription factors and how to target them. *Trends Biochem Sci*, 49(11):1014–1029, Nov 2024.

[9] Yaqin Shu, Jicai Huang, Yueping Dong, and Yasuhiro Takeuchi. Mathematical modeling and bifurcation analysis of pro-and anti-tumor macrophages. *Appl Math Model*, 88:758–773, 2020.

[10] Raluca Eftimie and Haneen Hamam. Modelling and investigation of the CD4(+) T cells - Macrophages paradox in melanoma immunotherapies. *J Theor Biol*, 420:82–104, May 2017.

[11] Nicholas Lai, Alexis Farman, and Helen M Byrne. The impact of T-cell exhaustion dynamics on tumour-immune interactions and tumour growth. *Bull Math Biol*, 87(5):61, Apr 2025.

[12] John W Hickey, Eran Agmon, Nina Horowitz, Tze-Kai Tan, Matthew Lamore, John B Sunwoo, Markus W Covert, and Garry P Nolan. Integrating multiplexed imaging and multiscale modeling identifies tumor phenotype conversion as a critical component of therapeutic T cell efficacy. *Cell Syst*, 15(4):322–338, Apr 2024.

[13] Shuming Zhang, Atul Deshpande, Babita K Verma, Hanwen Wang, Haoyang Mi, Long Yuan, Won Jin Ho, Elizabeth M Jaffee, Qingfeng Zhu, Robert A Anders, Mark Yarchoan, Luciane T Kagohara, Elana J Fertig, and Aleksander S Popel. Integration of clinical trial spatial multiomics analysis and virtual clinical trials enables immunotherapy response prediction and biomarker discovery. *Cancer Res*, 84(16):2734–2748, Aug 2024.

[14] Sandra Tietscher, Johanna Wagner, Tobias Anzeneder, Claus Langwieder, Martin Rees, Bettina Sobottka, Natalie de Souza, and Bernd Bodenmiller. A comprehensive single-cell map of T cell exhaustion-associated immune environments in human breast cancer. *Nat Commun*, 14(1):98, Jan 2023.

[15] Chenghang Li, Zonghang Ren, Guiyu Yang, and Jinzhi Lei. Mathematical modeling of tumor immune interactions: The role of Anti-FGFR and Anti-PD-1 in the combination therapy. *Bull Math Biol*, 86(9):116, Aug 2024.

[16] Yongzhen Pei, Siqi Han, Changguo Li, Jinzhi Lei, and Fengxi Wen. Data-based modeling of breast cancer and optimal therapy. *J Theor Biol*, 573:111593, Sep 2023.

[17] Milad Qomlaqi, Fariba Bahrami, Maryam Ajami, and Jamshid Hajati. An extended mathematical model of tumor growth and its interaction with the immune system, to be used for developing an optimized immunotherapy treatment protocol. *Math Biosci*, 292:1–9, Oct 2017.

[18] Pariya Khalili and Ramin Vatankhah. Studying the importance of regulatory T cells in chemoimmunotherapy mathematical modeling and proposing new approaches for developing a mathematical dynamic of cancer. *J Theor Biol*, 563:111437, Apr 2023.

[19] Margaret A Myers, Amanda P Smith, Lindey C Lane, David J Moquin, Rosemary Aogo, Stacie Woolard, Paul Thomas, Peter Vogel, and Amber M Smith. Dynamically linking influenza virus infection kinetics, lung injury, inflammation, and disease severity. *Elife*, 10, Jul 2021.

[20] Brian C Miller, Debattama R Sen, Rose Al Abosy, Kevin Bi, Yamini V Virkud, Martin W LaFleur, Kathleen B Yates, Ana Lako, Kristen Felt, Girish S Naik, Michael Manos, Evisa Gjini, Juhi R Kuchroo, Jeffrey J Ishizuka,

Jenna L Collier, Gabriel K Griffin, Seth Maleri, Dawn E Comstock, Sarah A Weiss, Flavian D Brown, Arpit Panda, Margaret D Zimmer, Robert T Manguso, F Stephen Hodi, Scott J Rodig, Arlene H Sharpe, and W Nicholas Haining. Subsets of exhausted CD8(+) T cells differentially mediate tumor control and respond to checkpoint blockade. *Nat Immunol*, 20(3):326–336, Mar 2019.

[21] Xiulan Lai, Andrew Stiff, Megan Duggan, Robert Wesolowski, William E 3rd Carson, and Avner Friedman. Modeling combination therapy for breast cancer with bet and immune checkpoint inhibitors. *Proc Natl Acad Sci USA*, 115(21):5534–5539, May 2018.

[22] Ling Xue, Hongyu Zhang, Xiaoming Zheng, Wei Sun, and Jinzhi Lei. Treatment of melanoma with dendritic cell vaccines and immune checkpoint inhibitors: A mathematical modeling study. *J Theor Biol*, 568:111489, Jul 2023.

[23] Xiulan Lai and Avner Friedman. Mathematical modeling of cancer treatment with radiation and PD-L1 inhibitor. *Sci China Math*, 63:465–484, Feb 2020.

[24] Fabien Franco, Alison Jaccard, Pedro Romero, Yi-Ru Yu, and Ping-Chih Ho. Metabolic and epigenetic regulation of T-cell exhaustion. *Nat Metab*, 2(10):1001–1012, Oct 2020.

[25] Thomas Gebhardt, Simone L Park, and Ian A Parish. Stem-like exhausted and memory CD8(+) T cells in cancer. *Nat Rev Cancer*, 23(11):780–798, Nov 2023.

[26] Josephine R Giles, Shin Foong Ngiow, Sasikanth Manne, Amy E Baxter, Omar Khan, Ping Wang, Ryan Staupe, Mohamed S Abdel-Hakeem, Hua Huang, Divij Mathew, Mark M Painter, Jennifer E Wu, Yinghui Jane Huang, Rishi R Goel, Patrick K Yan, Giorgos C Karakousis, Xiaowei Xu, Tara C Mitchell, Alexander C Huang, and E John Wherry. Shared and distinct biological circuits in effector, memory and exhausted CD8(+) T cells revealed by temporal single-cell transcriptomics and epigenetics. *Nat Immunol*, 23(11):1600–1613, Nov 2022.

[27] Seyed Peyman Shariatpanahi, Seyed Pooya Shariatpanahi, Keivan Madjidzadeh, Moustapha Hassan, and Manuchehr Abedi-Valugerdi. Mathematical modeling of tumor-induced immunosuppression by myeloid-derived suppressor cells: Implications for therapeutic targeting strategies. *J Theor Biol*, 442:1–10, Apr 2018.

[28] Jesse Kreger, Evanthia T Roussos Torres, and Adam L MacLean. Myeloid-derived suppressor-cell dynamics control outcomes in the metastatic niche. *Cancer Immunol Res*, 11(5):614–628, May 2023.

[29] Hannah G Anderson, Gregory P Takacs, Duane C Harris, Yang Kuang, Jeffrey K Harrison, and Tracy L Stepien. Global stability and parameter analysis reinforce therapeutic targets of PD-L1-PD-1 and MDSCs for glioblastoma. *J Math Biol*, 88(1):10, Dec 2023.

[30] Jenna L Collier, Sarah A Weiss, Kristen E Pauken, Debattama R Sen, and Arlene H Sharpe. Not-so-opposite ends of the spectrum: CD8(+) T cell dysfunction across chronic infection, cancer and autoimmunity. *Nat Immunol*, 22(7):809–819, Jul 2021.

[31] Simone A Minnie, Olivia G Waltner, Ping Zhang, Shuichiro Takahashi, Nicole S Nemychenkov, Kathleen S Ensbey, Christine R Schmidt, Samuel R W Legg, Melissa Comstock, Julie R Boiko, Ethan Nelson, Shruti S Bhise, Alec B Wilkens, Motoko Koyama, Madhav V Dhodapkar, Marta Chesi, Stanley R Riddell, Damian J Green, Andrew Spencer, Scott N Furlan, and Geoffrey R Hill. TIM-3(+) CD8 T cells with a terminally exhausted phenotype retain functional capacity in hematological malignancies. *Sci Immunol*, 9(94):eadg1094, Apr 2024.

[32] Zixiao Xiong, Yunfei Xia, Ling Xue, and Jinzhi Lei. Mathematical modelling and optimization of medication regimens for combination immunotherapy of breast cancer. *Bull Math Biol*, 87(7):88, Jun 2025.

[33] Kang-Ling Liao, Kenton D Watt, and Tom Protin. Different mechanisms of CD200-CD200R induce diverse outcomes in cancer treatment. *Math Biosci*, 365:109072, Nov 2023.

[34] E John Wherry, Sang-Jun Ha, Susan M Kaech, W Nicholas Haining, Surojit Sarkar, Vandana Kalia, Shruti Subramaniam, Joseph N Blattman, Daniel L Barber, and Rafi Ahmed. Molecular signature of CD8+ T cell exhaustion during chronic viral infection. *Immunity*, 27(4):670–684, Oct 2007.

[35] Daniela S Thommen, Viktor H Koelzer, Petra Herzig, Andreas Roller, Marcel Trefny, Sarah Dimeloe, Anna Kiialainen, Jonathan Hanhart, Catherine Schill, Christoph Hess, Spasenija Savic Prince, Mark Wiese, Didier Lardinois, Ping-Chih Ho, Christian Klein, Vaios Karanikas, Kirsten D Mertz, Ton N Schumacher, and Alfred Zippelius. A transcriptionally and functionally distinct PD-1(+) CD8(+) T cell pool with predictive potential in non-small-cell lung cancer treated with PD-1 blockade. *Nat Med*, 24(7):994–1004, Jul 2018.

[36] Tyler Simmons and Doron Levy. Modeling the development of cellular exhaustion and tumor-immune stalemate. *Bull Math Biol*, 85(11):106, Sep 2023.

[37] Tyler Simmons and Doron Levy. Targeting CD4+ T cell exhaustion to improve future immunotherapy strategies. *Bull Math Biol*, 87(1):10, Dec 2024.

[38] Irina Kareva and Jana L Gevertz. Mitigating non-genetic resistance to checkpoint inhibition based on multiple states of immune exhaustion. *NPJ Syst Biol Appl*, 10(1):14, Feb 2024.

[39] Steven A Frank. Input-output relations in biological systems: Measurement, information and the Hill equation. *Biol Direct*, 8:31, Dec 2013.
