# Supplementary material for: Multiscale modeling of T cell exhaustion: A mathematical framework integrating continuous dynamics with spatial heterogeneity
Source: PLoS Comput Biol. 2026 Aug 26;22(8):e1014690. doi: 10.1371/journal.pcbi.1014690 (PMC13541251; doi:10.1371/journal.pcbi.1014690)
Supplement: Supplementary file 2 [file pcbi.1014690.s002.pdf]

S2 Appendix. BCABC method

To calibrate the distributions of key unknown parameters in our mathematical model and generate a virtual cohort, we developed a boundary-constrained approximate Bayesian computation (BCABC) method. This method consists of the following four core components.

(1) **Prior distributions**. Based on biological mechanisms and parameter identifiability, we selected the tumor proliferation rate (α_C), tumor carrying capacity (K_C), killing rate of tumor by T cells (η_{T_f}, η_{T_p}, η_{T_t}), T cell activation rate (λ_{T_f}), T cell exhaustion rates ($\beta_{T_pT_f}$, $\beta_{T_tT_p}$), and efficacy coefficients (γ_1, γ_2) to characterize the heterogeneity of tumor-immune interactions. We employed Latin Hypercube Sampling (LHS) to generate 100,000 parameter sets for numerical simulations.

(2) **Approximate Bayesian computation**. Let θ denote the set of parameters to be estimated, with a prior distribution $\pi(\theta)$. Our goal is to approximate the posterior distribution $P(\theta \mid x_0)$, expressed as:

$$P\left(\theta \mid x_0\right) \propto \int K_\epsilon\left(\rho\left(x_*, x_0\right), \mathbb{I}\left(\rho, \rho_0\right)\right) \cdot F\left(x_* \mid \theta\right) \cdot \pi(\theta) d x_* . \tag{1}$$

where x_0 represents the observed data and x_* denotes the model output. The function $F(x_* \mid \theta)$ is the likelihood. The kernel $K_\epsilon\left(\rho\left(x_*, x_0\right), \mathbb{I}\left(\rho, \rho_0\right)\right)$ is a truncated Gaussian kernel, defined as:

$$K_\epsilon\left(\rho(x_*, x_0), \mathbb{I}(\rho, \rho_0)\right) = \exp\left(-\frac{\rho(x_*, x_0)^2}{2\epsilon^2}\right) \times \mathbb{I}(\rho, \rho_0), \tag{2}$$

where $\mathbb{I}\left(\rho, \rho_0\right) = \begin{cases} 1, & \rho\left(x_*, x_0\right) \leq \rho_0 \\ 0, & \rho\left(x_*, x_0\right) > \rho_0 \end{cases}$ is an indicator function. Here, $\rho_0 = 10$ is the tolerance threshold and $\epsilon = \frac{1}{10}\rho_0 = 1$ is the bandwidth parameter.

Conventional unweighted distance metrics assign equal weights to all data points. They fail to handle tumor volume data with a wide value range. Large values corresponding to late-stage tumor growth tend to dominate the fitting process. As a result, fitting errors of small early-stage tumors are overlooked, which further causes systematic bias in parameter estimation. To address this issue, we adopt an adaptive weighting strategy based on data magnitude. The data-adaptive error term $E(t_i)$ normalizes residuals at each time point. It balances the contributions of high and low amplitude observations to the total fitting loss.The distance function $\rho(x_*, x_0)$ is defined as:

$$\rho\left(x_*, x_0\right) = \sum_{i=1}^{n} \left(\frac{x_0(t_i) - x_*\left(t_i \mid \theta\right)}{E(t_i)}\right)^2 . \tag{3}$$

Here, $x_0(t_i)$ denotes the experimentally measured tumor volume at time t_i. $x_*(t_i \mid \theta)$ represents the model output under the parameter set θ. The numerator $x_0(t_i) - x_*(t_i \mid \theta)$ is the prediction error at each observation time point. The denominator $E(t_i)$ represents the scaling normalization coefficient [1,2]:

$$E(t_i) = \begin{cases} \left[x_0(t_i)\right]^\alpha, & x_0(t_i) \geq V_m \\ \left[V_m\right]^\alpha, & x_0(t_i) < V_m \end{cases} . \tag{4}$$

This piecewise weighting design has two key advantages. When the measured tumor volume

exceeds the threshold V_m, the weight scales with tumor volume in a power-law form. This reduces the dominant effect of large late-stage data and prevents the model from only fitting late tumor dynamics. When the measured tumor volume is lower than V_m, a constant minimum weight is applied. It prevents inflated weights caused by noise in low-amplitude early data and avoids overfitting. Following previous studies [1,2], we set $\alpha = 0.84$ and $V_m = 83$ mm^3. This standardized weighting scheme objectively evaluates the overall fitting performance across the entire tumor growth period.

The choice of the tolerance threshold ρ_0 balances the final number of accepted samples and the inclusion rate. We test the performance of the BCABC method at $\rho_0 = 4, 6, 8, 10, 12, 14, 16$ (see Table A). The results show that as ρ_0 increases, the number of accepted samples gradually increases, while the inclusion rate continuously decreases. When $\rho_0 = 10$, the number of accepted samples is 825, which is close to the maximum sample size achievable at a larger threshold (e.g., 881 at $\rho_0 = 16$), and the inclusion rate is 26.81%, which is within an acceptable range. Further increasing ρ_0 causes the inclusion rate to drop significantly below 20%, while the sample size increases by less than 7%. Decreasing ρ_0 leads to a more substantial loss of samples. We choose $\rho_0 = 10$ because it ensures a sufficient sample size while avoiding an excessively low inclusion rate.

Table A. The number of samples generated under different tolerance thresholds ρ_0.

ρ_0 **value**	**ABC method**	**BCABC method**	**Inclusion rate**
$\rho_0 = 4$	654	332	50.76%
$\rho_0 = 6$	1562	656	42.00%
$\rho_0 = 8$	2384	778	32.63%
$\rho_0 = 10$	3077	825	26.81%
$\rho_0 = 12$	3656	857	23.44%
$\rho_0 = 14$	4132	866	20.96%
$\rho_0 = 16$	4537	881	19.42%

(3) **Virtual cohort test**. The virtual cohort generated via approximate Bayesian computation demonstrates a good overall fit. However, the dynamical trajectories of some virtual samples extend beyond the range constrained by the actual observed data. Therefore, we introduce a penalty function:

$$\ell(\theta) = \sum_{i=1}^{n} \max \left[\left(x_*(t_i \mid \theta) - \frac{l_i + u_i}{2} \right)^2 - \left(\frac{u_i}{2} - \frac{l_i}{2} \right)^2, 0 \right], \tag{5}$$

where u_i and l_i represent the upper and lower limits of the experimental determination, respectively. A parameter set θ is rejected if $\ell(\theta) > 0$, indicating that at least one model prediction falls outside the acceptable range. Conversely, it is accepted if $\ell(\theta) = 0$, meaning all predictions lie within the plausible bounds.

Furthermore, considering the larger measurement errors associated with smaller tumor volumes, only the final measured tumor volume was used as the criterion for data inclusion in this filtering step. To identify key parameters that characterize the evolutionary dynamics of tumors under different treatment strategies, we derive the following parameter sets via the penalty function

screening process:

$$\begin{aligned}\Theta_a &= \{\theta \mid \ell(\theta) = 0,\ V_{\text{final}}(\theta) \in [l_a, u_a]\}\,, \\ \Theta_b &= \{\theta \mid \ell(\theta) = 0,\ V_{\text{final}}(\theta) \in [l_b, u_b]\}\,, \\ \Theta_c &= \{\theta \mid \ell(\theta) = 0,\ V_{\text{final}}(\theta) \in [l_c, u_c]\}\,,\end{aligned} \tag{6}$$

where $V_{\text{final}}(\theta)$ denotes the simulated final tumor volume under parameter vector θ. $[l_a, u_a]$, $[l_b, u_b]$, and $[l_c, u_c]$ represent the plausible observed intervals of the final tumor volume for the control group, the anti-PD-1 monotherapy group, and the anti-PD-1 plus anti-CTLA-4 combination therapy group, respectively. Accordingly, we obtain three parameter sets that reflect distinct treatment-specific responses. The intersection set $\Theta = \Theta_a \cap \Theta_b \cap \Theta_c \subset \mathbb{R}^m$ constitutes the final collection of parameters capable of consistently characterizing the dynamical evolution of tumors across the different therapeutic strategies.

(4) **Posterior Distribution**. Through approximate Bayesian computation and virtual cohort filtering, we obtain a set of parameter vectors $\Theta = \{\theta_1, \theta_2, \cdots, \theta_m\}$ with corresponding weights $W = \{\omega_1, \omega_2, \cdots, \omega_m\}$. The weight ω_i for each $\theta_i \in \Theta$ is set to the weighted average of the weights under different treatment strategies. The discretized posterior distribution of the parameters can thus be expressed as:

$$P(\theta \mid x_0) \approx \sum_{k=1}^{m} \omega_k^* \cdot \delta(\theta - \theta_k), \tag{7}$$

where $\omega_k^* = \omega_k / \sum_{k=1}^{m} \omega_k$ denotes the normalized weight, and θ_k represents the k-th parameter sample drawn from the prior distribution $\pi(\theta)$. Here, m is the number of effectively accepted parameter samples. The weight ω_k is derived from the kernel function $K_\epsilon(\rho(x_*, x_0), \mathbb{I}(\rho, \rho_0))$ computed for the k-th sample. The term $\delta(\theta - \theta_k)$ is the Kronecker delta function, defined as:

$$\delta(\theta - \theta_k) = \begin{cases} 1, & \theta = \theta_k \\ 0, & \theta \neq \theta_k \end{cases}. \tag{8}$$

To convert the discrete probability distribution into a continuous one, we apply Gaussian kernel density estimation:

$$f(\theta \mid x^0) = \sum_{k=1}^{m} \omega_k^* \cdot \left(\frac{1}{h\sqrt{2\pi}} \exp\left(-\frac{(\theta - \theta_k)^2}{2h^2} \right) \right). \tag{9}$$

where h is the smoothing parameter.

The prior distributions, ABC posterior distributions, and BCABC posterior distributions of the 10 sampled parameters are shown in Figure A. The pseudocode of the BCABC algorithm is described in Algorithm A. To evaluate the differences among the prior distribution, ABC posterior distribution, and BCABC posterior distribution in parameter estimation, we compared the mean, variance, KL divergence, and JS divergence of 10 parameters (see Figures B, C, and D).

Quantitative analysis shows that, compared with the broad prior distribution, both the ABC and BCABC posterior distributions reduce the mean and variance of the tumor proliferation rate (α_C) and the carrying capacity (K_C), increase the mean of the killing rate of C by T_f (η_{T_f}) and the exhaustion rate from T_f to T_p ($\beta_{T_pT_f}$), reduce the variance of η_{T_f}, the activation rate of T_f by C (λ_{T_f}), $\beta_{T_pT_f}$, the efficacy coefficient of anti-CTLA-4 (γ_1), and the efficacy coefficient of anti-PD-1 (γ_2), and increase the variance of the killing rate of C by T_p (η_{T_p}) and the killing rate of C by T_t (η_{T_t}). Furthermore, the BCABC posterior distribution reduces the mean of η_{T_p}, η_{T_t}, and γ_1

compared with the ABC posterior distribution.

Qualitative analysis indicates that, compared with the broad prior distribution, the KL divergence of η_{T_f}, $\beta_{T_pT_f}$, and λ_{T_f} in the ABC posterior exceeds 0.3, and the JS divergence exceeds or approaches 0.1. In the BCABC posterior distribution, the KL divergence of η_{T_f} and λ_{T_f} exceeds 0.3, and the JS divergence exceeds or approaches 0.1. For all parameters, the KL divergence between the ABC and BCABC posterior distributions does not exceed 0.3, and the JS divergence does not exceed 0.1. This indicates that the ABC and BCABC posterior distributions further adjust the distribution shapes of η_{T_f}, $\beta_{T_pT_f}$, and λ_{T_f}. Meanwhile, BCABC mainly excludes parameter combinations that are statistically significantly different based on the ABC algorithm, without affecting the overall parameter distribution.

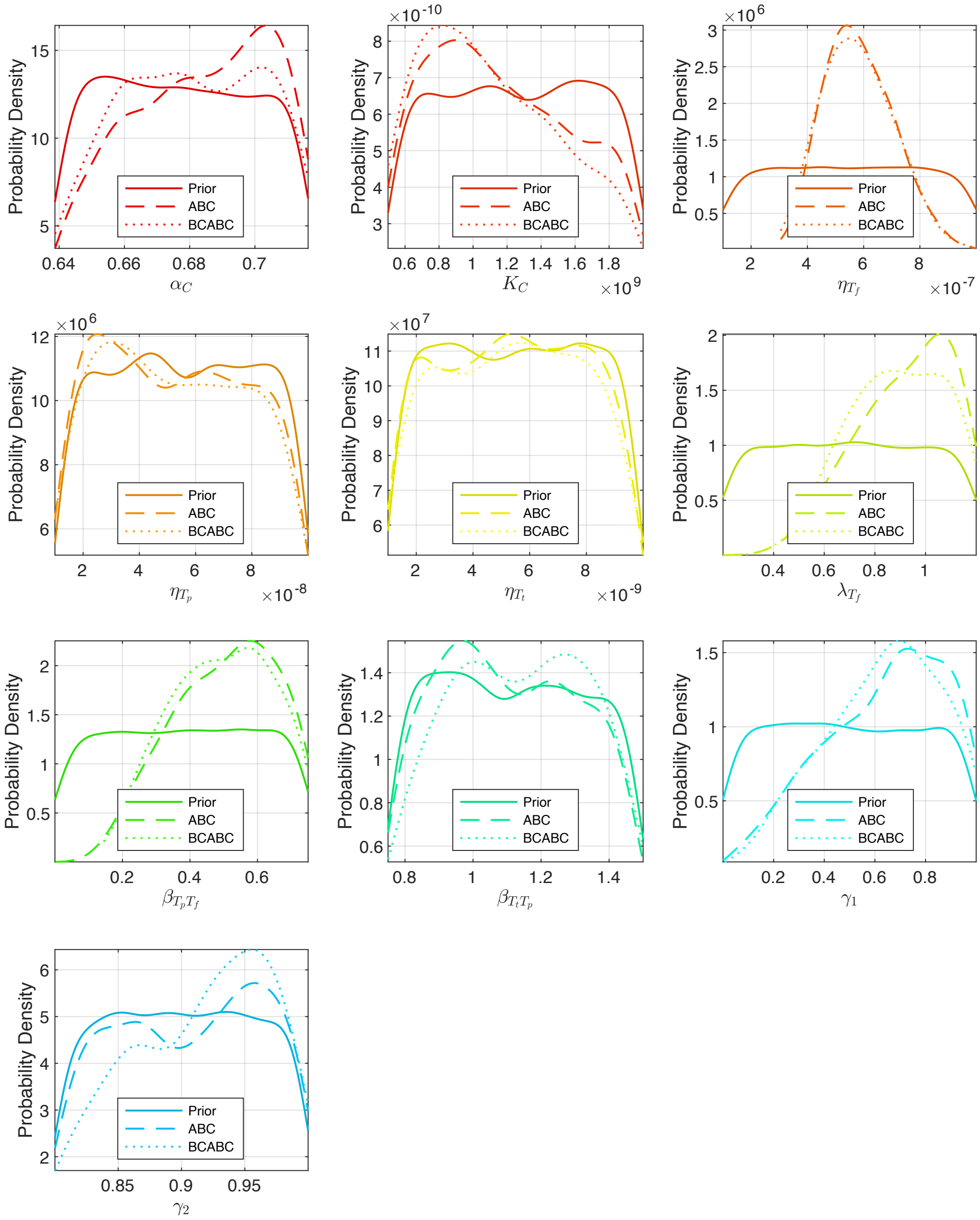

Figure A. The posterior distribution of sampling parameters based on the BCABC method.

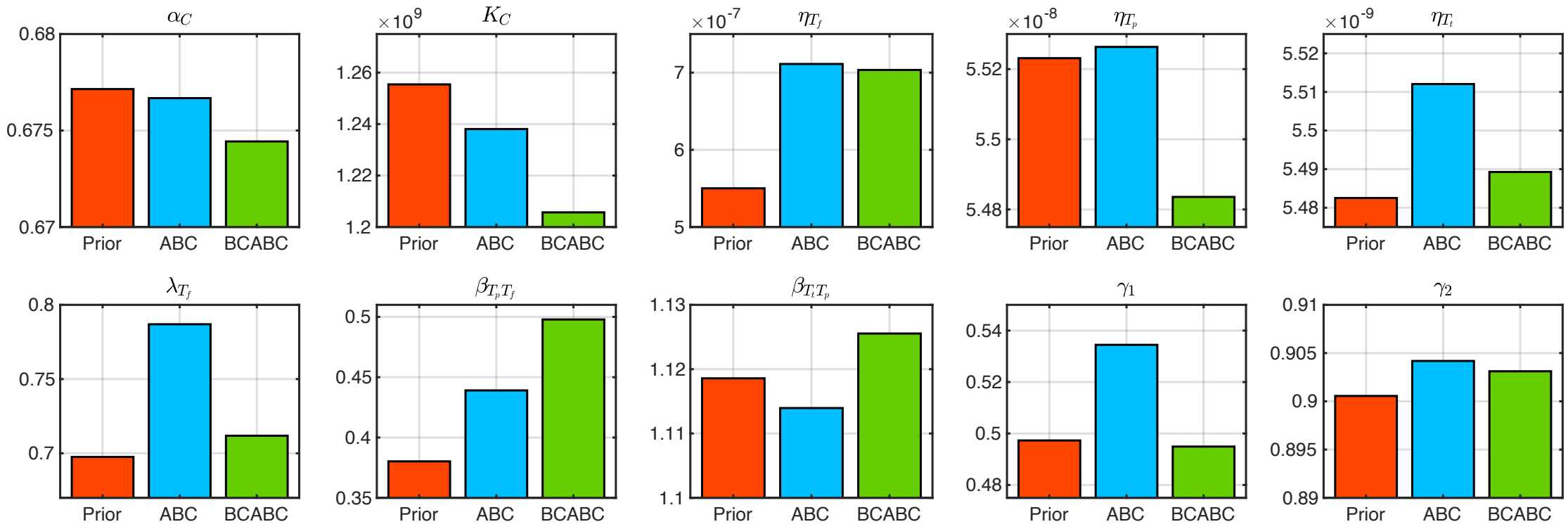

Figure B. The mean of parameters in the prior distribution, ABC posterior distribution, and BCABC posterior distribution.

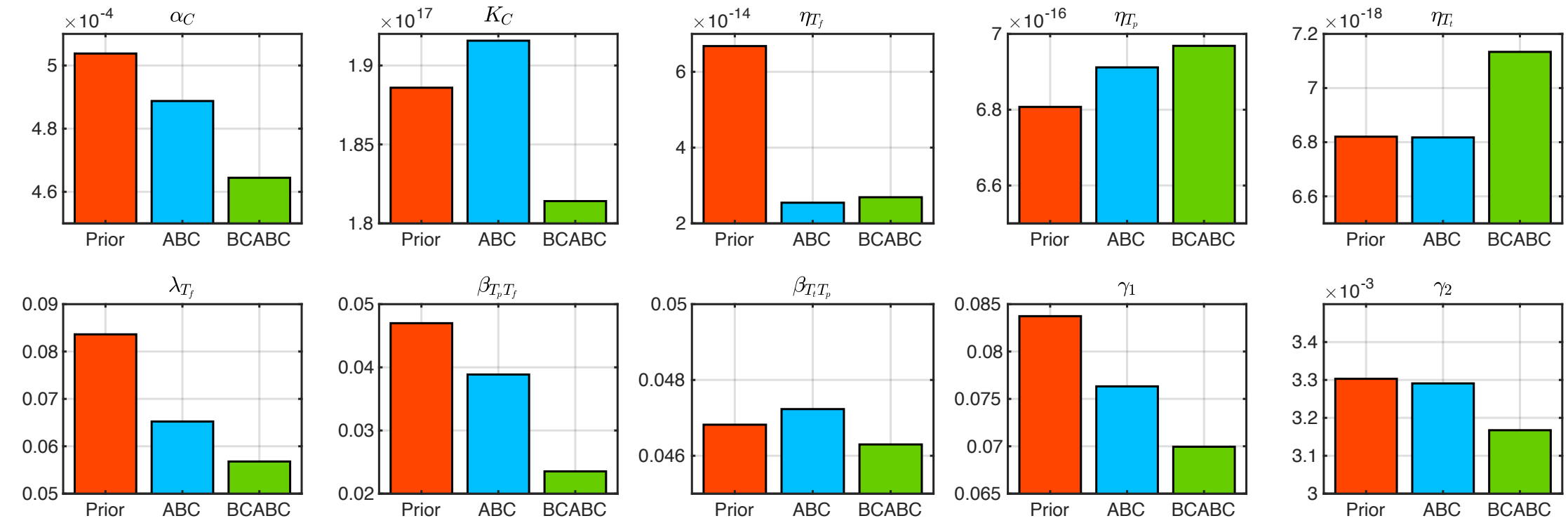

Figure C. The variance of parameters in the prior distribution, ABC posterior distribution, and BCABC posterior distribution.

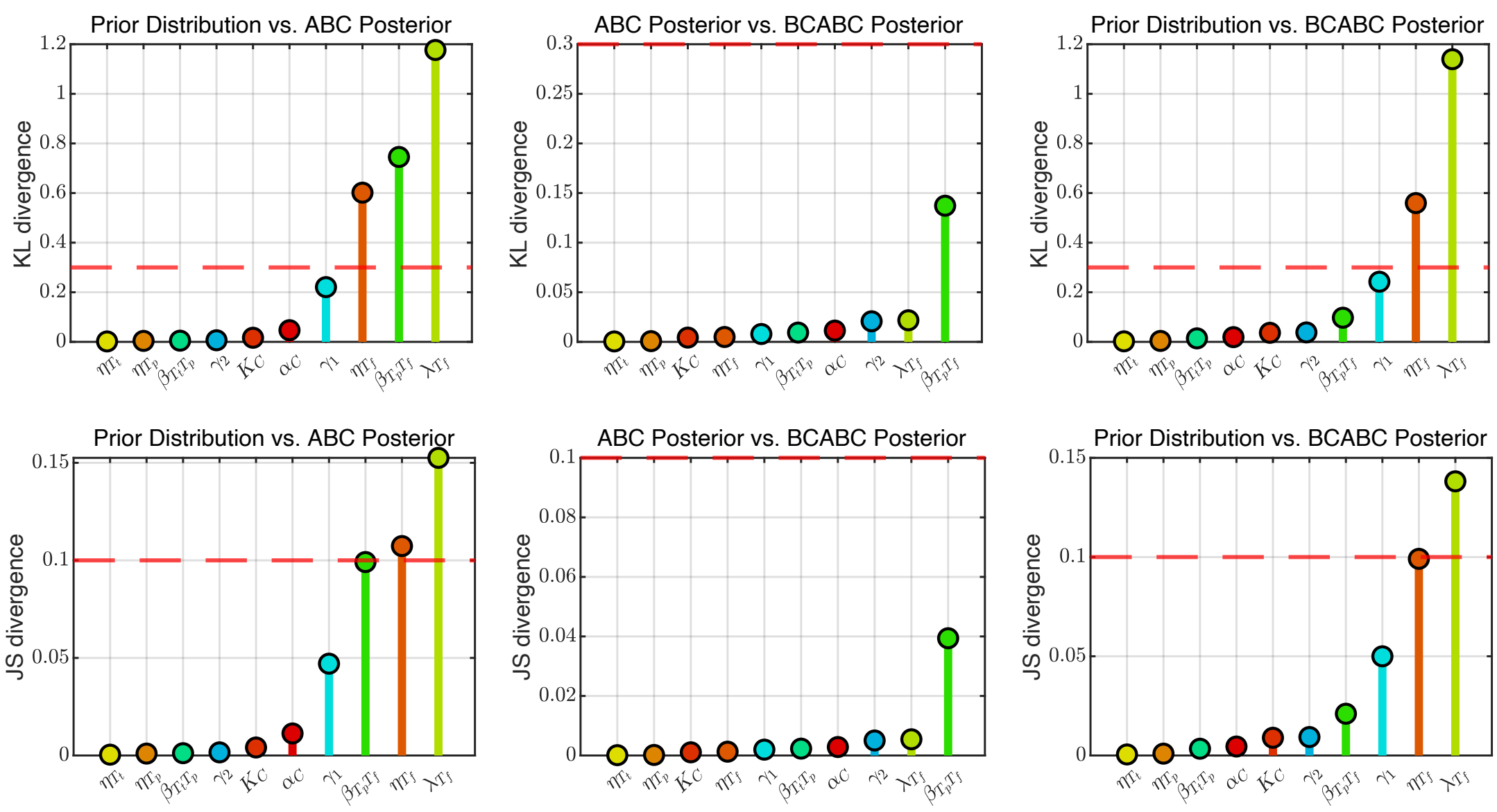

Figure D. The KL divergence and JS divergence of parameters in the prior distribution, ABC posterior distribution, and BCABC posterior distribution.

Algorithm A. Boundary-Constrained Approximate Bayesian Computation (BCABC) method

Require:

Prior ranges for $\theta = (\alpha_C, K_C, \eta_{T_f}, \eta_{T_p}, \eta_{T_t}, \lambda_{T_f}, \beta_{T_pT_f}, \beta_{T_tT_p}, \gamma_1, \gamma_2)$

Observed data x_0 at times t_i $(i = 1, \ldots, 5)$

Tolerance $\rho_0 = 5$, bandwidth $\epsilon = 2.5$, error weight parameters $\alpha = 0.84$, $V_m = 83$ mm^3

Final volume acceptable interval $[l, u]$

Ensure:

Posterior samples $\Theta = \{\theta_k\}$ with normalized weights ω_k^*

Virtual cohort trajectories for all state variables

Step 1: Prior Sampling

Generate $N = 100,000$ parameter samples $\{\theta_1, \theta_2, \ldots, \theta_N\}$ via LHS from the prior $\pi(\theta)$

Step 2: Approximate Bayesian Computation

for each θ_i **do**

  Simulate model $x_*(\cdot \mid \theta_i)$ using the Euler solver

  Extract simulated tumor volumes $\{x_*(t_i \mid \theta_i)\}$ at $t_i = 6, 9, 12, 15, 18$ days

  Compute distance:

$$\rho\big(x_*, x_0\big) = \sum_{i=1}^{5} \left(\frac{x_0(t_i) - x_*(t_i \mid \theta_i)}{E(t_i)} \right)^2, \quad E(t_i) = \begin{cases} \big[x_0(t_i)\big]^{\alpha}, & x_0(t_i) \geq V_m \\ V_m^{\alpha}, & x_0(t_i) < V_m \end{cases}$$

  Define the indicator $\mathbb{I}\big(\rho, \rho_0\big) = \mathbf{1}_{\{\rho \leq \rho_0\}}$ and compute the weight $w_i = \exp\left(-\frac{\rho^2}{2\epsilon^2}\right) \cdot \mathbb{I}\big(\rho, \rho_0\big)$

end for

Keep θ_i only if $w_i > 0$

Step 3: Boundary-Constrained Virtual Cohort Filtering

for each remaining θ_k **do**

  Simulate the final tumor volume $V_{\text{final}}(\theta_k) = x_*(t_{\text{end}} \mid \theta_k)$ at day 18

  Compute the penalty:

$$\ell(\theta_k) = \max\left(\left(V_{\text{final}} - \frac{u+l}{2} \right)^2 - \left(\frac{u-l}{2} \right)^2, \ 0 \right)$$

  Reject θ_k if $\ell(\theta_k) > 0$

  Accept θ_k if $\ell(\theta_k) = 0$

end for

Step 4: Posterior distribution

Normalize weights: $\omega_k^* = \omega_k \big/ \sum_{k=1}^{M} \omega_k$ (where $M = |\Theta|$)

Continuous approximation via Gaussian kernel density estimation:

$$f(\theta \mid x_0) = \sum_{k=1}^{M} \omega_k^* \cdot \frac{1}{h\sqrt{2\pi}} \exp\left(-\frac{(\theta - \theta_k)^2}{2h^2} \right)$$

where h is the smoothing bandwidth.

Step 5: Generate virtual cohort

for each $\theta_k \in \Theta$ **do**

  Simulate the full time course of state variables: $C(t), T_f(t), T_p(t), T_t(t), T_h(t), T_r(t)$

end for

References

[1] Sébastien Benzekry, Clare Lamont, Afshin Beheshti, Amanda Tracz, John M L Ebos, Lynn Hlatky, and Philip Hahnfeldt. Classical mathematical models for description and prediction of experimental tumor growth. *PLoS Comput Biol*, 10(8):e1003800, Aug 2014.

[2] Cristina Vaghi, Anne Rodallec, Raphaëlle Fanciullino, Joseph Ciccolini, Jonathan P Mochel, Michalis Mastri, Clair Poignard, John M L Ebos, and Sébastien Benzekry. Population modeling of tumor growth curves and the reduced Gompertz model improve prediction of the age of experimental tumors. *PLoS Comput Biol*, 16(2):e1007178, Feb 2020.
