# Supplementary material for: Multiscale modeling of T cell exhaustion: A mathematical framework integrating continuous dynamics with spatial heterogeneity
Source: PLoS Comput Biol. 2026 Aug 26;22(8):e1014690. doi: 10.1371/journal.pcbi.1014690 (PMC13541251; doi:10.1371/journal.pcbi.1014690)
Supplement: Supplementary file 3 [file pcbi.1014690.s003.pdf]

S3 Appendix. TIED-ABM algorithm

Mechanistic models based on differential equations often capture only a subset of the complex dynamics in the tumor-immune system. In contrast, agent-based models (ABMs) can incorporate multiple biological processes at the cellular scale, including intercellular communication and spatial competition [1, 2]. To embed biologically grounded mechanisms into an ABM, we developed the TIED-ABM algorithm. Its core innovation is the use of a Poisson process to link macroscopic deterministic equations with stochastic events at the individual cell level, thereby integrating macroscale and microscale dynamics. The TIED-ABM algorithm provides a quantitative framework to investigate the complex bidirectional regulatory interactions within the tumor-immune system.

(1) Model elements. In this study, we primarily integrate the interactions among tumor cells (C), naïve CD8+ T cells (T_{N8}), tumor-reactive T cells (T_f), progenitor exhausted T cells (T_p), terminally exhausted T cells (T_t), naïve CD4+ T cells (T_{N4}), helper T cells (T_h), and regulatory T cells (T_r). Simultaneously, we simulate cellular behaviors through seven key biological mechanisms: proliferation, death, killing, migration, exhaustion, activation, and quiescence (see Figure A). Each process is described by a Poisson distribution with its own rate parameter. Based on previous studies [3–5], we assume that each cell undergoes at most one fundamental biological process within a single time step Δt. The purpose of this assumption is to simplify the model and enhance computational efficiency. It is assumed that Δt is sufficiently small so that the probability of multiple events happening in the same time step is negligible.

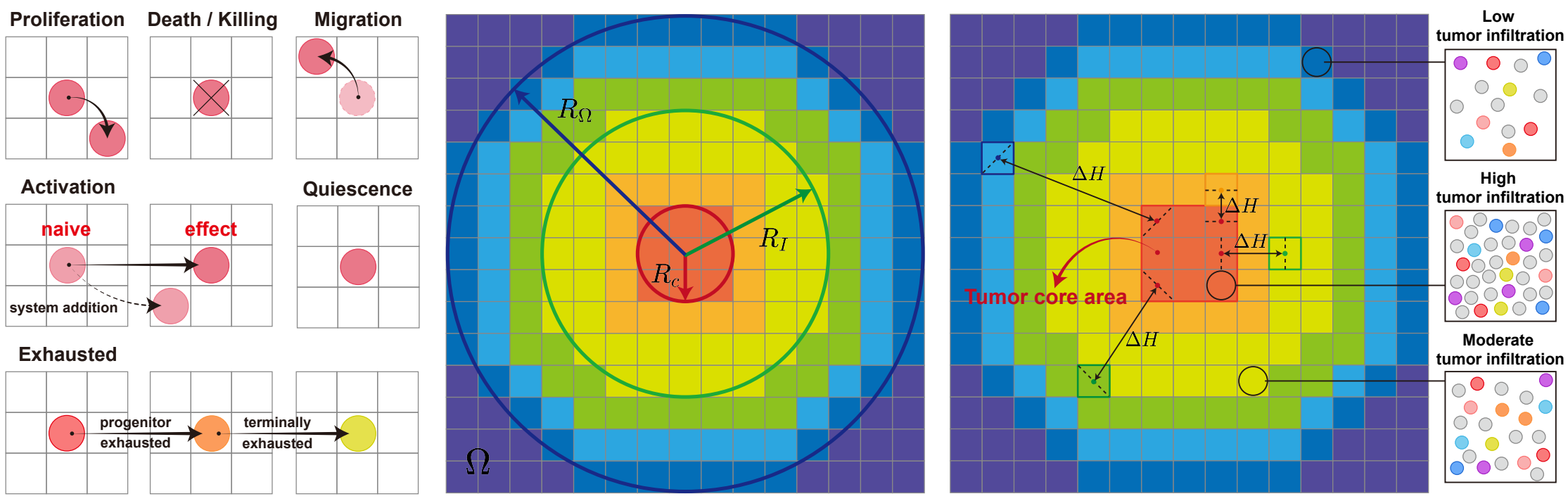

Figure A. Basic setup of the TIED-ABM algorithm. Cell state update mechanisms. Initial delivery radius and diffusion boundaries: R_C (tumor cells), R_T (immune cells), R_Ω (diffusion radius), Ω (voxel space). Voxel distance ΔH and spatial distribution of tumor cells (gray), naïve CD8+ T cells (pink), naïve CD4+ T cells (light blue), helper T cells (dark blue), regulatory T cells (purple), tumor-reactive T cells (red), progenitor exhausted T cells (orange), and terminally exhausted T cells (light green).

(2) Spatial configuration. The initial delivery radii of tumor cells and immune cells are denoted by R_C and R_I, respectively (see Figure A). R_Ω is defined as the boundary radius of spatial diffusion, describing the maximum range that a molecule or particle can reach through random motion in space. We discretized the spatial domain into a two-dimensional grid of voxels (see Figure A). Each voxel contains a 10×10 array of lattice points, representing a cluster of cells that can occupy distinct spatial positions. This design captures spatial heterogeneity across different locations. The dynamic state of each voxel is determined by its cellular occupancy and the surrounding microenvironment. The local environmental carrying capacity N is specified as the

upper limit for the number of cells a single voxel can accommodate. For each voxel, the numbers of C, T_{N8}, T_f, T_p, T_t, T_{N4}, T_h, and T_r are denoted by n_C, $n_{T_{N8}}$, n_{T_f}, n_{T_p}, n_{T_t}, $n_{T_{N4}}$, n_{T_h}, and n_{T_r}, respectively.

(3) Poisson distribution. The Poisson distribution is a key tool in stochastic modeling for describing the probability of discrete events, particularly the number of events occurring within a fixed time interval [6]. In the TIED-ABM algorithm, we assume that state transitions of individual cells within a time step Δt follow a Poisson process. Let λ denote the event rate for a given biological process. Then, the probability that this event occurs exactly k times within Δt is given by the probability mass function:

$$\text{Poisson}(k, \lambda\Delta t) = \frac{(\lambda\Delta t)^k}{k!} e^{-\lambda\Delta t}, \quad k = 0, 1, 2, \dots \tag{1}$$

Here, $\lambda\Delta t$ represents the expected number of events during the interval Δt. The specific event rates λ for each biological process are derived from the TIED model and summarized in Table A.

Table A. Event rate derived from the IDE model

Symbol	Formula expression	Biological description
p^C_{pro}	$\alpha_C\left(1-\frac{C}{K_C}\right)$	Tumor cell proliferation rate
p^C_{kill}	$\eta_{T_f}T_f+\eta_{T_p}T_p+\eta_{T_t}T_t$	Immune cell killing rate
p^C_{death}	d_C	Tumor cell death rate
$p^{T_f}_{\text{act}}$	$\lambda_{T_f}(1+\gamma_1)\frac{C}{E_C+C}\cdot\frac{1}{1+T_r/E_{T_fT_r}}$	Tumor-reactive T cell activation rate
$p^{T_f}_{\text{pro}}$	$\alpha_{T_f}\frac{T_h}{T_h+E_{T_h}}\left(1-\frac{T_f+T_p+T_t}{K_T}\right)$	Tumor-reactive T cell proliferation rate
$p^{T_f}_{\text{death}}$	d_{T_f}	Tumor-reactive T cell death rate
$p^{T_f}_{\text{proexh}}$	$\beta_{T_pT_f}(1-\gamma_2)\frac{\left(\int_{t-\tau_1}^{t}C(s)ds\right)^{n_1}}{E_{pf}^{n_1}+\left(\int_{t-\tau_1}^{t}C(s)ds\right)^{n_1}}$	Tumor-reactive T cell exhaustion rate
$p^{T_p}_{\text{pro}}$	$\alpha_{T_p}\left(1-\frac{T_f+T_p+T_t}{K_T}\right)$	Progenitor exhausted T cell proliferation rate
$p^{T_p}_{\text{death}}$	d_{T_p}	Progenitor exhausted T cell death rate
$p^{T_f}_{\text{proexh}}$	$\beta_{T_tT_p}\frac{\left(\int_{t-\tau_2}^{t}C(s)ds\right)^{n_2}}{E_{tp}^{n_2}+\left(\int_{t-\tau_2}^{t}C(s)ds\right)^{n_2}}$	Progenitor exhausted T cell exhaustion rate
$p^{T_t}_{\text{pro}}$	$\alpha_{T_t}\left(1-\frac{T_f+T_p+T_t}{K_T}\right)$	Terminally exhausted T cell proliferation rate
$p^{T_t}_{\text{death}}$	d_{T_t}	Terminally exhausted T cell death rate
$p^{T_h}_{\text{act}}$	$\lambda_{T_h}\frac{C}{E_C+C}\frac{1}{1+T_r/E_{T_hT_r}}$	Helper T cell activation rate
$p^{T_h}_{\text{pro}}$	$\alpha_{T_h}\frac{T_h}{E_{T_h}+T_h}$	Helper T cell proliferation rate
$p^{T_h}_{\text{death}}$	d_{T_h}	Helper T cell death rate
$p^{T_r}_{\text{act}}$	$\lambda_{T_r}\frac{C}{E_C+C}\frac{1}{1+T_f/E_{T_rT_f}}\frac{1}{1+T_h/E_{T_rT_h}}$	Regulatory T cell activation rate
$p^{T_r}_{\text{pro}}$	$\lambda_{T_r}\frac{C}{E_C+C}\frac{1}{1+T_f/E_{T_rT_f}}\frac{1}{1+T_h/E_{T_rT_h}}$	Regulatory T cell proliferation rate
$p^{T_r}_{\text{death}}$	d_{T_r}	Regulatory T cell death rate

(4) Cell status update.

(4.1) Cell proliferation. Cell proliferation is a core process in tumor growth and immune response. Its rate is mainly affected by spatial competition and resource limitations in the local microenvironment. To simulate density-dependent proliferation, we make two assumptions. First, cells in the same voxel share the same microenvironmental conditions. Second, their proliferative behavior is regulated by local cell density. We define the total number of cells in a voxel (i, j) as

$$n(i,j) = \sum_k n_k(i,j), \quad k \in \{C, T_f, T_p, T_t, T_h, T_r, T_{N8}, T_{N4}\}. \tag{2}$$

The relative cell density is then defined as the ratio of the voxel cell count to the local carrying capacity N:

$$\Delta S(i,j) = \frac{n(i,j)}{N}, \tag{3}$$

which quantifies the degree of crowding within the voxel. Based on previous studies on tumor-immune spatial competition [7], the proliferation probability for cell type Q is given by

$$\mathrm{P}^Q_{\mathrm{pro}} = \mathrm{Poisson}(k, p^Q_{\mathrm{pro}}\Delta t) \times \delta^{\mathrm{pro}}_{\Delta S} \times (1 - \Delta S), \quad Q \in \{C, T_f, T_p, T_t, T_h, T_r\} \tag{4}$$

where $\mathrm{Poisson}(k, p^Q_{\mathrm{pro}}\Delta t)$ is the Poisson probability for the proliferation event occurring exactly k times within Δt. The factor $\delta^{\mathrm{pro}}_{\Delta S}$ modulates the effect of local crowding on proliferation kinetics. Because tumor cells have a higher proliferative capacity than immune cells, we set $\delta^{\mathrm{pro}}_{\Delta S} = 5$ for tumor cells and $\delta^{\mathrm{pro}}_{\Delta S} = 2.5$ for immune cells. The term $1 - \Delta S$ implements density-dependent regulation: as ΔS approaches 1, the proliferation probability tends to zero. This formulation couples stochastic proliferation with local density, allowing rapid expansion in low-density areas and strong inhibition in crowded regions, thereby reproducing the self-limiting nature of cell population growth and resource competition.

(4.2) Cell activation. Naïve CD8+ T cells and naïve CD4+ T cells serve as the initial cell populations driving the adaptive immune response, with their differentiation regulated by the spatial distribution of tumor antigen signals. To quantify this spatial heterogeneity, we define the tumor core region as voxels satisfying the condition:

$$\Delta C = \frac{n_C(i,j)}{N} > 0.5, \tag{5}$$

where $n_C(i, j)$ is the number of tumor cells in the voxel and N is the voxel carrying capacity. For any voxel (i, j), we use its Euclidean distance to the nearest tumor core region, denoted $\Delta H(i, j)$, as a spatial measure of antigen signal strength:

$$\Delta H(i,j) = \min_{(p,q)\in\mathcal{T}} \sqrt{(i-p)^2 + (j-q)^2}, \tag{6}$$

where $\mathcal{T}$ is the set of all tumor core voxels. Based on this, the activation probability for cell type Q (including T_f, T_h, and T_r) is given by:

$$\mathrm{P}^Q_{\mathrm{act}} = \mathrm{Poisson}(k, p^Q_{\mathrm{act}}\Delta t) \times \delta^{\mathrm{act}}_{\Delta H}. \tag{7}$$

Here, $\mathrm{Poisson}(k, p^Q_{\mathrm{act}}\Delta t)$ is the Poisson probability for the activation event occurring exactly k times within Δt. The factor $\delta^{\mathrm{act}}_{\Delta H}$ is a regulatory factor based on the spatial decay of the antigen

signal, modeled by a Hill function:

$$\delta^{\text{act}}_{\Delta H} = \frac{V_{\text{act}} \cdot (K_{\text{act}})^n}{(K_{\text{act}})^n + (\Delta H)^n}, \tag{8}$$

where V_{act} is the maximum activation rate, K_{act} the half-saturation constant, and n the Hill coefficient. For CD8+ T cells, we set $V_{\text{act}} = 3$; for CD4+ T cells, $V_{\text{act}} = 1$. This parameterization reflects the fact that CD8+ T cells, as primary effector cells against tumors, have higher activation requirements than CD4+ T cells, thus capturing their functional differences in the anti-tumor immune response. We set $K_{\text{act}} = 1$ and $n = 3$. This formulation embodies the biological assumption that antigen concentration is high near the tumor region, and the Hill function captures the gradual decay of antigen signals with increasing distance.

- When $\Delta H \to 0$, $\delta^{\text{act}}_{\Delta H} \to V_{\text{act}}$, meaning the antigen concentration is highest in the tumor core and activation reaches its maximum.
- When $\Delta H = K_{\text{act}}$, $\delta^{\text{act}}_{\Delta H} \to V_{\text{act}}/2$, i.e., the activation rate is half of its maximum.
- When $\Delta H \to +\infty$, $\delta^{\text{act}}_{\Delta H} \to 0$, indicating that far from the tumor core the antigen signal vanishes and activation regulation ceases.

Furthermore, we assume that naïve T cells are continuously introduced into the system during the simulation and are stochastically replenished after their activation into various effector T cell subsets. This is consistent with the ongoing process of peripheral T cell homing.

(4.3) Cell exhaustion. T cell exhaustion is a core mechanism of immunosuppression within the tumor microenvironment, typically driven by persistent antigen exposure. To spatially characterize this process, we model the conversion of T_f to T_p (progenitor exhaustion) and T_p to T_t (terminal exhaustion) as stochastic events dependent on the spatial distribution of antigen signals. As previously defined, $\mathcal{T} = \left\{(i,j) \mid \frac{n_C(i,j)}{N} > 0.5\right\}$. For any voxel (i,j), the antigen exposure intensity is represented by the Euclidean distance to the nearest tumor core, ΔH. Based on this, we distinguish two exhaustion pathways for cell types T_f and T_p: progenitor exhaustion and terminal exhaustion. The corresponding probabilities are given by:

$$\text{P}^Q_{\text{proexh}} = \text{Poisson}(k, p^Q_{\text{proexh}} \Delta t) \times \delta^{\text{exh}}_{\Delta H}, \tag{9}$$

$$\text{P}^Q_{\text{terexh}} = \text{Poisson}(k, p^Q_{\text{terexh}} \Delta t) \times \delta^{\text{exh}}_{\Delta H}, \tag{10}$$

where $\text{Poisson}(k, p^Q_{\text{proexh}} \Delta t)$ and $\text{Poisson}(k, p^Q_{\text{terexh}} \Delta t)$ are Poisson probabilities for the exhaustion event occurring exactly k times within Δt. The factor $\delta^{\text{exh}}_{\Delta H}$ is a spatially dependent exhaustion regulatory factor, given by:

$$\delta^{\text{exh}}_{\Delta H} = \frac{V_{\text{exh}} \cdot (K_{\text{exh}})^n}{(K_{\text{exh}})^n + (\Delta H)^n}, \tag{11}$$

where $V_{\text{exh}} = 10$ is the maximum exhaustion rate, $K_{\text{exh}} = 1$ is the half-saturation constant, and $n = 3$ is the Hill coefficient. Both progenitor exhaustion and terminal exhaustion share the same spatial regulatory factor $\delta^{\text{exh}}_{\Delta H}$, reflecting regulation by the same antigenic microenvironment. The differences between the two exhaustion stages are captured by the rate parameters p^Q_{proexh} and p^Q_{terexh}. Thus, this module quantitatively couples T cell exhaustion dynamics with the spatial gradient of antigen distribution, enabling a mechanistic simulation of immune exhaustion heterogeneity at the individual cell scale.

(4.4) Cell death. In the tumor microenvironment, the functional status of T cells significantly influences their survival. We categorize cell death probability $\mathrm{P}_{\text{death}}^{Q}$ into two classes. (1) For functionally normal cells Q_1 (including C, T_f, T_h, and T_r), the death probability depends solely on their intrinsic baseline mortality rate and is modeled as a Poisson process:

$$\mathrm{P}_{\text{death}}^{Q_1} = \text{Poisson}(k, p_{\text{death}}^{Q_1}\Delta t). \tag{12}$$

(2) For dysfunctional cells Q_2 (including T_p and T_t), the death probability is regulated not only by the baseline mortality rate but also by the local tumor cell density ΔC, which simulates the suppressive effect of the tumor microenvironment on exhausted T cell survival:

$$\mathrm{P}_{\text{death}}^{Q_2} = \text{Poisson}(k, p_{\text{death}}^{Q_2}\Delta t) \times \delta_{\Delta C}^{\text{dys}}, \tag{13}$$

where $\delta_{\Delta C}^{\text{dys}}$ is a tumor-density-dependent death enhancement factor, given by the Hill function:

$$\delta_{\Delta C}^{\text{dys}} = \frac{V_{\text{dys}}^{Q_2} \cdot (\Delta C)^n}{(K_{\text{dys}})^n + (\Delta C)^n}. \tag{14}$$

Here, $K_{\text{dys}} = 0.3$ is the half-saturation tumor density, and $n = 3$ is the Hill coefficient. $V_{\text{dys}}^{T_p} = 5$ represents the upper limit of death enhancement for T_p, and $V_{\text{dys}}^{T_t} = 10 \times V_{\text{dys}}^{T_p} = 50$ represents the upper limit for T_t. This reflects that T_t cells suffer from higher metabolic dysregulation and survival vulnerability. Thus, by introducing a tumor-density-dependent death enhancement factor, this module provides a mechanistic characterization of the survival disadvantage of exhausted T cells at the individual cell level.

(4.5) Cell killing. The killing of tumor cells by cytotoxic T lymphocytes is the core effector mechanism of the adaptive immune response against tumors. This process depends not only on the local number of T cells but also on their functional status. To quantitatively characterize spatially dependent killing kinetics, we define the cytotoxic strength of a voxel (i, j) as:

$$\Delta E(i,j) = \varepsilon_{T_f} n_{T_f}(i,j) + \varepsilon_{T_p} n_{T_p}(i,j) + \varepsilon_{T_t} n_{T_t}(i,j), \tag{15}$$

where the weight vector $\boldsymbol{\varepsilon} = (\varepsilon_{T_f}, \varepsilon_{T_p}, \varepsilon_{T_t})^\top = (1, 0.1, 0.01)^\top$ quantifies the cytotoxic potency of each cell type. This assignment reflects the functional hierarchy: T_f cells possess the strongest killing activity, T_p cells show significantly reduced activity, and T_t cells have almost completely lost their killing function. Based on this, the probability of killing a single tumor cell in voxel (i, j) is:

$$\mathrm{P}_{\text{kill}}^{C} = \text{Poisson}(k, p_{\text{kill}}^{C}\Delta t) \times (1 + \delta_{\Delta E}^{\text{kill}}), \tag{16}$$

where $\text{Poisson}(k, p_{\text{kill}}^{C}\Delta t)$ is the Poisson probability for the killing event occurring exactly k times within Δt, and $\delta_{\Delta E}^{\text{kill}}$ is a cytotoxicity regulatory factor given by the Hill function:

$$\delta_{\Delta E}^{\text{kill}} = \frac{V_{\text{kill}} \cdot (\Delta E)^n}{(K_{\text{kill}})^n + (\Delta E)^n}, \tag{17}$$

where $V_{\text{kill}} = 1$ is the maximum killing rate, $K_{\text{kill}} = 5$ is the half-saturation cytotoxic strength, and $n = 3$ is the Hill coefficient. The Hill function captures the saturation of killing efficiency as local cytotoxic strength increases, reflecting the upper limit of effector functions such as perforin and granzyme. Thus, this module provides a mechanistic description of tumor cell killing that

incorporates multi-cell synergy, spatial locality, and saturation kinetics.

(4.6) Cell migration. In the solid tumor microenvironment, the migration of immune cells and tumor cells is regulated by multiple physical and chemical signals. Local spatial crowding is a key factor influencing the direction of cell movement. To characterize density-driven migration, we employ a two-stage stochastic decision process. First, we determine whether a cell migrates during the current time step; if so, we then determine its movement direction based on local crowding.

(1) Migration trigger probability. The probability that a cell Q migrates within a time step Δt is given by:

$$\mathrm{P}_{\mathrm{mig}}^{Q} = 1 - \exp(-\lambda_{\mathrm{mig}}^{Q} \Delta t), \tag{18}$$

where $\lambda_{\mathrm{mig}}^{Q} = \delta_{\Delta S}^{Q,\mathrm{mig}} \Delta S$ is the migration rate, representing the average migratory propensity per unit time. ΔS is the relative cell density of the current voxel, reflecting local crowding, and $\delta_{\Delta S}^{Q,\mathrm{mig}}$ is a cell-type-specific migration modulation coefficient. This formula indicates that the migration rate is proportional to ΔS, meaning cells are more likely to migrate under higher local pressure. Experimental studies show that tumor cells typically have lower migration rates than immune cells due to their epithelial characteristics and enhanced cell-matrix adhesion. To reflect this difference, we set $\delta_{\Delta S}^{C,\mathrm{mig}} = 0.1$ for tumor cells and $\delta_{\Delta S}^{T,\mathrm{mig}} = 1$ for all T cell subtypes.

(2) Migration direction probability. For a cell Q in voxel (i, j), the probability of remaining in place or migrating to any of the eight neighboring voxels is determined by local cell density as follows:

$$\mathrm{P}_{\mathrm{mig},0}^{Q}(i,j) = \frac{1}{\delta_{\mathrm{direction}}} \times \left(1 - \frac{\Delta S(i-1, j-1)}{\Delta \tilde{S}(i,j)}\right), \tag{19}$$

$$\mathrm{P}_{\mathrm{mig},1}^{Q}(i,j) = \frac{1}{\delta_{\mathrm{direction}}} \times \left(1 - \frac{\Delta S(i-1, j)}{\Delta \tilde{S}(i,j)}\right), \tag{20}$$

$$\mathrm{P}_{\mathrm{mig},2}^{Q}(i,j) = \frac{1}{\delta_{\mathrm{direction}}} \times \left(1 - \frac{\Delta S(i-1, j+1)}{\Delta \tilde{S}(i,j)}\right), \tag{21}$$

$$\mathrm{P}_{\mathrm{mig},3}^{Q}(i,j) = \frac{1}{\delta_{\mathrm{direction}}} \times \left(1 - \frac{\Delta S(i, j+1)}{\Delta \tilde{S}(i,j)}\right), \tag{22}$$

$$\mathrm{P}_{\mathrm{mig},4}^{Q}(i,j) = \frac{1}{\delta_{\mathrm{direction}}} \times \left(1 - \frac{\Delta S(i+1, j+1)}{\Delta \tilde{S}(i,j)}\right), \tag{23}$$

$$\mathrm{P}_{\mathrm{mig},5}^{Q}(i,j) = \frac{1}{\delta_{\mathrm{direction}}} \times \left(1 - \frac{\Delta S(i+1, j)}{\Delta \tilde{S}(i,j)}\right), \tag{24}$$

$$\mathrm{P}_{\mathrm{mig},6}^{Q}(i,j) = \frac{1}{\delta_{\mathrm{direction}}} \times \left(1 - \frac{\Delta S(i+1, j-1)}{\Delta \tilde{S}(i,j)}\right), \tag{25}$$

$$\mathrm{P}_{\mathrm{mig},7}^{Q}(i,j) = \frac{1}{\delta_{\mathrm{direction}}} \times \left(1 - \frac{\Delta S(i, j-1)}{\Delta \tilde{S}(i,j)}\right), \tag{26}$$

$$\mathrm{P}_{\mathrm{mig},8}^{Q}(i,j) = \frac{1}{\delta_{\mathrm{direction}}} \times \left(1 - \frac{\Delta S(i, j)}{\Delta \tilde{S}(i,j)}\right), \tag{27}$$

where $\delta_{\mathrm{direction}} = 8$ is the normalized regulatory factor,

$$\Delta S(i,j) = \sum_{k} \frac{n_k(i,j)}{N}, \quad k \in \{C, T_f, T_p, T_t, T_h, T_r, T_{N8}, T_{N4}\} \tag{28}$$

is the relative cell density of voxel (i, j), and

$$\Delta\tilde{S}(i,j) = \sum_{p=i-1}^{i+1} \sum_{q=j-1}^{j+1} \Delta S(p,q) \tag{29}$$

is the sum of densities over the 3×3 neighborhood centered at (i, j). These probabilities satisfy

$$\sum_{n=0}^{8} \mathrm{P}_{\mathrm{mig,n}}^{Q}(i,j) = 1. \tag{30}$$

The migration probability is inversely proportional to the target voxel density, so cells preferentially move toward less crowded regions. This simulates contact inhibition and pressure-driven dispersal, consistent with experimental observations of cells spreading outward from crowded areas.

(4.7) Cell quiescence. In tumor-immune dynamics, not all cells engage in active processes such as proliferation, migration, or death at every time step. Due to factors such as the cell cycle, microenvironmental signals, or resource limitations, some cells may temporarily enter a quiescent state. To capture this biological reality and maintain model completeness within a probabilistic framework, the quiescence probability for a cell Q is defined as:

$$\mathrm{P}_{\mathrm{Qui}}^{Q} = 1 - \sum_{\omega \in \Omega_Q} \mathrm{P}_{\omega}^{Q}, \tag{31}$$

where Ω_Q denotes the set of all possible events for cell Q. For each cell type, Ω_Q is defined as follows:

- Tumor cells (C): $\Omega_C = \{\mathrm{pro},\ \mathrm{death},\ \mathrm{kill},\ \mathrm{mig}\}$;
- Tumor-reactive T cells (T_f): $\Omega_{T_f} = \{\mathrm{pro},\ \mathrm{act},\ \mathrm{proexh},\ \mathrm{death},\ \mathrm{mig}\}$;
- Progenitor exhausted T cells (T_p): $\Omega_{T_p} = \{\mathrm{pro},\ \mathrm{terexh},\ \mathrm{death},\ \mathrm{mig}\}$;
- Terminally exhausted T cells (T_t): $\Omega_{T_t} = \{\mathrm{pro},\ \mathrm{death},\ \mathrm{mig}\}$;
- Helper T cells (T_h): $\Omega_{T_h} = \{\mathrm{pro},\ \mathrm{act},\ \mathrm{death},\ \mathrm{mig}\}$;
- Regulatory T cells (T_r): $\Omega_{T_r} = \{\mathrm{pro},\ \mathrm{act},\ \mathrm{death},\ \mathrm{mig}\}$.

References

[1] Blanche Mongeon, Julien Hébert-Doutreloux, Anudeep Surendran, Elham Karimi, Benoit Fiset, Daniela F Quail, Logan A Walsh, Adrianne L Jenner, and Morgan Craig. Spatial computational modelling illuminates the role of the tumour microenvironment for treating glioblastoma with immunotherapies. *NPJ Syst Biol Appl*, 10(1):91, Aug 2024.

[2] Maisa N G van Genderen, Jeroen Kneppers, Anniek Zaalberg, Elise M Bekers, Andries M Bergman, Wilbert Zwart, and Federica Eduati. Agent-based modeling of the prostate tumor microenvironment uncovers spatial tumor growth constraints and immunomodulatory properties. *NPJ Syst Biol Appl*, 10(1):20, Feb 2024.

[3] Colin G Cess and Stacey D Finley. Multi-scale modeling of macrophage-T cell interactions within the tumor microenvironment. *PLoS Comput Biol*, 16(12):e1008519, Dec 2020.

[4] Heber L Rocha, Boris Aguilar, Michael Getz, Ilya Shmulevich, and Paul Macklin. A multiscale model of immune surveillance in micrometastases gives insights on cancer patient digital twins. *NPJ Syst Biol Appl*, 10(1):144, Dec 2024.

[5] Zeming Liu, Ji Zhang, Liu Hong, Qing Nie, and Xiaoqiang Sun. Multiscale mathematical model-informed reinforcement learning optimizes combination treatment scheduling in glioblastoma evolution. *Sci Adv*, 11(32):eadv3316, Aug 2025.

[6] Xinyu Wang, Youming Li, and Chen Jia. Poisson representation: A bridge between discrete and continuous models of stochastic gene regulatory networks. *J R Soc Interface*, 20(208):20230467, Nov 2023.

[7] Beatriz Ocaña-Tienda, Odelaisy León-Triana, Julián Pérez-Beteta, Juan Jiménez-Sánchez, and Víctor M Pérez-García. Radiation necrosis after radiation therapy treatment of brain metastases: A computational approach. *PLoS Comput Biol*, 20(1):e1011400, Jan 2024.
