# Supplementary material for: Multiscale modeling of T cell exhaustion: A mathematical framework integrating continuous dynamics with spatial heterogeneity
Source: PLoS Comput Biol. 2026 Aug 26;22(8):e1014690. doi: 10.1371/journal.pcbi.1014690 (PMC13541251; doi:10.1371/journal.pcbi.1014690)
Supplement: Supplementary file 4 [file pcbi.1014690.s004.pdf]

S4 Appendix: Marker gene expression in different T cell subsets

To delineate the molecular heterogeneity among T cell differentiation states, we compiled the marker gene expression in Table A.

Table A. Marker gene expression in different T cell subsets.

Gene	Biological function description	T_n	T_f	T_p	T_t	References
Satb1	Highly expressed in naïve T cells to maintain quiescence	+ +	- -	- -	- -	[1]
Bach2	Maintaining quiescence and inhibiting effector differentiation	+ +	- -	- -	- -	[1–4]
Sell	Mediating T cell homing and highly expressing in naïve T cells	+ +	- -	- -	- -	[5]
Ccr7	Mediating lymph node homing and maintaining naïve state	+ +	- -	- -	- -	[5, 6]
Lef1	Maintaining naïve state and inhibiting terminal differentiation	+ +	- -	- -	- -	[5]
Tcf7	Maintaining naïve/memory state and inhibiting effector differentiation	+ +	- -	- -	- -	[5]
Foxp1	Maintaining naïve quiescence and inhibiting overactivation	+ +	-/+	- -	- -	[7, 8]
Il7r	Maintaining homeostatic survival of naïve and memory cells	+ +	-/+	- -	- -	[5]
Itga1	Induced expression during effector phase and mediating tissue residency	- -	+ +	-	-	[9, 10]
Itgb1	Critical for tissue residency, migration, and survival of effector/memory T cells	- -	+ +	-/+	-	[11]
Cx3cr1	As a marker of effector cells, mediating killing and migration	- -	+ +	+	- -	[5, 12]
Tbx21	Driving helper T cell differentiation and IFN-γ production	- -	+ +	-/+	- -	[1, 13–15]
Zeb2	Promoting CD8+ T cell differentiation and counteracting TCF1 function	-	+ +	+	- / +	[1]
Gzmb	Highly expressed during effector phase, mediating killing function	- -	+ +	-/+	- -	[16–18]
Ifng	Encoding the cytokine IFN-γ and driving type 1 immune responses	-	+	+	- / +	[16, 19]
Ccl3	Encoding multifunctional inflammatory chemokines	- -	-	+	+	[19, 20]
Ccl4	Recruiting immune cells and synergistically driving T cell exhaustion	- -	-	+	+	[19, 20]
Cd38	Promoting CD8+ T cell exhaustion and resistance to immunotherapy	- -	-	+	+	[19, 21]
Batf	Promoting differentiation of TCF-1+ precursor cells into effector cells	- -	-	+ +	+	[1, 4, 22]
Eomes	Driving transcription of the anti-apoptotic protein Bcl-2	- -	-	+ +	+ +	[1, 14, 23]
Lag3	Maintaining and deepening the exhausted state of CD8+ T cells	- -	-	+ +	+ +	[24, 25]
Tox	The master switch and core driver of the exhaustion program	- -	-	+ +	+ +	[21, 26]
Tox2	Transcriptional regulator of the exhaustion program	- -	-	+ +	+ +	[21, 26]
Ctla4	Encoding immune checkpoint receptors and driving T cell exhaustion	- -	-	+	+ +	[13, 27]
Pdcd1	Negatively regulating T cell function and driving exhaustion	- -	-	+	+ +	[18, 24, 25, 27]
Tigit	Inhibiting anti-tumor immunity and promoting exhaustion	- -	-	+	+ +	[28–31]
Nr4a2	Key driver of exhaustion program and metabolic regulator	- -	-	+	++	[26, 32, 33]
Il2rb	Driving and maintaining terminal exhaustion of CD8+ T cells	- -	-	+	++	[19, 34]
Tnfrsf9	Promoting CD8+ T cell function and participating in exhaustion induction	- -	-	+	++	[19, 35]
Havcr2	Inhibiting T cell effector function and driving exhaustion	- -	-	-/+	++	[6, 36, 37]
Entpd1	Promoting T cell exhaustion and immune evasion	- -	-	-/+	++	[19, 38, 39]
Ahr	Upregulating immunosuppressive receptors and driving T cell exhaustion	- -	-	-/+	++	[21, 40]

Note: (1) T_n: Naïve CD8+ T cells, T_f: Tumor-reactive T cells, T_p: Progenitor exhausted T cells, T_t: Terminally exhausted T cells. (2) Scoring criteria for expression levels: ++ high expression, + moderate expression, - low expression, - - very low/undetectable expression.

References

[1] Xin Lan, Caitlin C Zebley, and Ben Youngblood. Cellular and molecular waypoints along the path of t cell exhaustion. *Sci Immunol*, 8(87):eadg3868, Sep 2023.

[2] Xinyu Weng, Min Zheng, Yanning Liu, and Guohua Lou. The role of Bach2 in regulating CD8 + T cell development and function. *Cell Commun Signal*, 22(1):169, Mar 2024.

[3] Chen Yao, Guohua Lou, Hong-Wei Sun, Ziang Zhu, Yi Sun, Zeyu Chen, Daniel Chauss, E Ashley Moseman, Jun Cheng, Marc A D'Antonio, Wangke Shi, Junwei Shi, Kohei Kometani, Tomohiro Kurosaki, E John Wherry, Behdad Afzali, Luca Gattinoni, Yuwen Zhu, Dorian B McGavern, John J O'Shea, Pamela L Schwartzberg, and Tuoqi Wu. BACH2 enforces the transcriptional and epigenetic programs of stem-like CD8(+) T cells. *Nat Immunol*, 22(3):370–380, Mar 2021.

[4] Daniel T Utzschneider, Sarah S Gabriel, David Chisanga, Renee Gloury, Patrick M Gubser, Ajithkumar Vasanthakumar, Wei Shi, and Axel Kallies. Early precursor T cells establish and propagate T cell exhaustion in chronic infection. *Nat Immunol*, 21(10):1256–1266, Oct 2020.

[5] Anne M van der Leun, Daniela S Thommen, and Ton N Schumacher. CD8(+) T cell states in human cancer: Insights from single-cell analysis. *Nat Rev Cancer*, 20(4):218–232, Apr 2020.

[6] Hong Sheng Quah, Elaine Yiqun Cao, Lisda Suteja, Constance H Li, Hui Sun Leong, Fui Teen Chong, Shilpi Gupta, Camille Arcinas, John F Ouyang, Vivian Ang, Teja Celhar, Yunqian Zhao, Hui Chen Tay, Jerry Chan, Takeshi Takahashi, Daniel S W Tan, Subhra K Biswas, Owen J L Rackham, and N Gopalakrishna Iyer. Single cell analysis in head and neck cancer reveals potential immune evasion mechanisms during early metastasis. *Nat Commun*, 14(1):1680, Mar 2023.

[7] Yaroslav Kaminskiy, Varvara Kuznetsova, Anna Kudriaeva, Ekaterina Zmievskaya, and Emil Bulatov. Neglected, yet significant role of FOXP1 in T-cell quiescence, differentiation and exhaustion. *Front Immunol*, 13:971045, 2022.

[8] Xiaoming Feng, Haikun Wang, Hiroshi Takata, Timothy J Day, Jessica Willen, and Hui Hu. Transcription factor Foxp1 exerts essential cell-intrinsic regulation of the quiescence of naive T cells. *Nat Immunol*, 12(6):544–550, Jun 2011.

[9] Shannon K Bromley, Hasan Akbaba, Vinidhra Mani, Rut Mora-Buch, Alexandra Y Chasse, Andrea Sama, and Andrew D Luster. CD49a regulates cutaneous resident memory CD8(+) T cell persistence and response. *Cell Rep*, 32(9):108085, Sep 2020.

[10] Stanley Cheuk, Heinrich Schlums, Irène Gallais Sérézal, Elisa Martini, Samuel C Chiang, Nicole Marquardt, Anna Gibbs, Ebba Detlofsson, Andrea Introini, Marianne Forkel, Charlotte Höög, Annelie Tjernlund, Jakob Michaëlsson, Lasse Folkersen, Jenny Mjösberg, Lennart Blomqvist, Marcus Ehrström, Mona Ståhle, Yenan T Bryceson, and Liv Eidsmo. CD49a expression defines tissue-resident CD8(+) T cells poised for cytotoxic function in human skin. *Immunity*, 46(2):287–300, Feb 2017.

[11] Benoît P Nicolet, Aurélie Guislain, Floris P J van Alphen, Raquel Gomez-Eerland, Ton N M Schumacher, Maartje van den Biggelaar, and Monika C Wolkers. CD29 identifies IFN-γ-producing human CD8(+) T cells with an increased cytotoxic potential. *Proc Natl Acad Sci USA*, 117(12):6686–6696, Mar 2020.

[12] Jean-Christophe Beltra, Mohamed S Abdel-Hakeem, Sasikanth Manne, Zhen Zhang, Hua Huang, Makoto Kurachi, Leon Su, Lora Picton, Shin Foong Ngiow, Yuki Muroyama, Valentina Casella, Yinghui J Huang, Josephine R Giles, Divij Mathew, Jonathan Belman, Max Klapholz, Hélène Decaluwe, Alexander C Huang, Shelley L Berger, K Christopher Garcia, and E John Wherry. Stat5 opposes the transcription factor Tox and rewires exhausted CD8(+) T cells toward durable effector-like states during chronic antigen exposure. *Immunity*, 56(12):2699–2718, Dec 2023.

[13] Andrew Baessler and Dario A A Vignali. T cell exhaustion. *Annu Rev Immunol*, 42(1):179–206, Jun 2024.

[14] Laura M McLane, Shin Foong Ngiow, Zeyu Chen, John Attanasio, Sasikanth Manne, Gordon Ruthel, Jennifer E Wu, Ryan P Staupe, Wei Xu, Ravi K Amaravadi, Xiaowei Xu, Giorgos C Karakousis, Tara C Mitchell, Lynn M Schuchter, Alexander C Huang, Bruce D Freedman, Michael R Betts, and E John Wherry. Role of nuclear localization in the regulation and function of T-bet and Eomes in exhausted CD8 T cells. *Cell Rep*, 35(6):109120, May 2021.

[15] Charlly Kao, Kenneth J Oestreich, Michael A Paley, Alison Crawford, Jill M Angelosanto, Mohammed-Alkhatim A Ali, Andrew M Intlekofer, Jeremy M Boss, Steven L Reiner, Amy S Weinmann, and E John Wherry. Transcription factor T-bet represses expression of the inhibitory receptor PD-1 and sustains virus-specific CD8+ T cell responses during chronic infection. *Nat Immunol*, 12(7):663–671, May 2011.

[16] Axel Kallies, Dietmar Zehn, and Daniel T Utzschneider. Precursor exhausted T cells: Key to successful immunotherapy? *Nat Rev Immunol*, 20(2):128–136, Feb 2020.

[17] Imran Siddiqui, Karin Schaeuble, Vijaykumar Chennupati, Silvia A Fuertes Marraco, Sandra Calderon-Copete, Daniela Pais Ferreira, Santiago J Carmona, Leonardo Scarpellino, David Gfeller, Sylvain Pradervand, Sanjiv A Luther, Daniel E Speiser, and Werner Held. Intratumoral Tcf1(+)PD-1(+)CD8(+) T cells with stem-like properties promote tumor control in response to vaccination and checkpoint blockade immunotherapy. *Immunity*, 50(1):195–211, Jan 2019.

[18] Thomas Gebhardt, Simone L Park, and Ian A Parish. Stem-like exhausted and memory CD8(+) T cells in cancer. *Nat Rev Cancer*, 23(11):780–798, Nov 2023.

[19] Brian C Miller, Debattama R Sen, Rose Al Abosy, Kevin Bi, Yamini V Virkud, Martin W LaFleur, Kathleen B Yates, Ana Lako, Kristen Felt, Girish S Naik, Michael Manos, Evisa Gjini, Juhi R Kuchroo, Jeffrey J Ishizuka, Jenna L Collier, Gabriel K Griffin, Seth Maleri, Dawn E Comstock, Sarah A Weiss, Flavian D Brown, Arpit Panda, Margaret D Zimmer, Robert T Manguso, F Stephen Hodi, Scott J Rodig, Arlene H Sharpe, and W Nicholas Haining. Subsets of exhausted CD8(+) T cells differentially mediate tumor control and respond to checkpoint blockade. *Nat Immunol*, 20(3):326–336, Mar 2019.

[20] Xinyu Su, Rong Huang, Donglin Kang, Jiayu Wang, Lin Li, and Zhengyun Zou. Blocking CCR1(+) macrophages overcomes resistance to immune checkpoint inhibitors in melanoma. *Cell Commun Signal*, 23(1):475, Nov 2025.

[21] Andrew C Scott, Friederike Dündar, Paul Zumbo, Smita S Chandran, Christopher A Klebanoff, Mojdeh Shakiba, Prerak Trivedi, Laura Menocal, Heather Appleby, Steven Camara, Dmitriy Zamarin, Tyler Walther, Alexandra Snyder, Matthew R Femia, Elizabeth A Comen, Hannah Y Wen, Matthew D Hellmann, Niroshana Anandasabapathy, Yong Liu, Nasser K Altorki, Peter Lauer, Olivier Levy, Michael S Glickman, Jonathan Kaye, Doron Betel, Mary Philip, and Andrea Schietinger. TOX is a critical regulator of tumour-specific T cell differentiation. *Nature*, 571(7764):270–274, Jul 2019.

[22] Yao Chen, Ryan A Zander, Xiaopeng Wu, David M Schauder, Moujtaba Y Kasmani, Jian Shen, Shikan Zheng, Robert Burns, Elizabeth J Taparowsky, and Weiguo Cui. BATF regulates progenitor to cytolytic effector CD8(+) T cell transition during chronic viral infection. *Nat Immunol*, 22(8):996–1007, Aug 2021.

[23] Jing Li, Yi He, Jing Hao, Ling Ni, and Chen Dong. High levels of eomes promote exhaustion of anti-tumor CD8(+) T cells. *Front Immunol*, 9:2981, 2018.

[24] Lawrence P Andrews, Samuel C Butler, Jian Cui, Anthony R Cillo, Carly Cardello, Chang Liu, Erin A Brunazzi, Andrew Baessler, Bingxian Xie, Sheryl R Kunning, Shin Foong Ngiow, Yinghui Jane Huang, Sasikanth Manne, Arlene H Sharpe, Greg M Delgoffe, E John Wherry, John M Kirkwood, Tulia C Bruno, Creg J Workman, and Dario A A Vignali. LAG-3 and PD-1 synergize on CD8(+) T cells to drive T cell exhaustion and hinder autocrine IFN-γ-dependent anti-tumor immunity. *Cell*, 187(16):4355–4372, Aug 2024.

[25] Seng-Ryong Woo, Meghan E Turnis, Monica V Goldberg, Jaishree Bankoti, Mark Selby, Christopher J Nirschl, Matthew L Bettini, David M Gravano, Peter Vogel, Chih Long Liu, Stephanie Tangsombatvisit, Joseph F Grosso, George Netto, Matthew P Smeltzer, Alcides Chaux, Paul J Utz, Creg J Workman, Drew M Pardoll, Alan J Korman, Charles G Drake, and Dario A A Vignali. Immune inhibitory molecules LAG-3 and PD-1 synergistically regulate T-cell function to promote tumoral immune escape. *Cancer Res*, 72(4):917–927, Feb 2012.

[26] Hyungseok Seo, Joyce Chen, Edahí González-Avalos, Daniela Samaniego-Castruita, Arundhoti Das, Yueqiang H Wang, Isaac F López-Moyado, Romain O Georges, Wade Zhang, Atsushi Onodera, Cheng-Jang Wu, Li-Fan Lu, Patrick G Hogan, Avinash Bhandoola, and Anjana Rao. TOX and TOX2 transcription factors cooperate with NR4A transcription factors to impose CD8(+) T cell exhaustion. *Proc Natl Acad Sci USA*, 116(25):12410–12415, Jun 2019.

[27] Xinyi Guo, Yuanyuan Zhang, Liangtao Zheng, Chunhong Zheng, Jintao Song, Qiming Zhang, Boxi Kang, Zhouzerui Liu, Liang Jin, Rui Xing, Ranran Gao, Lei Zhang, Minghui Dong, Xueda Hu, Xianwen Ren, Dennis Kirchhoff, Helge Gottfried Roider, Tiansheng Yan, and Zemin Zhang. Global characterization of T cells in non-small-cell lung cancer by single-cell sequencing. *Nat Med*, 24(7):978–985, Jul 2018.

[28] Tengqian Tang, Wenhao Wang, Lang Gan, Jie Bai, Dehong Tan, Yan Jiang, Ping Zheng, Weijun Zhang, Yu He, Qianfei Zuo, and Leida Zhang. TIGIT expression in extrahepatic cholangiocarcinoma and its impact on CD8 + T cell exhaustion: implications for immunotherapy. *Cell Death Dis*, 16(1):90, Feb 2025.

[29] Rongpu Liang, Xudong Zhu, Tianyun Lan, Dongbing Ding, Zongheng Zheng, Tufeng Chen, Yong Huang, Jianpei Liu, Xiaofeng Yang, Jun Shao, Hongbo Wei, and Bo Wei. TIGIT promotes CD8(+)T cells exhaustion and predicts poor prognosis of colorectal cancer. *Cancer Immunol Immunother*, 70(10):2781–2793, Oct 2021.

[30] Lei Wu, Liang Mao, Jian-Feng Liu, Lei Chen, Guang-Tao Yu, Lei-Lei Yang, Hao Wu, Lin-Lin Bu, Ashok B Kulkarni, Wen-Feng Zhang, and Zhi-Jun Sun. Blockade of TIGIT/CD155 signaling reverses T-cell exhaustion and enhances antitumor capability in head and neck squamous cell carcinoma. *Cancer Immunol Res*, 7(10):1700–1713, Oct 2019.

[31] Dmitrij Ostroumov, Steven Duong, Jessica Wingerath, Norman Woller, Michael P Manns, Kai Timrott, Moritz Kleine, Wolf Ramackers, Stephanie Roessler, Sven Nahnsen, Stefan Czemmel, Oliver Dittrich-Breiholz, Tobias Eggert, Florian Kühnel, and Thomas C Wirth. Transcriptome profiling identifies TIGIT as a marker of T-cell exhaustion in liver cancer. *Hepatology*, 73(4):1399–1418, Apr 2021.

[32] Marilyn Giordano, Coralie Henin, Julien Maurizio, Claire Imbratta, Pierre Bourdely, Michel Buferne, Lukas Baitsch, Laurent Vanhille, Michael H Sieweke, Daniel E Speiser, Nathalie Auphan-Anezin, Anne-Marie Schmitt-Verhulst, and Grégory Verdeil. Molecular profiling of CD8 T cells in autochthonous melanoma identifies Maf as driver of exhaustion. *EMBO J*, 34(15):2042–2058, Aug 2015.

[33] Joyce Chen, Isaac F López-Moyado, Hyungseok Seo, Chan-Wang J Lio, Laura J Hempleman, Takashi Sekiya, Akihiko Yoshimura, James P Scott-Browne, and Anjana Rao. NR4A transcription factors limit CAR T cell function in solid tumours. *Nature*, 567(7749):530–534, Mar 2019.

[34] Jean-Christophe Beltra, Sara Bourbonnais, Nathalie Bédard, Tania Charpentier, Moana Boulangé, Eva Michaud, Ines Boufaied, Julie Bruneau, Naglaa H Shoukry, Alain Lamarre, and Hélène Decaluwe. Il2rβ-dependent signals drive terminal exhaustion and suppress memory development during chronic viral infection. *Proc Natl Acad Sci USA*, 113(37):E5444–53, Sep 2016.

[35] Se Jin Im, Masao Hashimoto, Michael Y Gerner, Junghwa Lee, Haydn T Kissick, Matheus C Burger, Qiang Shan, J Scott Hale, Judong Lee, Tahseen H Nasti, Arlene H Sharpe, Gordon J Freeman, Ronald N Germain, Helder I Nakaya, Hai-Hui Xue, and Rafi Ahmed. Defining CD8+ T cells that provide the proliferative burst after PD-1 therapy. *Nature*, 537(7620):417–421, Sep 2016.

[36] Kaori Sakuishi, Lionel Apetoh, Jenna M Sullivan, Bruce R Blazar, Vijay K Kuchroo, and Ana C Anderson. Targeting Tim-3 and PD-1 pathways to reverse T cell exhaustion and restore anti-tumor immunity. *J Exp Med*, 207(10):2187–2194, Sep 2010.

[37] Jean-Christophe Beltra, Sasikanth Manne, Mohamed S Abdel-Hakeem, Makoto Kurachi, Josephine R Giles, Zeyu Chen, Valentina Casella, Shin Foong Ngiow, Omar Khan, Yinghui Jane Huang, Patrick Yan, Kito Nzingha, Wei Xu, Ravi K Amaravadi, Xiaowei Xu, Giorgos C Karakousis, Tara C Mitchell, Lynn M Schuchter, Alexander C Huang, and E John Wherry. Developmental relationships of four exhausted cd8(+) t cell subsets reveals underlying transcriptional and epigenetic landscape control mechanisms. *Immunity*, 52(5):825–841, May 2020.

[38] Shilpa Tiwari-Heckler, Ghee Rye Lee, James Harbison, Carola Ledderose, Eva Csizmadia, David Melton, Quanzhi Zhang, Wolfgang Junger, Guanqing Chen, Carl J Hauser, Leo E Otterbein, Maria Serena Longhi, and Simon Christopher Robson. Extracellular mitochondria drive CD8 T cell dysfunction in trauma by upregulating CD39. *Thorax*, 78(2):151–159, Feb 2023.

[39] Marius Witt, Leticia Oliveira-Ferrer, Friedrich Koch-Nolte, Stephan Menzel, Louisa Hell, Tabea Sturmheit, Elisa Seubert, Pauline Weimer, Yi Ding, Minyue Qi, Barbara Schmalfeldt, Carsten Bokemeyer, Walter Fiedler, Jasmin Wellbrock, and Franziska Brauneck. Expression of CD39 is associated with T cell exhaustion in ovarian cancer and its blockade reverts T cell dysfunction. *Oncoimmunology*, 13(1):2346359, 2024.

[40] Yifei Zhu, Haoji Wang, Yanxi Yao, Hongdou Ding, Li Xu, and Xinnan Xu. SLC1A5-mediated kynurenine metabolism drives AHR-FANCD2 axis to remodel chromatin and induce T cell exhaustion in lung adenocarcinoma. *Cell Commun Signal*, Feb 2026.
