# Supplementary material for: Multiscale modeling of T cell exhaustion: A mathematical framework integrating continuous dynamics with spatial heterogeneity
Source: PLoS Comput Biol. 2026 Aug 26;22(8):e1014690. doi: 10.1371/journal.pcbi.1014690 (PMC13541251; doi:10.1371/journal.pcbi.1014690)
Supplement: Supplementary file 5 [file pcbi.1014690.s005.pdf]

S5 Appendix. Parameter analysis

(1) Comprehensive sensitivity analysis. To further evaluate the influence of individual parameter perturbations on the overall model fitting performance, we employed a one-at-a-time perturbation strategy. Let the baseline parameter set be $\boldsymbol{\theta}^* = (\theta_1^*, \theta_2^*, \ldots, \theta_M^*)^\top$. For the j-th parameter, the perturbed parameter vector is defined such that only the j-th component is altered:

$$\boldsymbol{\theta}^{(j,k)} = \left(\theta_1^*, \ldots, \theta_{j-1}^*, (1+\varepsilon_k)\theta_j^*, \theta_{j+1}^*, \ldots, \theta_M^*\right)^\top,$$

where $\varepsilon_k \in \{-20\%, -10\%, 0\%, 10\%, 20\%\}$ denotes the perturbation level ($k = 1, \ldots, 5$). For each perturbed parameter set, the average coefficient of determination is computed as:

$$\hat{R}^2(\boldsymbol{\theta}^{(j,k)}) = \frac{1}{N}\sum_{i=1}^{N} R_i^2(\boldsymbol{\theta}^{(j,k)}),$$

where $N = 6$ corresponds to the six experimental data types fitted: tumor volume, cytotoxic T cell count, helper T cell count, regulatory T cell count, progenitor exhausted T cell proportion, and terminally exhausted T cell proportion. To quantify the impact of each parameter perturbation on the fitting performance, we define the comprehensive sensitivity index (CSI_j) for the j-th parameter as the range of the average R^2 values across all perturbation levels:

$$\mathrm{CSI}_j = \max_k \hat{R}^2(\boldsymbol{\theta}^{(j,k)}) - \min_k \hat{R}^2(\boldsymbol{\theta}^{(j,k)}).$$

A larger comprehensive sensitivity index value indicates that the overall model fitting performance is more sensitive to perturbations in that specific parameter.

The computational results indicate that the tumor proliferation rate (α_C), regulatory T cell proliferation rate (α_{T_r}), helper T cell death rate (d_{T_h}), and regulatory T cell death rate (d_{T_r}) exert a substantial influence on the overall model fitting performance (see Figure A). Across the perturbation range of -20% to $+20\%$, the comprehensive sensitivity index for these parameters exceeds 0.1. In other words, the model fitting quality is highly sensitive to perturbations in these parameters. Therefore, in model application and parameter estimation, priority should be given to ensuring the accuracy of these highly sensitive parameters, while parameters with low sensitivity can be simplified to reduce the complexity of model parameter estimation. At the baseline parameter values, the average coefficients of determination corresponding to α_C, α_{T_r}, d_{T_h}, and d_{T_r} all demonstrate good agreement with the experimental data (see Figure B).

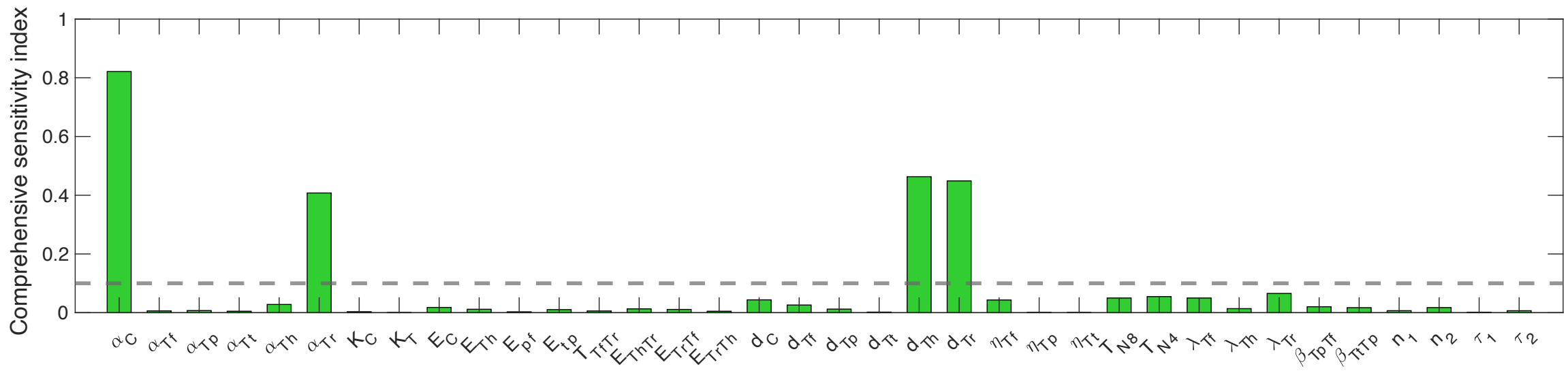

Figure A. Comprehensive sensitivity index for individual parameter perturbations. The comprehensive sensitivity index for each parameter is calculated as the range of $\hat{R}^2$ over five perturbation levels (-20% to $+20\%$).

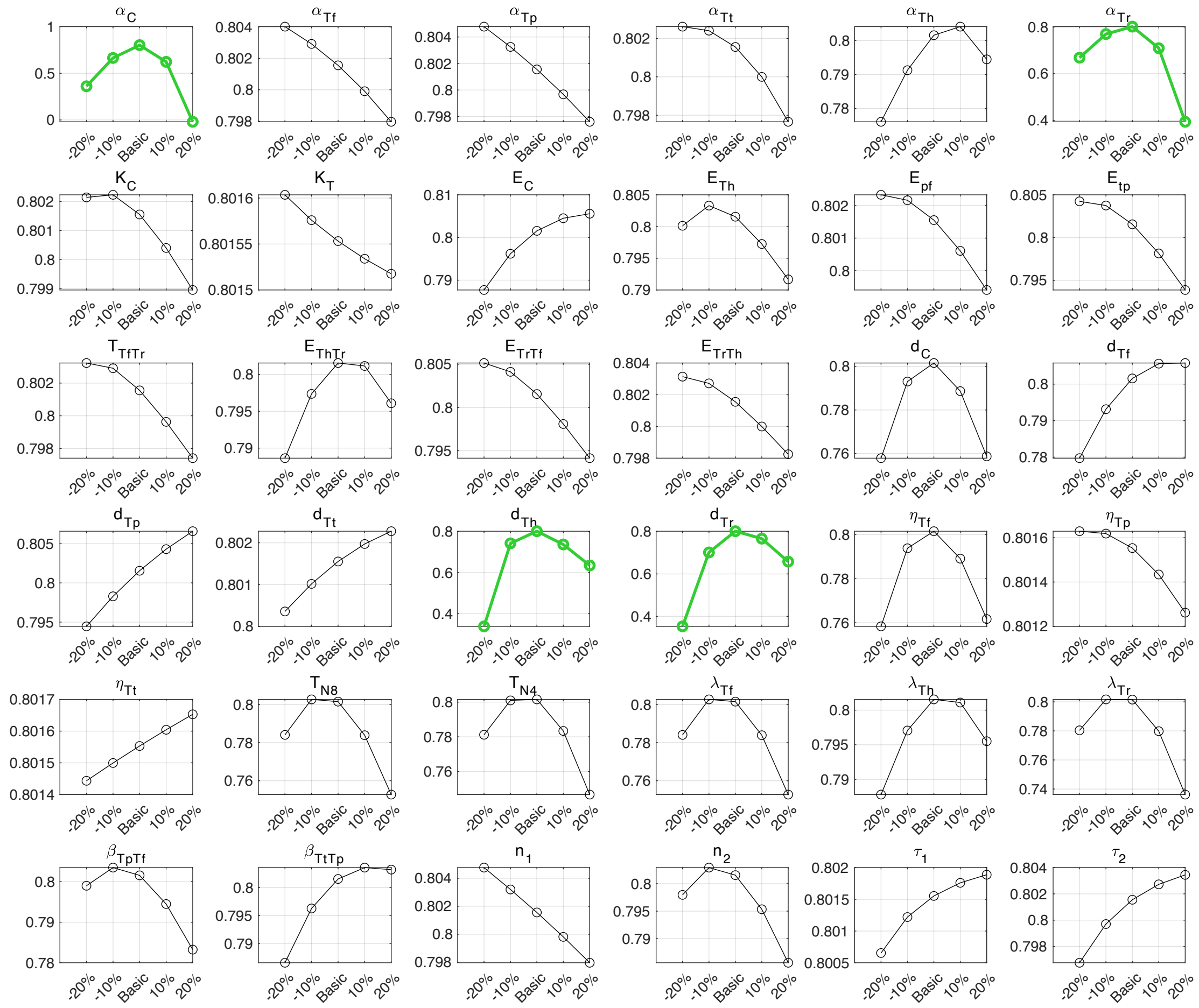

Figure B. Average coefficients of determination at perturbation levels of -20%, -10%, 0%, 10%, and 20% relative to baseline values.

To evaluate the influence of individual parameter perturbations on model fitting performance, we perturbed its value by $-20\%, -10\%, 0\%, 10\%, 20\%$ while keeping all other parameters at their baseline values. For each perturbed parameter set, we computed the coefficient of determination separately for each of the six experimental datasets. The difference between the maximum and minimum values across the perturbation levels was then calculated to characterize the sensitivity of the fitting performance to perturbations in that specific parameter. We set 0.1 as the sensitivity threshold for identifying influential parameters. Parameters with a range exceeding 0.1 were considered to have a significant impact on the fitting performance for the corresponding dataset.

The calculation results are shown in Figure C, where α_C, d_C, and η_{T_f} exert a substantial influence on the fitting of tumor volume data. For cytotoxic T cell counts, the parameters α_C, d_C, η_{T_f}, T_{N8}, λ_{T_f}, and $\beta_{T_pT_f}$ show pronounced effects. The fitting of helper T cell counts is notably affected by changes in α_C, α_{T_h}, and d_{T_h}. For regulatory T cell counts, α_C, α_{T_r}, d_{T_r}, T_{N4}, and λ_{T_r} play major roles. The fitting of progenitor exhausted T cell proportion is significantly influenced by α_C, E_{tp}, d_C, η_{T_f}, $\beta_{T_tT_p}$, and n_2. Finally, the fitting of terminally exhausted T cell proportion is primarily driven by variations in α_C alone.

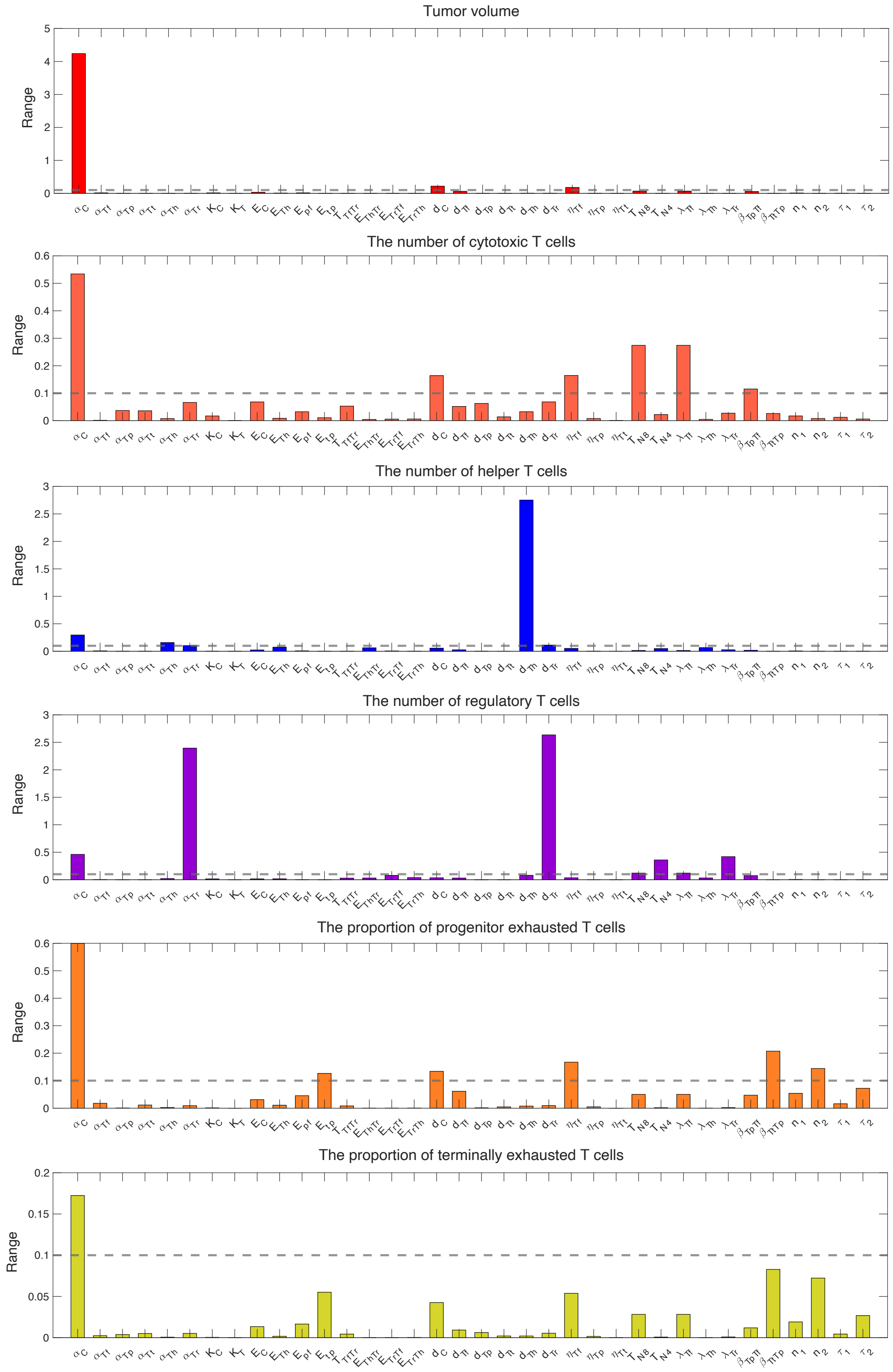

Figure C. The range of R^2 values across perturbation levels of $-20\%, -10\%, 0\%, 10\%$, and 20% relative to baseline. A larger range indicates that the corresponding dataset is more sensitive to the perturbed parameter, thereby providing greater informational value for parameter identification.

(2) Local sensitivity analysis. In differential equation-based dynamical models, parameters often carry specific biological or physical meanings. Uncertainties in parameter values may affect the system to varying degrees. To identify key factors influencing population dynamics, we selected eight classes of dynamically critical parameters from the untreated TIED model for local sensitivity analysis. For each selected parameter, we increased or decreased its baseline value by 10% and quantified the sensitivity using the following index:

$$S(\theta) = \frac{1}{T}\int_0^T \frac{x(\theta+\Delta\theta;t) - x(\theta;t)}{x(\theta;t)}\mathrm{d}t, \tag{1}$$

where θ denotes the model parameter of interest, $\Delta\theta$ represents a small perturbation applied to the parameter, x is the system output of interest, and $T = 20$ days is the observation period. This index reflects the average relative change in the system output induced by a small perturbation in parameter θ over the time interval $[0, T]$. A larger $|S(\theta)|$ indicates that the system is more sensitive to that parameter, whereas a smaller $|S(\theta)|$ suggests insensitivity to parameter variations. The calculation results are described in Figure D.

The results show that a higher tumor proliferation rate (α_C) promotes the synchronous expansion of both tumor volume and immune cell counts. Conversely, increasing the killing rate of T_f on C (η_{T_f}) reduces both tumor volume and immune cell numbers. Similarly, an increase in the activation rate of T_f (λ_{T_f}) helps control tumor growth and upregulates CD8+ T cell infiltration into the tumor. In contrast, upregulation of the precursor exhaustion rate ($\beta_{T_pT_f}$) and terminal exhaustion rate ($\beta_{T_tT_p}$) drives downregulation of T_f and T_p, respectively, ultimately leading to an expansion of T_t. Finally, the tumor carrying capacity (K_C) and the killing rates of exhausted T cells on tumors (η_{T_p}, η_{T_t}) are insensitive to small perturbations.

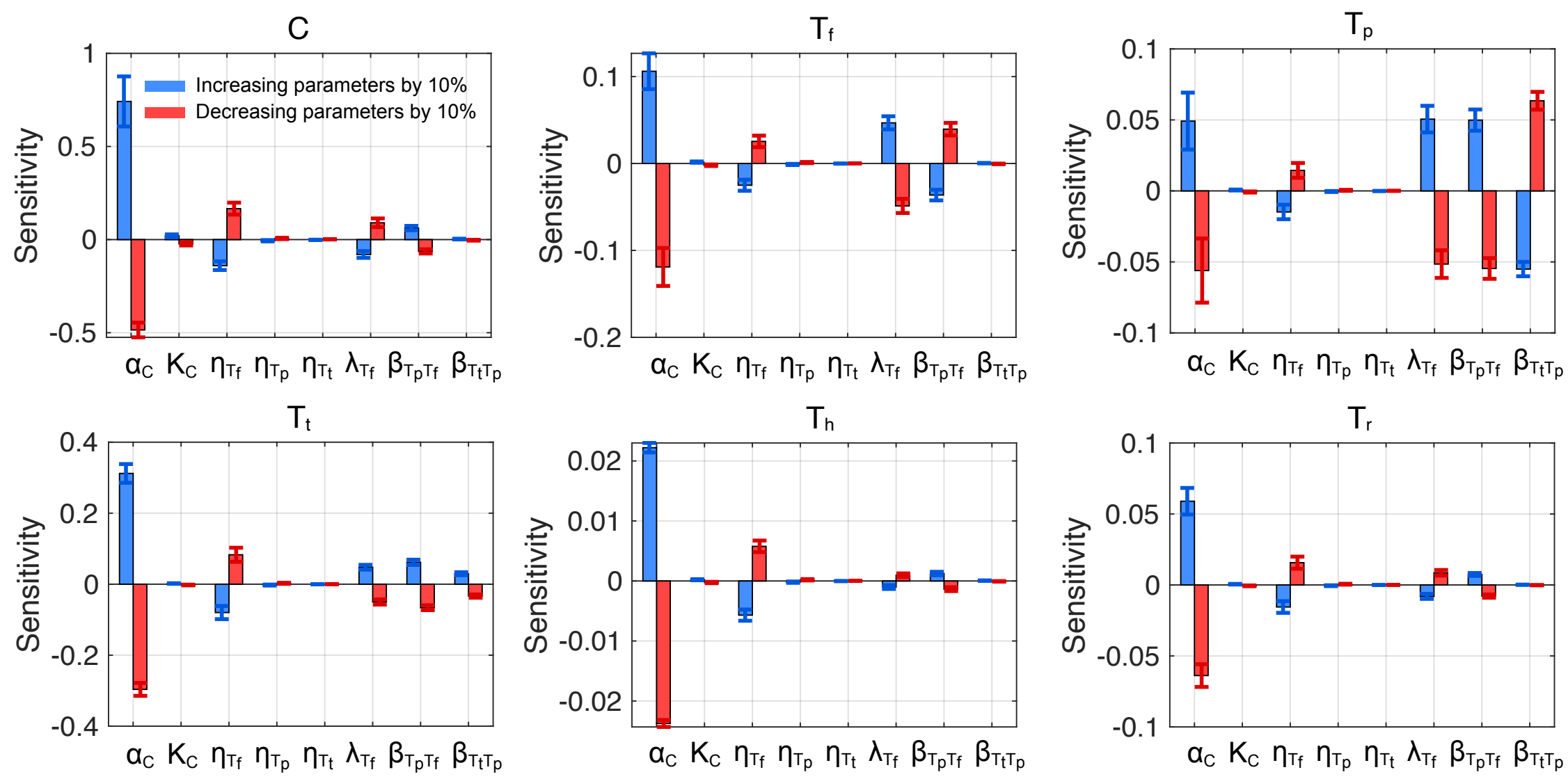

Figure D. Local sensitivity analysis. Evaluation of the impact of small perturbations in model parameters on tumor volume and immune cell counts. Bars represent the mean, and error bars indicate the standard deviation.

(3) Global sensitivity analysis. To further evaluate the impact of model parameters on model outputs under interacting conditions, we employed a global sensitivity analysis based on the Sobol method. This approach quantifies the various orders of sensitivity indices of the

aforementioned parameters on tumor volume and immune cell counts [1, 2]. Consider a biological system represented by the function $y = f(\boldsymbol{x})$, where the input is a vector of independent parameters $\boldsymbol{x} = [x_1, x_2, \cdots, x_k]$. The parameter space is defined as the k-dimensional unit hypercube:

$$\Omega = (\boldsymbol{x} | 0 \leq x_i \leq 1; i = 1, ..., k). \tag{2}$$

Assume that $f(\boldsymbol{x})$ is a square-integrable function defined over the k-dimensional unit hypercube Ω. It can be uniquely decomposed into summands of increasing dimensionality, known as the ANOVA (analysis of variance) decomposition:

$$f(\boldsymbol{x}) = f_0 + \sum_{i=1}^{k} f_i(x_i) + \sum_{1 \leq i < j \leq k} f_{i,j}(x_i, x_j) + \cdots + f_{1,2,\ldots,k}(x_1, x_2, \ldots, x_k), \tag{3}$$

where f_0 is a constant term representing the overall mean of f. For this decomposition to be orthogonal, the component functions must satisfy the following conditions:

$$\int_0^1 f_{i_1,\ldots,i_s}(x_{i_1}, \ldots, x_{i_s}) \, \mathrm{d}x_{i_q} = 0, \quad 1 \leq q \leq s, \tag{4}$$

$$\int_\Omega f(\boldsymbol{x}) \, \mathrm{d}\boldsymbol{x} = f_0, \tag{5}$$

$$\int_\Omega f_{i_1,\ldots,i_s}(x_{i_1}, \ldots, x_{i_s}) \, f_{j_1,\ldots,j_l}(x_{j_1}, \ldots, x_{j_l}) \, \mathrm{d}\boldsymbol{x} = 0, \tag{6}$$

where $\mathrm{d}\boldsymbol{x} = \mathrm{d}x_1 \cdots \mathrm{d}x_k$. The total variance $V(y)$ and partial variance $V_{i_1,i_2,\ldots,i_s}$ of $f(\boldsymbol{x})$ can be calculated using the following equation,

$$V(y) = \int_\Omega f^2(\boldsymbol{x}) \, d\boldsymbol{x} - f_0^2, \tag{7}$$

$$V_{i_1,i_2,\ldots,i_s} = \int_0^1 \cdots \int_0^1 f^2_{i_1,i_2,\ldots,i_s}(x_{i_1}, \ldots, x_{i_s}) \, dx_{i_1} \cdots dx_{i_s}. \tag{8}$$

Therefore, the total variance of the model output can be decomposed into the sum of variances contributed by each term in the ANOVA decomposition:

$$V(y) = \sum_{i=1}^{k} V_i + \sum_{1 \leq i < j \leq k} V_{ij} + \ldots + V_{1,2,3,\ldots,k}, \tag{9}$$

where V_i, V_{ij}, and $V_{1,2,3,\ldots,k}$ represent the partial variances associated with the component functions $f_i(x_i)$, $f_{i,j}(x_i, x_j)$, and $f_{1,2,\ldots,k}(x_1, x_2, \ldots, x_k)$, respectively. Based on this variance decomposition, the Sobol sensitivity indices are defined as follows:

$$S_i = \frac{V_i}{V(y)}, \quad S_{ij} = \frac{V_{ij}}{V(y)}, \quad T_i = \frac{V(y) - V_{\sim i}}{V(y)}, \tag{10}$$

where $V_{\sim i}$ denotes the sum of all partial variances that do not involve the parameter x_i. The sensitivity indices have the following interpretations:

- S_i is the first-order sensitivity index, which measures the individual contribution of parameter x_i alone to the total output variance.

- S_{ij} is the second-order sensitivity index, which quantifies the contribution due to the interaction between parameters x_i and x_j.
- T_i is the total-order sensitivity index, which captures the overall contribution of parameter x_i to the output variance, including its first-order effect and all higher-order interactions involving x_i.

The computational results reveal that η_{T_f} is the most critical factor regulating C and T_h, exhibiting both high total-order and first-order sensitivity indices (see Figure E). This indicates that the cytotoxic capacity of functionally intact T cells serves as a core mechanism for suppressing tumor growth, while also being closely associated with the auxiliary immune response. Furthermore, $\beta_{T_pT_f}$ and λ_{T_f} demonstrate high total-order and first-order sensitivities for T_f, T_p, T_t, and T_r. This suggests that intervening in the processes of T cell activation and exhaustion represents a critical biological window for modulating immune cell functional lineages and therapeutic responses. The second-order sensitivity results indicate strong interaction effects among α_C, $\beta_{T_tT_p}$, η_{T_p}, and η_{T_t} (see Figure E). This finding implies that tumor growth dynamics and T cell exhaustion processes are not independent but rather collectively influence system behavior through nonlinear coupling. Such higher-order interactions suggest that simultaneously targeting T cell exhaustion while rescuing their cytotoxic function may yield synergistic therapeutic effects beyond those achieved by single interventions.

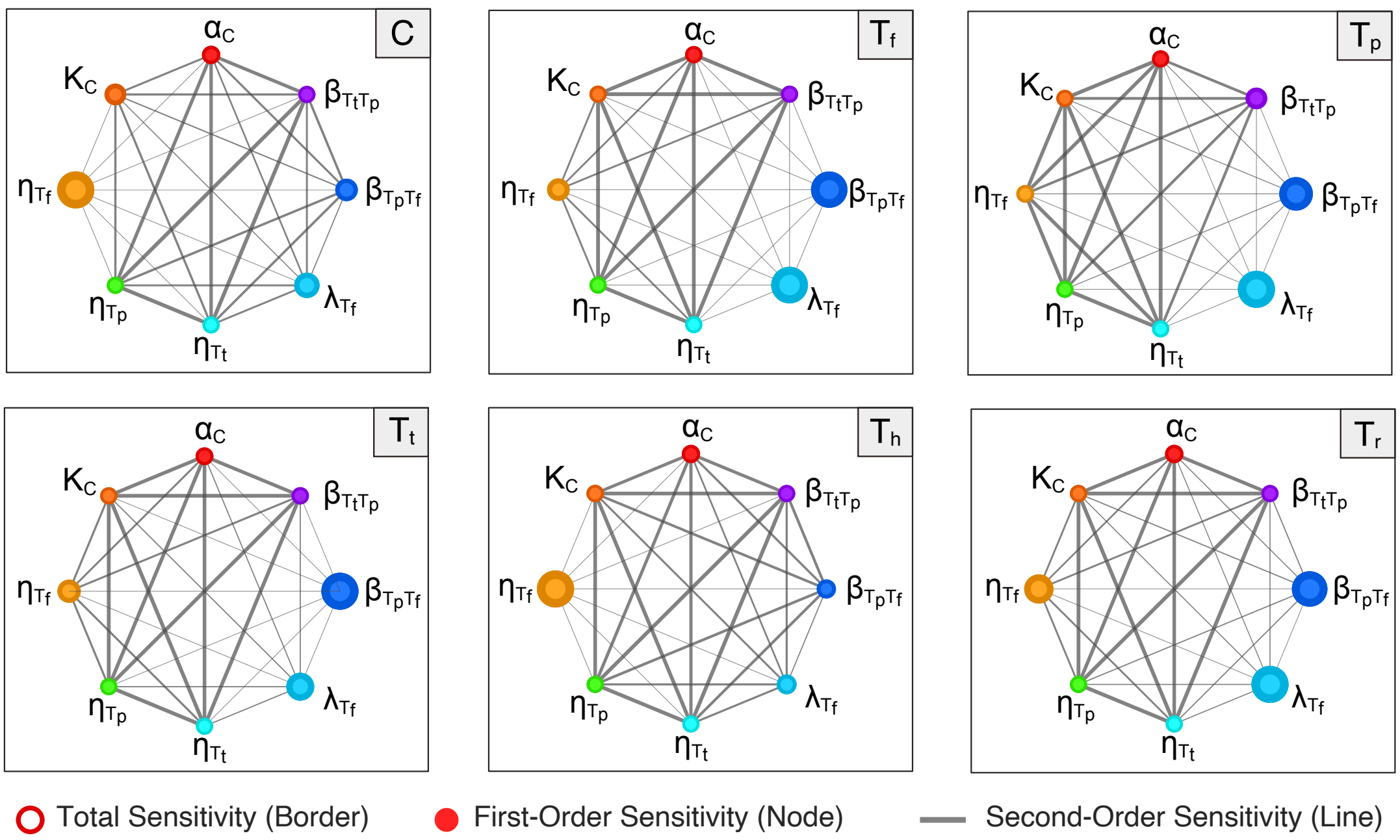

Figure E. Global sensitivity analysis based on the Sobol method. Nodes represent first-order sensitivity indices, borders indicate total-order sensitivity indices, and edges denote second-order sensitivity indices.

(4) Correlation analysis. To investigate how the association patterns between model parameters and system variables change under different treatment strategies, we performed a hierarchical clustering-based Pearson correlation analysis on the generated virtual cohort (see Figure F). In the control group, λ_{T_f} exhibited strong positive correlations with T_f, T_p, T_t, and T_h, indicating that enhanced T cell activation capacity drives significant expansion of anti-tumor T cell subsets. Meanwhile, an increase in η_{T_f} suppressed tumor growth, consequently downregulating

the activation level of the immune system. Furthermore, a higher $\beta_{T_pT_f}$ promoted the expansion of T_p and T_r while downregulating T_f. This suggests that accelerated T cell exhaustion not only impairs immune effector function but also facilitates the formation of an immunosuppressive microenvironment. In the anti-PD-1 monotherapy group, the negative correlations between η_{T_f} and both C and T_r were significantly strengthened. Additionally, the anti-PD-1 treatment intensity γ_2 showed strong negative correlations with T_p and T_t. These findings indicate that anti-PD-1 therapy helps delay the progression of T cell exhaustion. In the combination therapy group (anti-PD-1 + anti-CTLA-4), the negative correlation between $\beta_{T_pT_f}$ and C was attenuated, while the anti-CTLA-4 treatment intensity γ_1 exhibited a negative correlation with C. This suggests that CTLA-4 blockade contributes more directly to tumor suppression within the combination regimen.

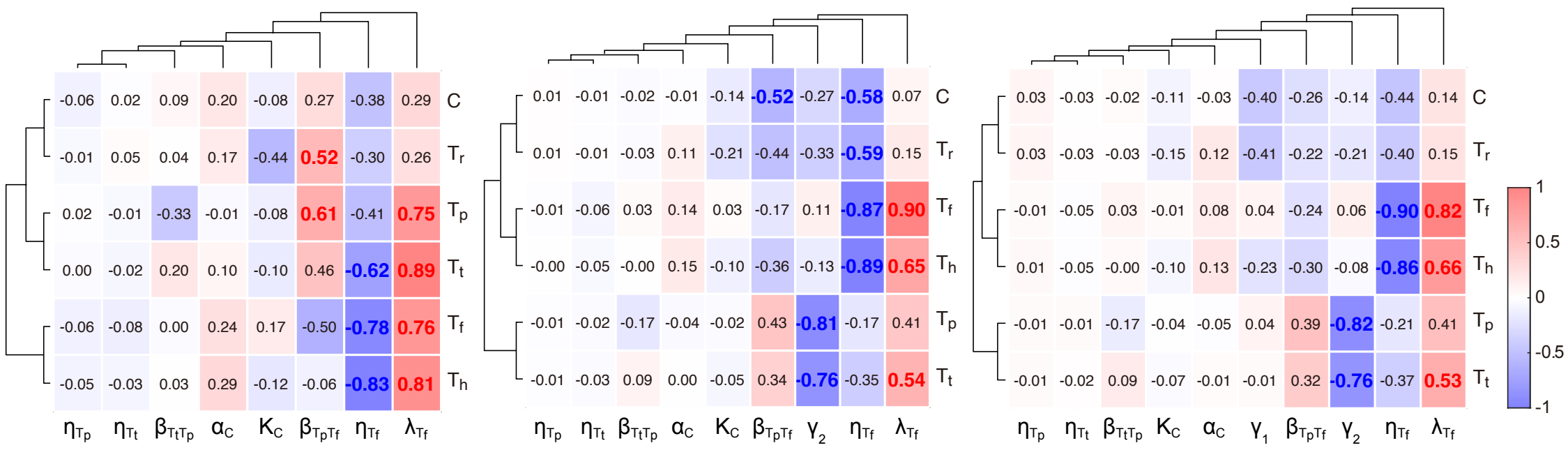

Figure F. Correlation analysis of model parameters and variables under control, anti-PD-1, and anti-PD-1 + anti-CTLA-4 conditions.

References

[1] Andrea Saltelli. Making best use of model evaluations to compute sensitivity indices. *Comput Phys Commun*, 145(2):280–297, May 2002.

[2] I.M Sobol′. Global sensitivity indices for nonlinear mathematical models and their Monte Carlo estimates. *Math Comput Simulat*, 55(1–3):271–280, February 2001.
