# Supplementary material for: Multiscale modeling of T cell exhaustion: A mathematical framework integrating continuous dynamics with spatial heterogeneity
Source: PLoS Comput Biol. 2026 Aug 26;22(8):e1014690. doi: 10.1371/journal.pcbi.1014690 (PMC13541251; doi:10.1371/journal.pcbi.1014690)
Supplement: Supplementary file 6 [file pcbi.1014690.s006.pdf]

S6 Appendix: Exhaustion level analysis

To further characterize T cell exhaustion levels across different datasets, we analyzed single-cell transcriptomic data from the colorectal cancer dataset GSE108989 and the melanoma dataset GSE115978. Based on the clustering results for each dataset (see Figure A), we calculated exhaustion scores for individual CD8+ T cells using the AddModuleScore algorithm. The results show that in both independent datasets, exhaustion scores increase progressively along the trajectory from naïve CD8+ T cells (T_n) to tumor-reactive T cells (T_f), progenitor exhausted T cells (T_p), and terminally exhausted T cells (T_t) (see Figure B), further validating the continuous exhaustion lineage proposed in this study. This finding suggests that the exhaustion process of CD8+ T cells follows a similar continuous dynamic pattern regardless of tumor type.

We defined the exhaustion weight δ_i for cell type i via min-max normalization:

$$\delta_i = \frac{s_i - s_{\text{min}}}{s_{\text{max}} - s_{\text{min}}},$$

where s_i denotes the raw average exhaustion score, and s_{min} and s_{max} are the minimum and maximum raw scores across all cell types. In the colorectal cancer dataset GSE108989, the average exhaustion scores for T_f, T_p, and T_t are -0.332, 0.135, and 0.555, respectively (see Figure C), yielding normalized weights of 0, 0.527, and 1. In the melanoma dataset GSE115978, the average exhaustion scores for T_f, T_p, and T_t are -0.253, 0.104, and 0.231, respectively (see Figure C), yielding normalized weights of 0, 0.738, and 1, respectively.

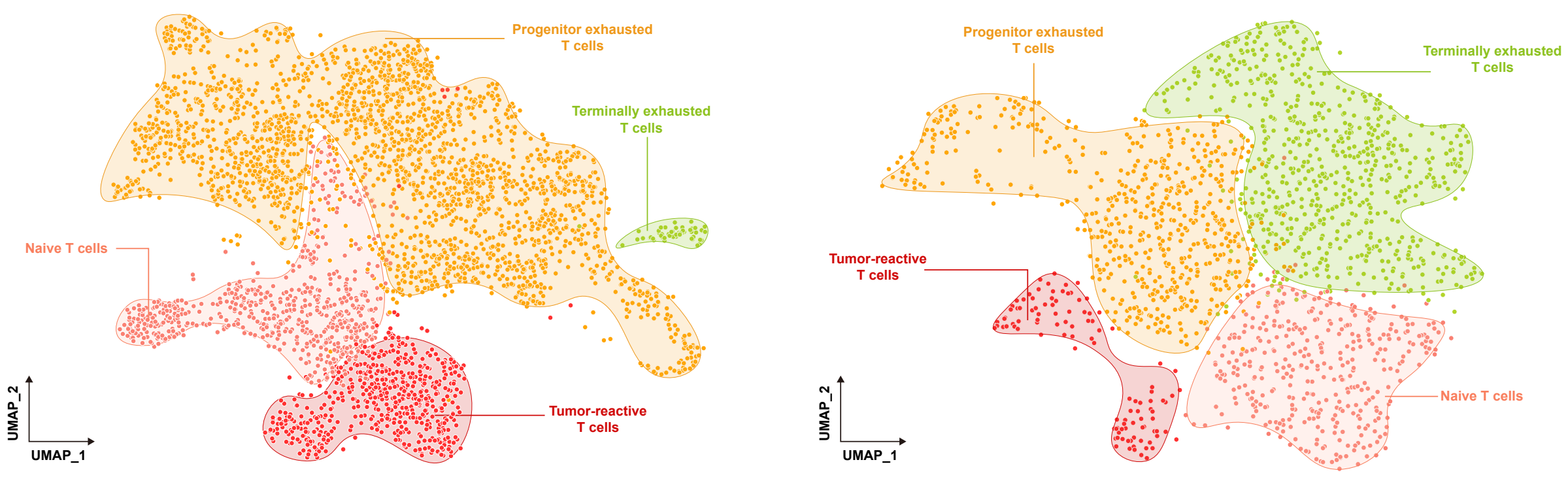

Figure A. Cluster analysis of GSE108989 (colorectal cancer, left panel) and GSE115978 (melanoma, right panel).

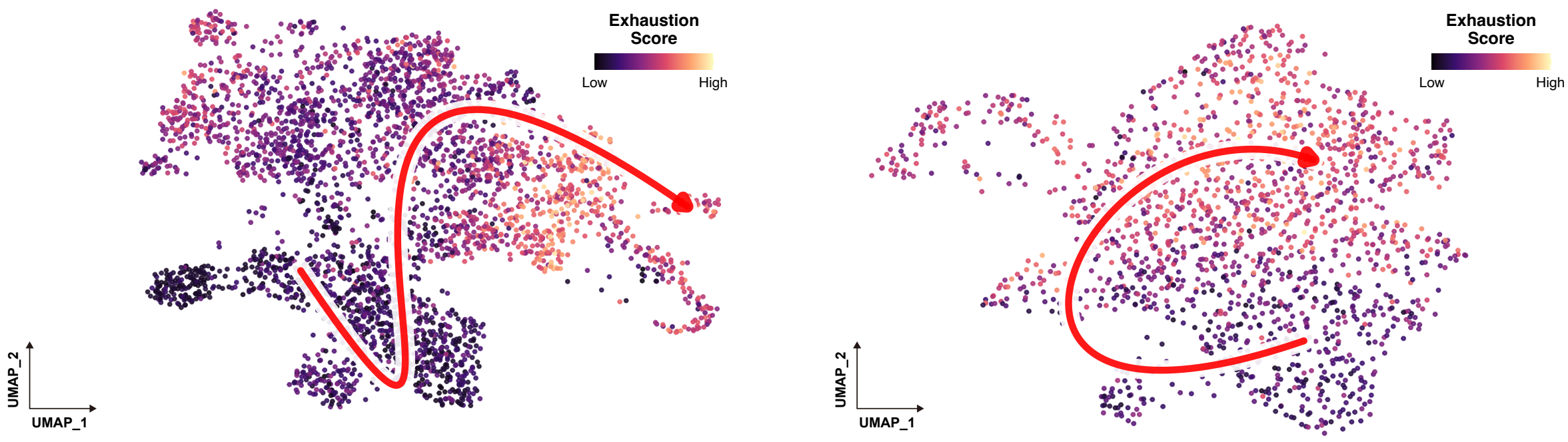

Figure B. Single-cell exhaustion scores of GSE108989 (colorectal cancer, left panel) and GSE115978 (melanoma, right panel).

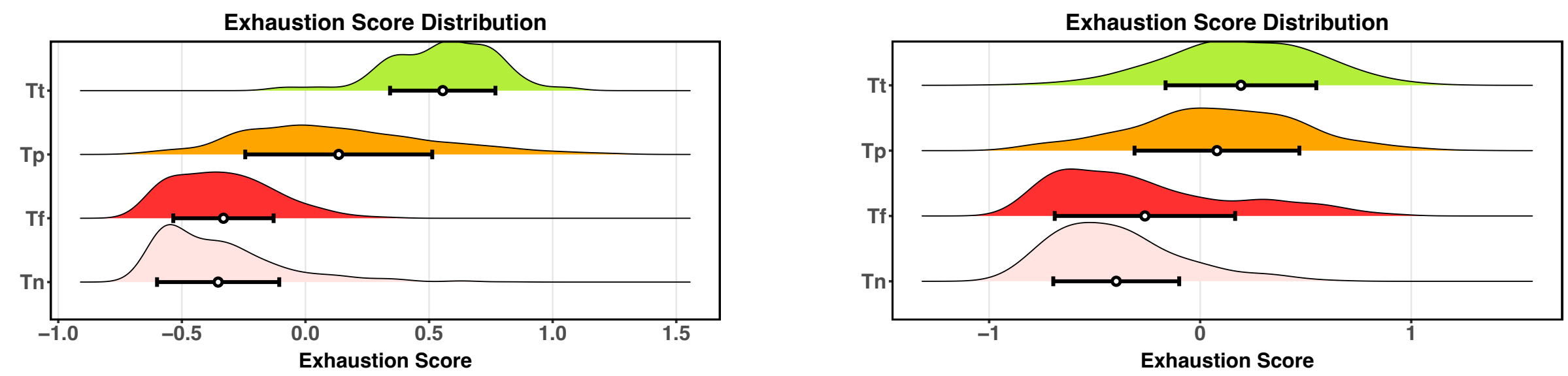

Figure C. Exhaustion score distribution of GSE108989 (colorectal cancer, left panel) and GSE115978 (melanoma, right panel).

In our framework, the exhaustion level is quantified as $Q(t) = \boldsymbol{\delta} \cdot \boldsymbol{x}(t) = \sum_{i=1}^{3} \delta_i \cdot x_i(t)$, with the weight δ_i defined via min-max normalization. Variations in exhaustion levels across different datasets result in inconsistent normalized exhaustion weights (δ_2) for T_p. Since δ_2 ranges between 0 and 1, we selected five representative gradients (0.1, 0.3, 0.5, 0.7, 0.9) in the control, anti-PD-1, and anti-PD-1+anti-CTLA-4 groups to systematically analyze the dynamic evolution of exhaustion levels under different weight values. The results show that as δ_2 increases, the average exhaustion levels increase across all treatment regimens (see Figure D). This difference in exhaustion levels is most pronounced in the control group (see Figure E). In the anti-PD-1 monotherapy and anti-PD-1+anti-CTLA-4 combination therapy groups, the intergroup differences in exhaustion levels are relatively significant at early time points (days 5 and 10) but gradually diminish at later time points (days 15 and 20) (see Figure E). This trend suggests that the exhaustion weight of T_p is a key determinant of the overall systemic exhaustion level, and that immunotherapy can partially attenuate the driving effect of this weight on the exhaustion process.

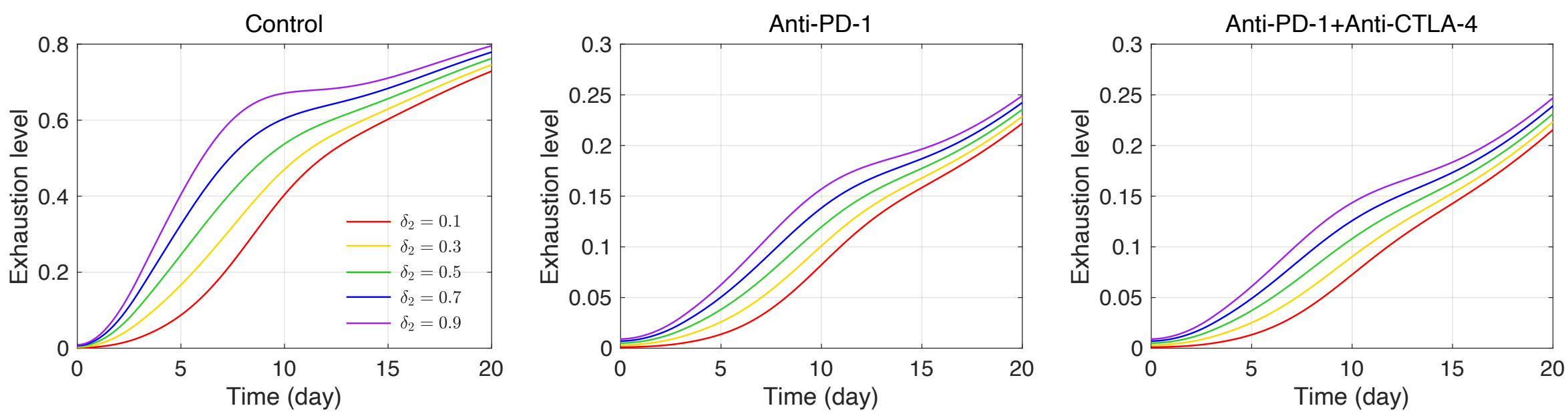

Figure D. Temporal evolution of exhaustion levels under different progenitor exhaustion weights (δ_2) in the control, anti-PD-1, and anti-PD-1+anti-CTLA-4 groups.

To further investigate changes in the immune system under different T_p exhaustion weights (δ_2), we plotted the temporal evolution of the pseudo-potential of the immune system as a function of the percentage of tumor-reactive T cells and the exhaustion level. The results indicate that smaller δ_2 values allow the system to maintain a low exhaustion state, whereas larger δ_2 values (e.g., $\delta_2 = 0.9$) tend to drive the system toward higher exhaustion levels (see Figure F). In other words, larger δ_2 values (e.g., $\delta_2 = 0.9$) make the relationship between the percentage of tumor-reactive T cells and the exhaustion level more linear (see Figure F).

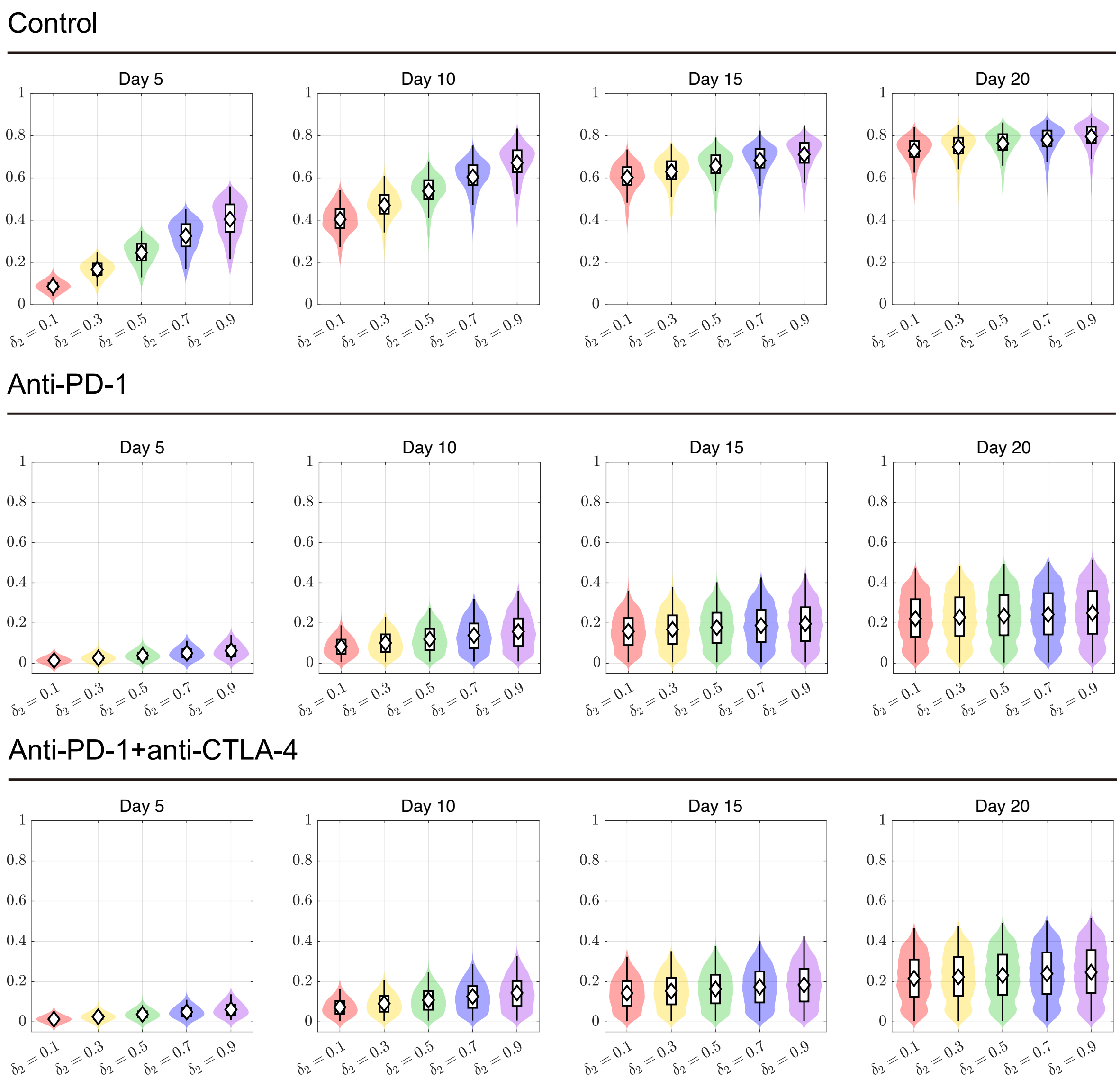

Figure E. Boxplots showing the distribution of exhaustion levels at days 5, 10, 15, and 20 under different progenitor exhaustion weights (δ_2) in the control, anti-PD-1, and anti-PD-1+anti-CTLA-4 groups.

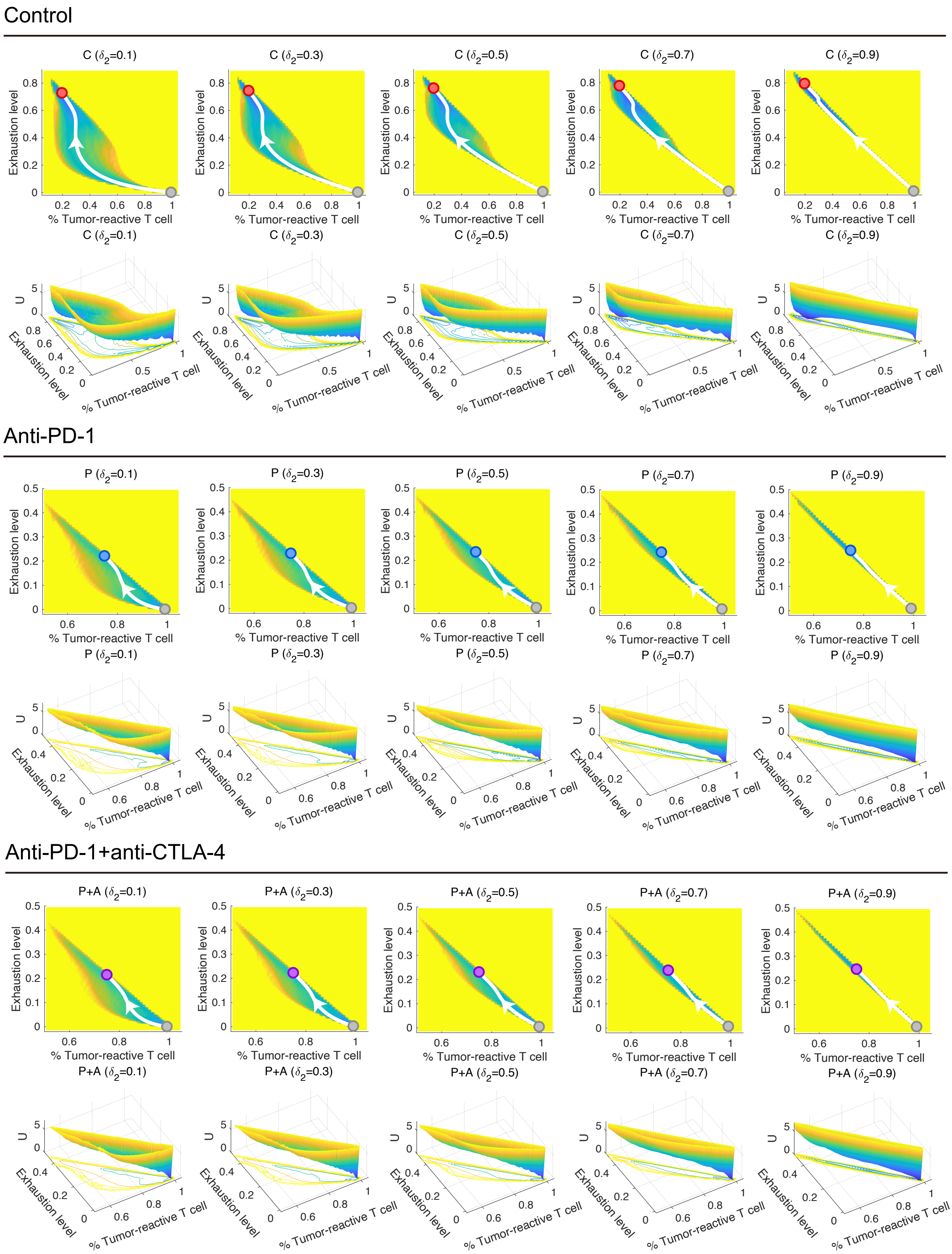

Figure F. Temporal evolution of the immune system's pseudo-potential energy under different progenitor-exhaustion weights (δ_2) for the control, anti-PD-1, and anti-PD-1+anti-CTLA-4 groups. C: control; P: anti-PD-1 monotherapy; P+A: anti-PD-1+anti-CTLA-4.
