# Supplementary material for: Multiscale modeling of T cell exhaustion: A mathematical framework integrating continuous dynamics with spatial heterogeneity
Source: PLoS Comput Biol. 2026 Aug 26;22(8):e1014690. doi: 10.1371/journal.pcbi.1014690 (PMC13541251; doi:10.1371/journal.pcbi.1014690)
Supplement: Supplementary file 7 [file pcbi.1014690.s007.pdf]

S7 Appendix. Delay dynamic analysis

To explore the regulatory effects of T cell exhaustion-related time-delay parameters τ_1 and τ_2 on the evolution of the tumor-immune system, single-parameter numerical simulations were performed. Figures A and B illustrate the dynamical profiles of tumor cells, tumor-reactive T cells, progenitor exhausted T cells, terminally exhausted T cells, helper T cells, and regulatory T cells under varying values of τ_1 and τ_2, respectively. The results show that a larger τ_1 (time delay for progenitor exhaustion) facilitates tumor progression, reduces the abundance of tumor-reactive T cells, and elevates the numbers of progenitor exhausted T cells, terminally exhausted T cells, and regulatory T cells (Figure A). Similarly, increased τ_2 (time delay for terminal exhaustion) accelerates tumor growth but markedly decreases progenitor exhausted T cells and accumulates terminally exhausted T cells (Figure B).

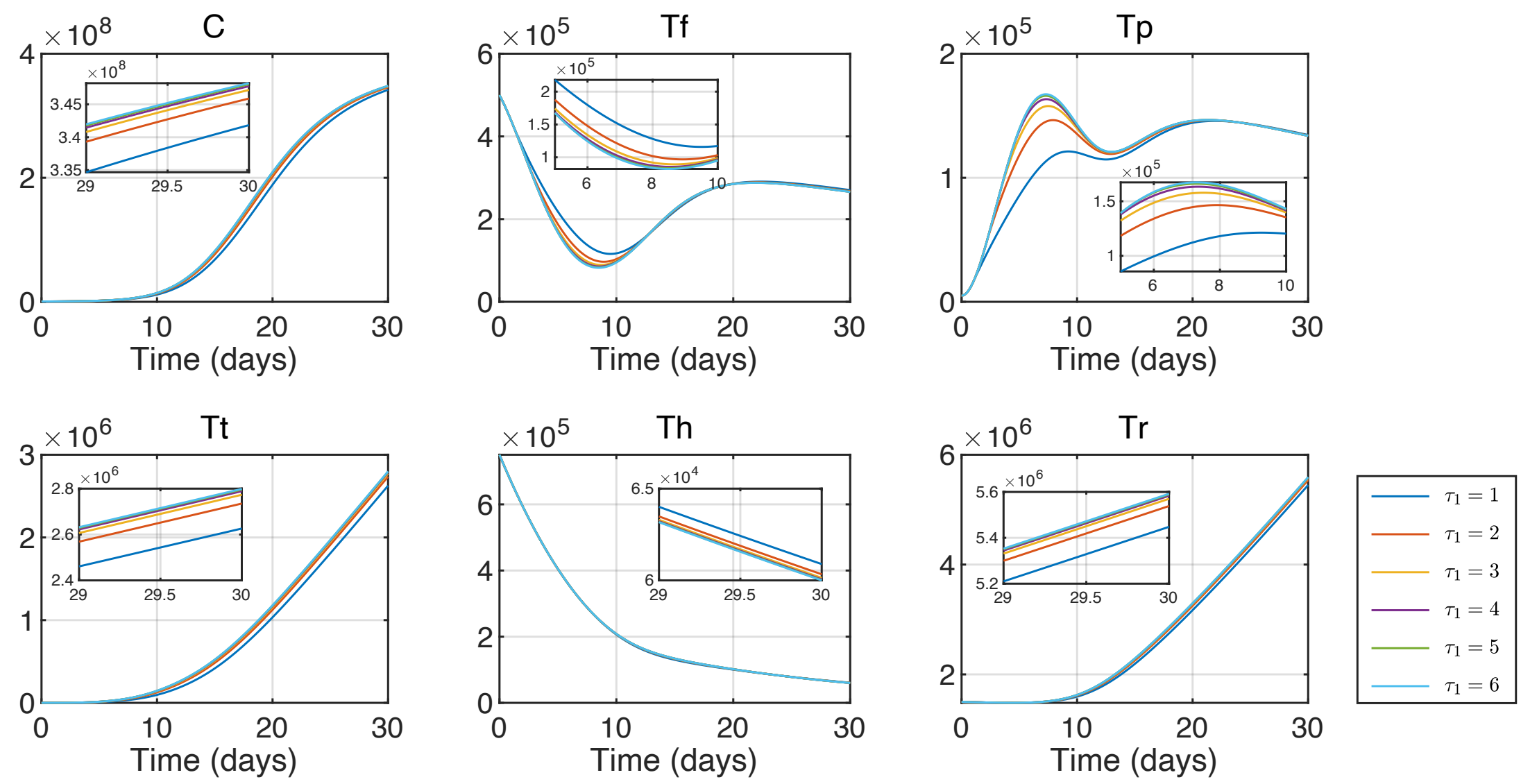

Figure A. Dynamics of the tumor-immune system under different τ_1 values.

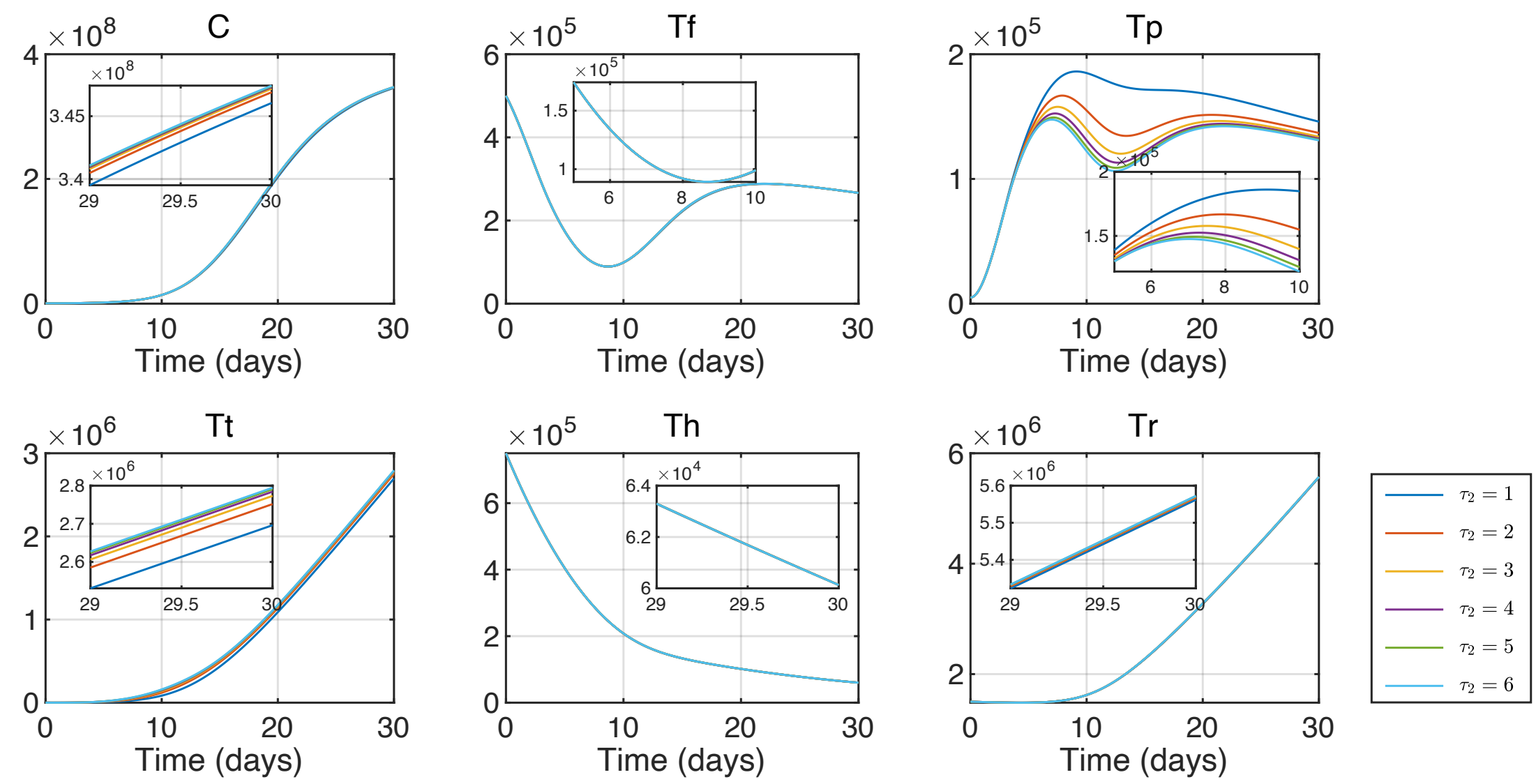

Figure B. Dynamics of the tumor-immune system under different τ_2 values.
