# Supplementary material for: Multiscale modeling of T cell exhaustion: A mathematical framework integrating continuous dynamics with spatial heterogeneity
Source: PLoS Comput Biol. 2026 Aug 26;22(8):e1014690. doi: 10.1371/journal.pcbi.1014690 (PMC13541251; doi:10.1371/journal.pcbi.1014690)
Supplement: Supplementary file 8 [file pcbi.1014690.s008.pdf]

S8 Appendix. ODE model integrating antigen exposure levels

To verify the rationality of using a finite-time-window integral to characterize cumulative antigen exposure in the original model, we constructed an ODE model that introduces cumulative antigen exposure Z. The original model employs a truncated integral $\int_{t-\tau}^{t} C(s)\,\mathrm{d}s$ to compute cumulative antigen, which only accounts for tumor antigens within the limited time interval $[t-\tau, t]$. In the extended model, we use $\frac{\mathrm{d}Z}{\mathrm{d}t} = C - \frac{Z}{\tau}$ to describe the dynamic process of cumulative antigen exposure. Here, C represents the instantaneous tumor cell count, which denotes the amount of newly generated antigen input per unit time. The term Z/τ represents the natural clearance loss of antigen, with τ denoting the mean residence time of antigen. Assuming $Z(0) = 0$, we have $Z(t) = \int_0^t C(s)e^{-(t-s)/\tau}ds$. We set $\tau = \tau_1 = \tau_2$, so that the integral expressions in the two exhaustion terms can be replaced by a single variable Z, while the remaining structure of the model remains unchanged. Based on the above, an ODE model incorporating the level of antigen exposure is established as:

$$
\begin{aligned}
\frac{\mathrm{d}C}{\mathrm{d}t} =& \alpha_C \left(1 - \frac{C}{K_C}\right) C - \left(\eta_{T_f} T_f + \eta_{T_p} T_p + \eta_{T_t} T_t\right) C - d_C C, \\
\frac{\mathrm{d}Z}{\mathrm{d}t} =& C - Z/\tau, \\
\frac{\mathrm{d}T_f}{\mathrm{d}t} =& \lambda_{T_f} \left(1 + \gamma_1\right) \cdot \frac{C}{E_C + C} \cdot \frac{1}{1 + T_r/E_{T_f T_r}} \cdot T_{N8} + \alpha_{T_f} \frac{T_h}{T_h + E_{T_h}} \left(1 - \frac{T_f + T_p + T_t}{K_T}\right) T_f \\
& - \beta_{T_p T_f} \left(1 - \gamma_2\right) \cdot \frac{Z^{n_1}}{E_{pf}^{n_1} + Z^{n_1}} \cdot T_f - d_{T_f} T_f, \\
\frac{\mathrm{d}T_p}{\mathrm{d}t} =& \alpha_{T_p} \left(1 - \frac{T_f + T_p + T_t}{K_T}\right) T_p + \beta_{T_p T_f} \left(1 - \gamma_2\right) \cdot \frac{Z^{n_1}}{E_{pf}^{n_1} + Z^{n_1}} \cdot T_f \\
& - \beta_{T_t T_p} \cdot \frac{Z^{n_2}}{E_{tp}^{n_2} + Z^{n_2}} \cdot T_p - d_{T_p} T_p, \\
\frac{\mathrm{d}T_t}{\mathrm{d}t} =& \alpha_{T_t} \left(1 - \frac{T_f + T_p + T_t}{K_T}\right) T_t + \beta_{T_t T_p} \cdot \frac{Z^{n_2}}{E_{tp}^{n_2} + Z^{n_2}} \cdot T_p - d_{T_t} T_t, \\
\frac{\mathrm{d}T_h}{\mathrm{d}t} =& \lambda_{T_h} \cdot \frac{C}{E_C + C} \cdot \frac{1}{1 + T_r/E_{T_h T_r}} T_{N4} + \alpha_{T_h} \frac{T_h}{E_{T_h} + T_h} T_h - d_{T_h} T_h, \\
\frac{\mathrm{d}T_r}{\mathrm{d}t} =& \lambda_{T_r} \cdot \frac{C}{E_C + C} \cdot \frac{1}{1 + T_f/E_{T_r T_f}} \cdot \frac{1}{1 + T_h/E_{T_r T_h}} \cdot T_{N4} + \alpha_{T_r} T_f - d_{T_r} T_r.
\end{aligned}
$$

Numerical results show that the R^2 values of tumor volume under control, anti-PD-1 monotherapy, and combined anti-PD-1 plus anti-CTLA-4 therapy are 0.96 vs 0.95, 0.83 vs 0.84, and 0.61 vs 0.62 for the IDE and ODE models (Figure A), respectively. For the dynamical profiles of cytotoxic T cells, helper T cells and regulatory T cells, the corresponding R^2 values equal 0.49 vs 0.52, 0.95 vs 0.94 and 0.68 vs 0.68 (Figure A). Accordingly, the IDE and ODE models exhibit comparable fitting performance for tumor volume, cytotoxic T cells, helper T cells and regulatory T cells, with R^2 fluctuations less than 0.03. Besides, no obvious discrepancy exists between the two models in fitting the fraction of terminally exhausted T cells (Figure A). In contrast, the IDE model achieves remarkably superior performance over the ODE model in reproducing the dynamics of progenitor exhausted T cells, with respective R^2 of 0.83 vs 0.69 (Figure A), indicating that the IDE model better captures the early exhaustion kinetics.

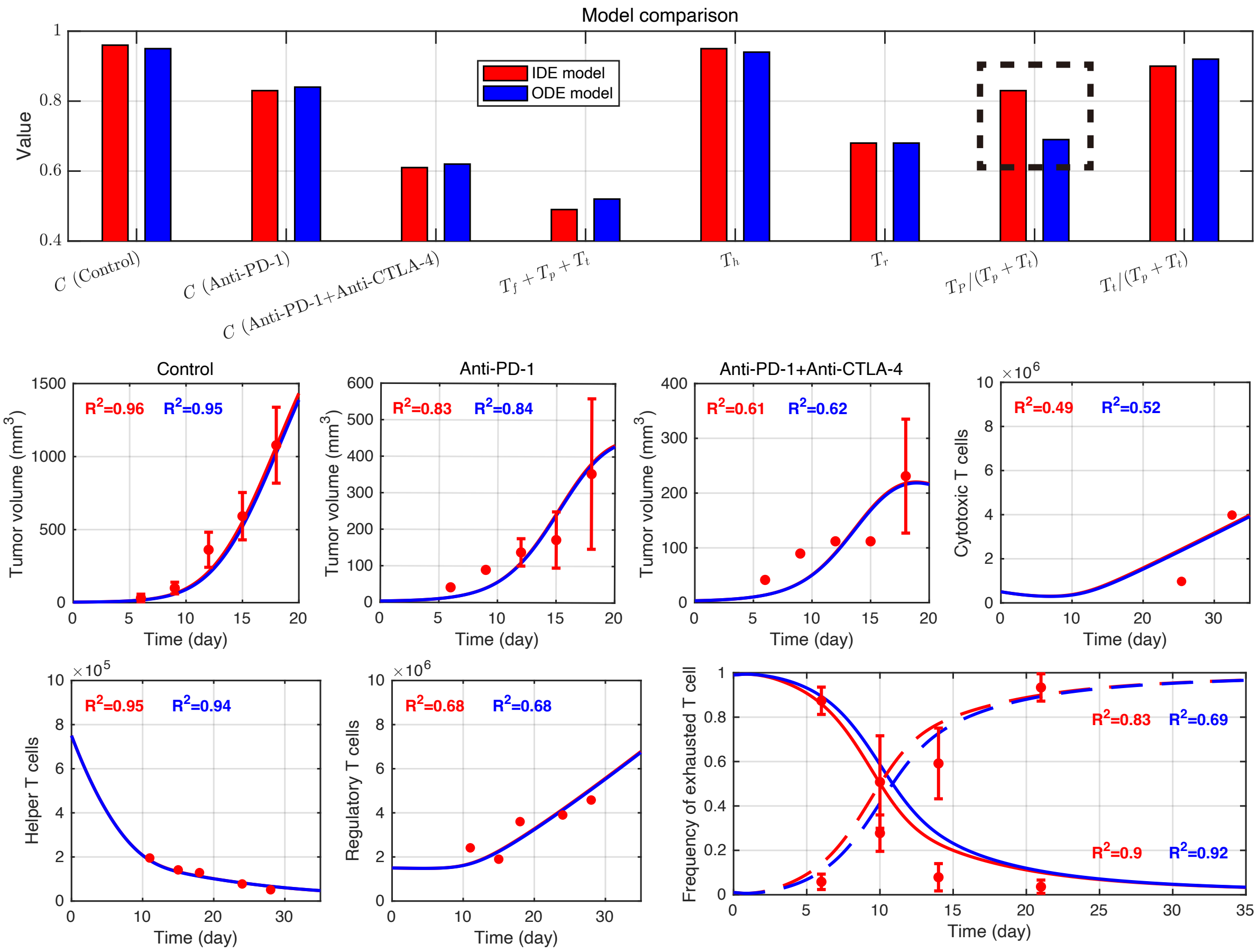

Figure A. Performance evaluation of the IDE model and the ODE model on data fitting. It presents the R^2 comparison and the temporal evolution of different indices for both models.
